# Identification of 170 New Long Noncoding RNAs in* Schistosoma mansoni*

**DOI:** 10.1155/2018/1264697

**Published:** 2018-07-11

**Authors:** Victor F. Oliveira, Lauro A. G. Moares, Ester A. Mota, Liana K. Jannotti-Passos, Paulo M. Z. Coelho, Ana C. A. Mattos, Flávia F. B. Couto, Brian E. Caffrey, Annalisa Marsico, Renata Guerra-Sá

**Affiliations:** ^1^Departamento de Ciências Biológicas, Núcleo de Pesquisas em Ciências Biológicas, Universidade Federal de Ouro Preto, Morro do Cruzeiro, Ouro Preto, MG, Brazil; ^2^Departamento de Computação, Laboratório de Computação de Sistemas Inteligentes, Universidade Federal de Ouro Preto, Morro do Cruzeiro, Ouro Preto, MG, Brazil; ^3^Centro de Pesquisas René Rachou, Fiocruz, Belo Horizonte, MG, Brazil; ^4^Max Planck Institute for Molecular Genetics, Ihnestr. 63-73, 14195 Berlin, Germany; ^5^Freie Universitaet Berlin, Arnimallee 14, 14195 Berlin, Germany

## Abstract

Long noncoding RNAs (lncRNAs) are transcripts generally longer than 200 nucleotides with no or poor protein coding potential, and most of their functions are also poorly characterized. Recently, an increasing number of studies have shown that lncRNAs can be involved in various critical biological processes such as organism development or cancer progression. Little, however, is known about their effects in helminths parasites, such as* Schistosoma mansoni*. Here, we present a computational pipeline to identify and characterize lncRNAs from RNA-seq data with high confidence from* S. mansoni* adult worms. Through the utilization of different criteria such as genome localization, exon number, gene length, and stability, we identified 170 new putative lncRNAs. All novel* S. mansoni* lncRNAs have no conserved synteny including human and mouse. These closest protein coding genes were enriched in 10 significant Gene Ontology terms related to metabolism, transport, and biosynthesis. Fifteen putative lncRNAs showed differential expression, and three displayed sex-specific differential expressions in praziquantel sensitive and resistant adult worm couples. Together, our method can predict a set of novel lncRNAs from the RNA-seq data. Some lncRNAs are shown to be differentially expressed suggesting that those novel lncRNAs can be given high priority in further functional studies focused on praziquantel resistance.

## 1. Introduction

The trematode* Schistosoma mansoni* is the primary parasite species responsible for schistosomiasis, a chronic debilitating disease. It is considered one of the most devastating tropical diseases in the world with at least 258 million people infected. Furthermore, 800 million people were living in endemic areas at risk of infection with more than 200,000 deaths each year [1, 2]. Its transmission has been reported in more than 78 countries, especially in tropical and subtropical areas such as Central and South America, Africa, and Southeast Asia [3, 4].

Although, in the last few decades, several drugs have been used for the treatment of schistosomiasis, with praziquantel (PZQ) representing the only widely effective agent used [5, 6]. It is effective against all species of schistosomes that infect humans and is relatively cheap and easy to use, but PZQ does not provide a cure, since young schistosomula are mostly resistant to its anthelmintic effects [7]. This drug provides some relief to treated patients. However, young parasites, due to their intrinsic resistance to PZQ, escape elimination during treatment, grow to maturation, and begin to release eggs [5]. This mechanism of resistance is worrying, because, under this ineffective pressure, drug resistance may also arise in humans, as in murine models [8, 9].

The* S. mansoni* genome is structured in 7 pairs of autosomes and one pair of sex chromosomes (female = ZW, male = ZZ). Chromosomes range in size from 18 to 73 MB and can be distinguished by size, shape, and the C-banding technique [10]. According to the latest annotation, the* S. mansoni* genome is still considered as a draft with 380 Mb and 885 scaffolds. Despite this, about 81% of the bases are organized in these chromosomes. More than 45% of the predicted genes were modified, and the total number was reduced from 11,807 to 10,852 [11].

This parasite has a complex life cycle that involves many larval stages, an intermediate snail, and a final mammalian host. It is believed that the difference and developmental complexity observed between the different evolutionary stages and environments depend on the regulation of gene expression [12]. Several molecules are responsible for gene expression regulation, especially long noncoding RNAs (lncRNAs). They are defined as transcripts longer than 200 nucleotides and do not encode proteins presenting several regulatory functions. They can interact with DNA, RNA molecules, and transcription factors, participating in various biological processes, mainly gene regulation [13].

While our knowledge of the mechanisms and scope of lncRNA-mediated regulation is growing, our understanding of how lncRNAs themselves are regulated is still quite limited [14]. Regulating lncRNA expression would be expected to be an important cellular consideration given that lncRNAs have been implicated in regulating a variety of processes in eukaryotes including imprinting, dosage compensation, cell cycle regulation, pluripotency, retrotransposon silencing, meiotic entry, and telomere length [15–18]. They can also play important roles in numerous disease and physiological metabolism processes, such as X-chromosome inactivation, embryonic development, and pluripotency maintenance [16, 19].

These findings deeply changed disease pathobiology comprehension and led to the emergence of new biological concepts about human diseases, including the parasitic disease. Several methodologies were created to characterize and identify this RNA subtype. Noteworthy,* S. mansoni* researchers used these techniques and described a great picture of these lncRNAs and their participation in the disease processes. To date two studies have been published predicting lncRNAs molecules in schistosomes [20, 21] and only one [21] describes possible lncRNAs' functions of 181 sequences from 7431 total predicted lncRNAs in 5 life cycle stages in this parasite, including canonically spliced putative lincRNA and spliced lncRNAs that are antisense to protein coding genes. These functions were predicted considering that lncRNAs may act by regulating their flanking protein coding gene neighbors in many processes to the rapid adaptation of the parasite to several environments.

In this study, we aimed to predict novel lncRNAs in* S. mansoni* via a computational pipeline and investigate features including possible functions of these sequences. Here we describe a complete new set of 170 lncRNAs in adult worms, from which we selected 15 for expression analysis in male, female, and also, for the first time, praziquantel-resistant worms which are known to have a differential expression profile in several important genes in relation to susceptible worms [8, 22, 23]. Our results show a differential expression profile of lncRNAs and reinforce the importance of this RNA subtype in schistosome biology.

## 2. Material and Methods

### 2.1. Ethics Statement

All experiments that involve animals were authorized by the Ethical Committee for Animal Care of the Federal University of Ouro Preto (CEUA-UFOP protocol 2011/55). These procedures were conducted in accordance with the accepted national and international regulations for laboratory animal use and care.

### 2.2. Parasites

The* S. mansoni* LE strain was maintained by routine passage through* Biomphalaria glabrata* snails and BALB/c mice. The infected snails were induced to shed cercariae under light exposure for 2 hours. Adult worm parasites were obtained by liver perfusion of mice after 50 days of infection. The* S. mansoni* LE praziquantel-resistant (LE-PZQ) strains were obtained following a described method for inducing resistance to PZQ using infected* B. glabrata* snails [24]. Infected snails were treated 3 times with 100 mg/kg PZQ for five consecutive days with a one-week interval between them. Then, after this treatment the cercariae both from treated snails (LE-PZQ strains) and from nontreated snails (LE strains susceptible) were used to infect two groups of mice. These mice were treated 45 days after infection with 200, 400, or 800 mg/kg PZQ with three PZQ treatments, each treatment administered on 5 consecutive days, with 1-week interval, for selection of less susceptible parasites to PZQ following the method developed by Couto et al. [24]. Then, the LE-PZQ adult worms were obtained by mice liver perfusion, washed in RPMI 1640 (Sigma Chemical Co.), quick-frozen in liquid nitrogen, and stored at -80°C until use.

### 2.3. Data Set Download

The latest annotation data for* S. mansoni* (Genome Assembly release v5.2) were downloaded from GeneDB database (http://www.genedb.org) [25]. In order to perform prediction analysis in this pipeline, a RNA-seq library from 7-week-old mixed sex adult worms was selected and downloaded from ArrayExpress database [11] in the FASTQ format (http://www.ebi.ac.uk/arrayexpress/) under accession number E-MTAB-451.

### 2.4. Initial Transcriptome Assembly for S. mansoni

*S. mansoni* genome FASTA file and the annotation data in the GTF format were downloaded from GeneDB. One paired RNA-seq library was downloaded in the FASTQ format provided by sequencing using the Illumina Genome Analyzer IIx platform [11]. The quality was then analyzed using FastQC 0.11.4 [26]. The main parameters analyzed were the quality of the scores on the bases (quality of the* Phred* set as greater than 20 where it considers 1 error per 100 bases), per sequence quality scores, per sequence GC content, and removal of possible adapters used in the sequencing. After that, these reads were trimmed using Trimmomatic [27], and the following parameters were used: HEADCROP:15, LEADING:20, TRAILING:20, SLIDINGWINDOW:5:20, and MINLEN:50. Next filtering the quality of the files in the FastQC and removing the adapters, all the reads were mapped using the* S. mansoni* reference genome with the STAR aligner [28]. In order to use this program, it was necessary to index the genome in the format compatible with STAR. Once the indexing was performed, the next step was to perform the mapping of the reads with the* S. mansoni* genome as reference using STAR. The output SAM files of STAR were then converted to its compressed BAM format, and then indexed and sorted with SAMtools for further analysis [29]. Subsequently, the BAM files were assembled by Cufflinks 2.2.1 [30] using* de novo* mode to assemble transcripts for 3* S. mansoni* stage samples. Finally, assembly of transcripts was performed via Cufflinks, and the final file was submitted to the following pipeline filters for prediction of lncRNAs in* S. mansoni*. These steps were written in and performed with the use of custom Perl and Python scripts.

### 2.5. Identification of Novel lncRNAs Candidates

Cuffcompare was the first step used to compare the results generated by the* ab initio *assembly with the known annotations present in GeneDB in GTF format. The consensuses of novel transcripts were then used for further analysis. As a result of Cuffcompare, all the assemblies that were detected as new transcripts were categorized into 12 different categories according to their location compared with the* S. mansoni* reference genes [30]. We kept the three following classes: unknown intergenic transcript (u), a transfrag falling entirely within a reference intron (i), and exonic overlap with reference on the opposite strand (x). This final file was submitted to the following filters written in the programming languages Python and Perl.

In the second step a filter was used to extract the sequences equal or greater than 200 nucleotides. The third step was done using CPAT (Coding Potential Assessment Tool), an alignment-free method to predict RNA coding potential using four sequence features [31]. Only the transcripts classified by CPAT as noncoding transcripts were added to a new FASTA file. For our proposed filter, we used a prebuilt logit fly model as the classifier with optimum cutoff (CP) 0.39 (CP >=0.39 indicates coding sequence; CP < 0.39 indicates noncoding sequence) [31]. The fourth step consisted of the extraction of transcripts that presented putative ORF (Open Reading Frame) smaller than 300 nt using OrfPredictor server [32]. Transcripts with protein coding potential generally have ORF greater than 300 nt in size, generating proteins greater than or equal to 100 aa (amino acids) [33, 34]. In the fifth step we used an FPKM cutoff based on the distribution of the lncRNA expression level. Only FPKM values ≥2 were included in the final analysis. The sixth step was performed manually using the NCBI database as a reference and lncRNAs sequences from other works [21]. At this step, we manually removed other RNA types, transposons, and predicted protein coding genes that have homology in other* Schistosoma* species. The seventh and final step was performed to remove all the known lncRNAs in* S. mansoni* using BLAST version 2.7.1 [35, 36].

### 2.6. lncRNAs Features

The novel adult lncRNAs predicted were characterized in terms of genomic localization, exon number, transcript length, and log_2_ FPKM using R and R Studio with ggplot2 packaged [37].

### 2.7. Gene Ontology Enrichment Analyses

The possible lncRNAs' functions were hypothesized with Gene Ontology (GO) enrichment analysis approach (http://geneontology.org/). In this analysis, all neighboring genes up to 100,000 bases upstream and downstream of the lncRNAs were selected and then their functions were evaluated [38]. In this way, lncRNAs may act by binding to these protein coding genes by regulating their translation. The list obtained with all GO terms was created and plotted with R version 3.4.2, and R Studio version 1.0.136. The analysis method was based on Fisher's exact test and the -log10 (*P* value) was used to denote the significance of the GO term enrichment.

### 2.8. LncRNAs Expression Analysis

Approximately 100 mg adult worms were used for total RNA extraction performed according to the manufacturer's specifications (SV Total RNA Isolation System). Expression levels of 15 lncRNAs were quantified by RT-qPCR with Applied Biosystems ABI 7300 by using SYBR-Green PCR Master Mix (Roche®). We designed specific primers for each lncRNA, endogenous control, and positive control using Gene Runner version 6.5.46 (Supplementary Table S1). For the investigated transcripts, three biological replicates were performed and normalized to the endogenous control with specific primers for* S. mansoni EIF4E* [39]. A long terminal repeat (LTR) retrotransposon with 58% cover and 95% identity with* S. mansoni* Saci-4 LTR retrotransposon was used as a comparison parameter because it is a transcript found expressed in all chromosomes. Expression levels were calculated according to the 2^−ΔCt^ method [40] using the Applied Biosystems 7300 software. All these experiments were performed following MIQE guidelines [41].

### 2.9. Statistical Analysis

Statistical analysis for RT-qPCR was performed using GraphPad Prism, version 7.0 (San Diego, CA, USA). One-way and two-way analysis of variance (ANOVA), following by Tukey multiple comparisons, were performed to investigate significant differential expression of transcripts throughout the sexes and treatments. In all cases, the differences were considered significant when* P* values were <0.05.

## 3. Results

### 3.1. Initially Assembled Transcripts

**Figure 1:**
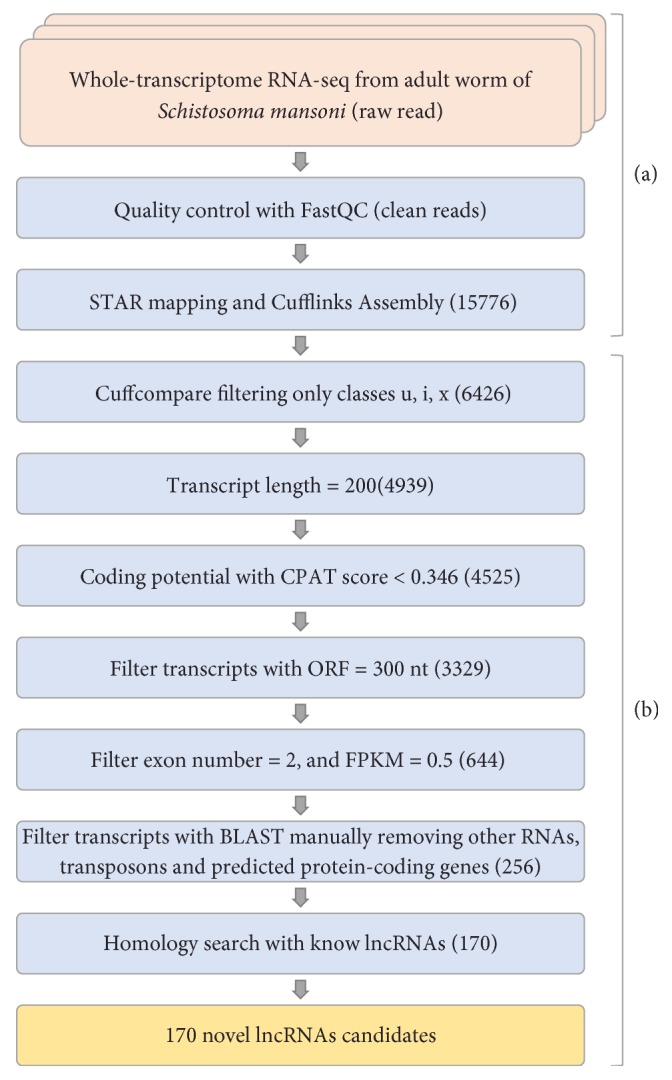
Integrative computational pipeline for the identification of lncRNAs in* S. mansoni*. (a) The raw RNA-seq data was preprocessed, aligned with STAR, and assembled using Cufflinks in ab initio mode. (b) The output was analyzed in several steps and algorithms were written in the Python and Perl programming languages. The numbers in parentheses represent the number of transcripts after each filtering step.

A stranded RNA-seq data set of the whole transcriptome was used to assemble transcripts (Figure 1(a)). We first trimmed more than 10 million raw reads (FASTQ reads) obtaining a total of 6 million clean reads. More than 5 million (82.18%) of them were mapped with STAR to the* S. mansoni* genome (v5.2) and calculation of summary statistics (Supplementary Table S2).* De novo* assembly from the aligned fragments was performed using Cufflinks with all the* ab initio* default parameters to generate 15,776 transcripts, which were then processed through the described pipeline.

### 3.2. Computational Pipeline to Predict Novel lncRNAs

Our computational pipeline applied multiple filters on these transcripts (Figure 1(b)) to predict novel lncRNAs in* S. mansoni* adult worm. First, Cuffcompare removed the assemblies overlapping with transcripts annotated in the reference genome. This includes reconstructed protein coding transcripts or annotated known noncoding transcripts. The selection of classes u, i, and x led to the removal of almost 60% of assembled transcripts. The remaining 6426 transcripts were submitted to a length filter (≥ 200) removing 1487. After passing this length filter, 4525 transcripts were classified by CPAT as noncoding transcripts following the ORF filter (≤ 300 nt) with 3329 transcripts remaining. For both categories, transcripts with exon number ≥ 2 and FPKM ≥ 0.5, only 644 transcripts were obtained. Since this study focuses on noncoding transcripts, a manual step was performed removing other RNAs, transposons, and predicted protein coding genes in other* Schistosoma* species. This filter led to 256 transcripts that were submitted to the last step removing all the known lncRNAs in* S. mansoni* by BLAST. From a total of 256 predicted lncRNAs, 170 (66.4%) were considered potentially novel lncRNAs candidates against 86 (33.6 %) that presented a homology (at least >70%) with other lncRNAs in* S. mansoni* (Supplementary Table S3).

### 3.3. lncRNAs Features

**Figure 2:**
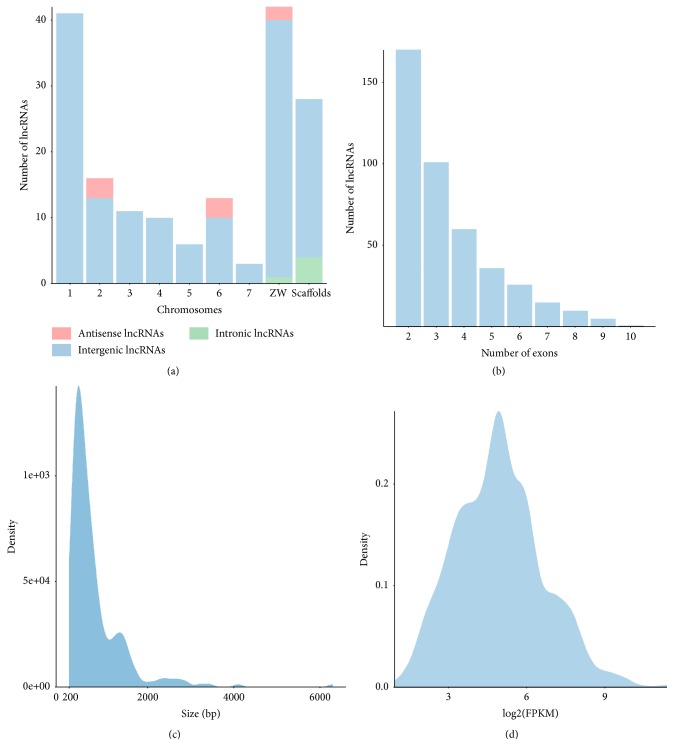
Features of* S. mansoni* adult lncRNAs. (a) Genomic localization of lncRNAs, (b) number of exons per transcripts, (c) transcripts length, (d) log_2_ FPKM expression.

To determine* S. mansoni* lncRNAs features, the genomic localization, exon number, transcript length, and log_2_ FPKM (Figure 2) were analyzed. In this data set we found that the majority of lncRNAs were found on chromosome 1, sexual ZW, and the scaffolds. The majority of the intronic lncRNAs were found on the sexual ZW and the unfinished scaffolds (Figure 2(a)). Moreover, lncRNAs had few exons per transcript (2-3) (Figure 2(b)) with most of them having transcript lengths between 200bp and 2000bp (Figure 2(c)). Finally, many of these lncRNAs could be confidently detected, with FPKM expression level between 2 and 9 (Figure 2(d)).

### 3.4. Overview of lncRNA RT-qPCR Validation.

**Figure 3:**
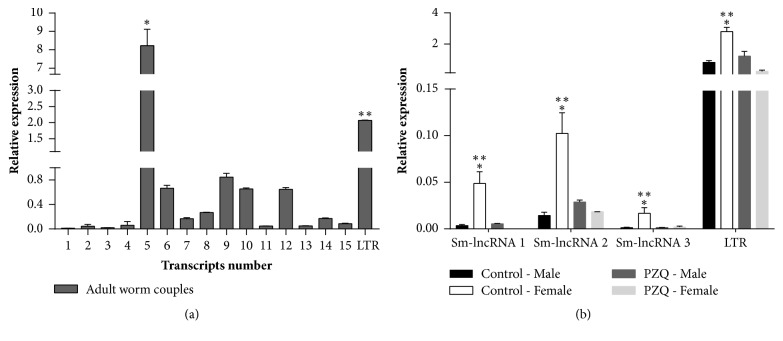
Relative expression of lncRNAs by RT-qPCR. (a) Fifteen Sm-lncRNAs and the LTR retrotransposon were selected for validation by RT-qPCR at the parasite adult stage (*∗*significantly different from all other lncRNAs and LTR, *∗∗*significantly different from all lncRNAs). (b) The first three lncRNAs and the LTR retrotransposon were selected to verify the expression between sexes into four groups Control-Male, Control-Female, PZQ-Male, and PZQ-Female (PZQ-Male and PZQ-Female are related to* S. mansoni* LE praziquantel-resistant strains) (*∗*significantly different from Control-Male, *∗∗* different from PZQ-Female). Two-way ANOVA and Tukey's posttest were used for calculating the statistical significance (*P* value* ≤* 0.05).

Based on nearby encoding genes, we selected 15 lncRNAs from a set of 170 putative molecules and the LTR retrotransposon to verify RT-qPCR expressions (Figure 3(a)). This selection was performed based on the enriched GO terms of the analyzed neighboring which were relevant genes for* S. mansoni* biology, for example, Smp_174670 (ubiquitin conjugating enzyme E2 J1), Smp_030780 (putative ubiquitination factor E4a), and Smp_199890 (venom allergen-like 7 protein).

Our relative expression results showed that* Sm-lncRNA 1*,* Sm-lncRNA 2, Sm-lncRNA 3, Sm-lncRNA 4, Sm-lncRNA 7, Sm-lncRNA 8, Sm-lncRNA 11, Sm-lncRNA 13, Sm-lncRNA 14, *and* Sm-lncRNA 15* had a low expression without statistical significance. Besides, 4 lncRNAs,* Sm-lncRNA 6, Sm-lncRNA 9, Sm-lncRNA 10, *and* Sm-lncRNA 12* had a higher expression than this lower group. The most highly expressed lncRNA was* Sm-lncRNA 5*. It was even more highly expressed than the LRT retrotransposon used as a comparison parameter.

We also selected the first three lncRNAs and the LTR retrotransposon to verify the expression between sexes into four groups: Control-Male, Control-Female, PZQ-Male, and PZQ-Female (PZQ-Male and PZQ-Female are related to* S. mansoni* LE praziquantel-resistant strains) (Figure 3(b)). All the 4 Control-Female expressions were higher than Control-Male and PZQ-Female.

### 3.5. Target Gene Prediction

**Figure 4:**
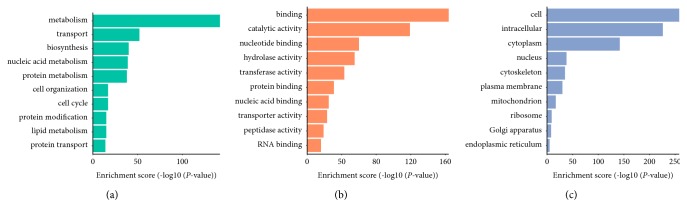
GO enrichment analysis of the lncRNA-target genes. The top 10 most enriched GO categories were calculated and plotted: (a) biological process; (b) molecular function; (c) cellular component.

GO enrichment analyses for the neighborhood target genes were made on three different aspects, namely, biological process (BP), molecular function (MF), and cellular component (CC). The 10 significantly overrepresented GO terms included 389 genes involved in BP, 555 genes involved in MF, and 768 genes involved in CC (Figure 4).

The largely enriched and meaningful BP terms were related to metabolism, transport, and biosynthesis. The enriched MF terms were predominantly related to binding including catalytic activity and nucleotide binding. As for CC, the most enriched terms were cell, intracellular, and cytoplasm. A detailed table of all 15 expressed lncRNAs was made including the neighboring coding genes and their respective GO entries (Supplementary Table S4).

## 4. Discussion

Several molecular and biochemical experiments revealed that lncRNAs may play diverse roles and functions in cellular biology. However, given the high abundance of lncRNAs and the poor-genetic conservation between species, the study of these molecules is extremely intriguing because they can be key regulators of species-specific biological processes. Notably, there are great prospects for a better understanding of* S. mansoni* biology, but much work will be needed to elucidate the specific role of lncRNAs in this parasite lifestyle. Thus, pipeline development for the* S. mansoni* genome is of extreme importance due to the lack of adapted and specific methodologies for the genus* Schistosoma*.

Current methodologies for predicting lncRNA genes are species-specific following the genome intrinsic characteristics of each species which increase data generation sensitivity and specificity. Each methodology used follows a flow chart that best adapts the steps and programs used [42–45]. This is a very important point because* S. mansoni* has particular characteristics since some of the repetitive fractions of DNA consist of tandemly repeated ribosomal genes of which there are 500-1000 copies per genome that represent 1.8-3.6% of the total DNA and nonribosomal repetitive sequences that comprise at least a further 2.0% of the total DNA [46].

In this pipeline, we introduced a manual step to remove other RNAs, transposons, and predicted protein coding genes in other* Schistosoma* species. In Figure 1, 39% of the initially putative lncRNAs showed significant homology with retrotransposons. This finding indicates an important difference of our pipeline. In addition, 33.6% of the putative lncRNAs identified were identical to those recently described for* S. mansoni* [21], reinforcing the hypothesis that the steps which were described in both studies are ideal for the identification of lncRNA in the genus* Schistosoma*.

In this work, we selected some lncRNAs to initiate expression studies, using lncRNA assumptions as criteria that are located in the proximity of genes involved with important pathways to* S. mansoni* survival, like the ubiquitin proteasome system, ribosomal protein (Supplementary Table S4), and others. In total, 100% of novel lncRNAs analyzed were expressed in adult worm couples (Figure 3). This is consistent with data from Vasconcelos et al. [21] who found similar percentages of expressed lncRNAs in* S. mansoni* adult worms.

The act of transcribing lncRNAs can have profound consequences on the ability of nearby genes to be expressed. For example, transcription of a lncRNA across the promoter region of a downstream protein coding gene directly interferes with transcription factor binding and thus prevents the protein coding gene from being expressed [47, 48]. We detected high levels of* Sm-lncRNA 5 *expression related to proteasome B1 subunit, responsible for peptidylglutamyl activity. Curiously, it is the lowest activity of the proteasome of* S mansoni *[49]. The proteasomes form a pivotal component for the ubiquitin proteasome system (UPS). It is implicated in protein ubiquitination, proteolysis, and degradation and was set as essential to* S. mansoni* biology [49]. Moreover, the* Sm-lncRNA 12* is differentially expressed in* S. mansoni *and occupies the neighborhood of E2 gene [50], leading us to hypothesize this participation on ubiquitin proteasome regulation. These findings reinforce themselves because differentially expressed lncRNAs, identified from human placentas, may regulate their associated mRNAs through several mechanisms and connect the UPS with infection-inflammation pathways [51].

Another set of putative lncRNAs, neighbor genes related to protein synthesis described in this work (Supplementary Table S4), are related to protein synthesis. Recently, lncRNAs have emerged as key players in the stress responses in plants [52] and eukaryotic cellular senescence [53]. The stress response is a feature of the* S. mansoni* lifestyle in both vertebrate and invertebrates hosts [54].

Furthermore, lncRNA levels dynamically change in response to various drugs. These alterations affect gene expression involved in several cells function such as cycle arrest, inhibition of apoptosis, and DNA damage repair [55].* S. mansoni* exposure to a drug, particularly PZQ, which is used in a single dose or repeatedly in reinfection, may induce drug resistance or reduced susceptibility over time [56]. The difference in gene expression between male and female in PZQ resistance worms suggest that lncRNAs can also be involved in drug resistance mechanisms in* S. mansoni.*

Several studies have provided insights into how genomic neighborhoods could influence gene expression levels, with important consequences for evolution, development, and disease [57].

## 5. Conclusions

In conclusion, we have identified a novel set of 170 lncRNAs* in S. mansoni* expressed in male, female, and PZQ resistant adult worms. These observations suggest that lncRNAs may be significant in parasite biology and be useful therapeutic targets. Further studies are required to dissect the function and mechanism of action of these RNA subtypes in normal biology and life cycle progression.

## Data Availability

The data used to support the findings of this study are available from the corresponding author upon request.

## Supplementary Materials

Supplementary Materials
* Supplementary Table S1:* primers pairs sequences selected for RT-qPCR experiments.* Supplementary Table S2:* detailed data obtained and mapping conditions for adult worm sample in RNA-seq.* Supplementary Table S3:* set of 170 putative lncRNAs identified by our computational pipeline.* Supplementary Table S4:* set of 15 expressed lncRNAs with the neighboring coding genes and their respective GO entries.

## References

[1] Steinmann P., Keiser J., Bos R., Tanner M., Utzinger J. (2006). Schistosomiasis and water resources development: systematic review, meta-analysis, and estimates of people at risk. *The Lancet Infectious Diseases*.

[2] Thétiot-Laurent S. A.-L., Boissier J., Robert A., Meunier B. (2013). Schistosomiasis chemotherapy. *Angewandte Chemie International Edition*.

[3] Gray D. J., Ross A. G., Li Y.-S., McManus D. P. (2011). Diagnosis and management of schistosomiasis. *BMJ*.

[4] WHO Schistosomiasis. Fact sheet No. 115, January 2012. http://www.who.int/mediacentre/factsheets/fs115/en/index.html.

[5] You H., McManus D. P., Gobert G. N. (2015). Current and prospective chemotherapy options for schistosomiasis. *Expert Opinion on Orphan Drugs*.

[6] Doenhoff M. J., Cioli D., Utzinger J. (2008). Praziquantel: mechanisms of action, resistance and new derivatives for schistosomiasis. *Current Opinion in Infectious Diseases*.

[7] Hagan P., Appleton C. C., Coles G. C., Kusel J. R., Tchuem-Tchuenté L.-A. (2004). Schistosomiasis control: keep taking the tablets. *Trends in Parasitology*.

[8] Aragon A. D., Imani R. A., Blackburn V. R. (2009). Towards an understanding of the mechanism of action of praziquantel. *Molecular and Biochemical Parasitology*.

[9] Pica-Mattoccia L., Cioli D. (2004). Sex- and stage-related sensitivity of *Schistosoma mansoni* to in vivo and in vitro praziquantel treatment. *International Journal for Parasitology*.

[10] Grossman A. I., Short R. B., Cain G. D. (1981). Karyotype evolution and sex chromosome differentiation in schistosomes (Trematoda, Schistosomatidae). *Chromosoma*.

[11] Protasio A. V., Tsai I. J., Babbage A. (2012). A systematically improved high quality genome and transcriptome of the human blood fluke *Schistosoma mansoni*. *PLOS Neglected Tropical Diseases*.

[12] Han Z.-G., Brindley P. J., Wang S.-Y., Zhu C. (2009). Schistosoma genomics: new perspectives on schistosome biology and host-parasite interaction. *Annual Review of Genomics and Human Genetics*.

[13] Ponting C. P., Oliver P. L., Reik W. (2009). Evolution and functions of long noncoding RNAs. *Cell*.

[14] Leone S., Santoro R. (2016). Challenges in the analysis of long noncoding RNA functionality. *FEBS Letters*.

[15] Cao J. (2014). The functional role of long non-coding RNAs and epigenetics. *Biological Procedures Online*.

[16] Li R., Zhu H., Luo Y. (2016). Understanding the functions of long non-coding RNAs through their higher-order structures. *International Journal of Molecular Sciences*.

[17] Fang Y., Fullwood M. J. (2016). Roles, functions, and mechanisms of long non-coding RNAs in cancer. *Genomics, Proteomics & Bioinformatics*.

[18] Grammatikakis I., Panda A. C., Abdelmohsen K., Gorospe M. (2014). Long noncoding RNAs (lncRNAs) and the molecular hallmarks of aging. *AGING*.

[19] Chen L.-L., Carmichael G. G. (2010). Decoding the function of nuclear long non-coding RNAs. *Current Opinion in Cell Biology*.

[20] Copeland C. C., Marz M., Rose D. (2009). Homology-based annotation of non-coding RNAs in the genomes of Schistosoma mansoni and Schistosoma japonicum. *BMC Genomics*.

[21] Vasconcelos E. J., daSilva L. F., Pires D. S. (2017). The Schistosoma mansoni genome encodes thousands of long non-coding RNAs predicted to be functional at different parasite life-cycle stages. *Scientific Reports*.

[22] Hines-Kay J., Cupit P. M., Sanchez M. C., Rosenberg G. H., Hanelt B., Cunningham C. (2012). Transcriptional analysis of Schistosoma mansoni treated with praziquantel in vitro. *Molecular and Biochemical Parasitology*.

[23] Sanchez M. C., Krasnec K. V., Parra A. S. (2017). Effect of praziquantel on the differential expression of mouse hepatic genes and parasite ATP binding cassette transporter gene family members during Schistosoma mansoni infection. *PLOS Neglected Tropical Diseases*.

[24] Couto F. F. B., Coelho P. M. Z., Araújo N. (2011). Schistosoma mansoni: a method for inducing resistance to praziquantel using infected Biomphalaria glabrata snails. *Memórias do Instituto Oswaldo Cruz*.

[25] Berriman M., Haas B. J., Loverde P. T. (2009). The genome of the blood fluke Schistosoma mansoni. *Nature*.

[26] Andrews S. FastQC: a quality control tool for high throughput sequence data. http://www.bioinformatics.babraham.ac.uk/projects/fastqc.

[27] Bolger A. M., Lohse M., Usadel B. (2014). Trimmomatic: a flexible trimmer for Illumina sequence data. *Bioinformatics*.

[28] Dobin A., Davis C. A., Schlesinger F. (2013). STAR: ultrafast universal RNA-seq aligner. *Bioinformatics*.

[29] Li H., Handsaker B., Wysoker A. (2009). The sequence alignment/map format and SAMtools. *Bioinformatics*.

[30] Trapnell C., Roberts A., Goff L. (2012). Differential gene and transcript expression analysis of RNA-seq experiments with TopHat and Cufflinks. *Nature Protocols*.

[31] Wang L., Park H. J., Dasari S. (2013). Coding-potential assessment tool using an alignment-free logistic regression model. *Nucleic Acids Research*.

[32] Min X. J., Butler G., Storms R., Tsang A. (2005). OrfPredictor: predicting protein-coding regions in EST-derived sequences. *Nucleic Acids Research*.

[33] Clamp M., Fry B., Kamal M. (2007). Distinguishing protein-coding and noncoding genes in the human genome. *Proceedings of the National Acadamy of Sciences of the United States of America*.

[34] Dinger M. E., Pang K. C., Mercer T. R., Mattick J. S. (2008). Differentiating protein-coding and noncoding RNA: challenges and ambiguities. *PLoS Computational Biology*.

[35] Camacho C., Coulouris G., Avagyan V. (2009). BLAST+: architecture and applications. *BMC Bioinformatics*.

[36] Altschul S. F., Madden T. L., Schäffer A. A. (1997). Gapped BLAST and PSI-BLAST: a new generation of protein database search programs. *Nucleic Acids Research*.

[37] Wickham H. (2009). *ggplot2: elegant graphics for data analysis*.

[38] Ørom U. A., Derrien T., Beringer M. (2010). Long noncoding RNAs with enhancer-like function in human cells. *Cell*.

[39] Liu S., Cai P., Hou N. (2012). Genome-wide identification and characterization of a panel of house-keeping genes in Schistosoma japonicum. *Molecular and Biochemical Parasitology*.

[40] Livak K. J., Schmittgen T. D. (2001). Analysis of relative gene expression data using real-time quantitative PCR and the 2^−ΔΔ*C*_*T*_^ method. *Methods*.

[41] Bustin S. A., Benes V., Garson J. A. (2009). The MIQE guidelines: minimum information for publication of quantitative real-time PCR experiments. *Clinical Chemistry*.

[42] Zhao Y., Luo H., Chen X., Xiao Y., Chen R. (2014). Computational methods to predict long noncoding RNA functions based on co-expression network. *Methods in Molecular Biology*.

[43] Ilott N. E., Ponting C. P. (2013). Predicting long non-coding RNAs using RNA sequencing. *Methods*.

[44] Zhang Y, Huang H., Zhang D. (2017). A review on recent computational methods for predicting noncoding RNAs. *BioMed Research International*.

[45] Luo H., Bu D., Sun L., Fang S., Liu Z., Zhao Y. (2017). Identification and function annotation of long intervening noncoding RNAs. *Briefings in Bioinformatics*.

[46] Simpson A. J. G., Sher A., McCutchan T. F. (1982). The genome of Schistosoma mansoni: Isolation of DNA, its size, bases and repetitive sequences. *Molecular and Biochemical Parasitology*.

[47] Martens J. A., Laprade L., Winston F. (2004). Intergenic transcription is required to repress the Saccharomyces cerevisiae SER3 gene. *Nature*.

[48] Katayama S., Tomaru Y., Kasukawa T. (2005). Antisense transcription in the mammalian transcriptome. *Science*.

[49] Guerra-Sá R., Castro-Borges W., Evangelista E. A., Kettelhut I. C., Rodrigues V. (2005). Schistosoma mansoni: functional proteasomes are required for development in the vertebrate host. *Experimental Parasitology emphasizes*.

[50] Costa M. P., Oliveira V. F., Pereira R. V. (2015). In silico analysis and developmental expression of ubiquitin-conjugating enzymes in Schistosoma mansoni. *Parasitology Research*.

[51] Luo X., Pan J., Wang L. (2015). Epigenetic regulation of lncRNA connects ubiquitin-proteasome system with infection-inflammation in preterm births and preterm premature rupture of membranes. *BMC Pregnancy and Childbirth*.

[52] Wang J., Meng X., Dobrovolskaya O. B., Orlov Y. L., Chen M. (2017). Non-coding RNAs and their roles in stress response in plants. *Genomics, Proteomics & Bioinformatics*.

[53] Kim C., Kang D., Lee E. K., Lee J.-S. (2017). Long noncoding RNAs and RNA-binding proteins in oxidative stress, cellular senescence, and age-related diseases. *Oxidative Medicine and Cellular Longevity*.

[54] Knight M., Ittiprasert W., Arican-Goktas H. D., Bridger J. M. (2016). Epigenetic modulation, stress and plasticity in susceptibility of the snail host, Biomphalaria glabrata, to Schistosoma mansoni infection. *International Journal for Parasitology*.

[55] Lipovich L., Johnson R., Lin C.-Y. (2010). MacroRNA underdogs in a microRNA world: evolutionary, regulatory, and biomedical significance of mammalian long non-protein-coding RNA. *Biochimica et Biophysica Acta (BBA)—Gene Regulatory Mechanisms*.

[56] Pinto-Almeida A., Mendes T., de Oliveira R. N. (2016). Morphological Characteristics of Schistosoma mansoni PZQ-Resistant and -Susceptible Strains Are Different in Presence of Praziquantel. *Frontiers in Microbiology*.

[57] Sanjana N. E., Wright J., Zheng K. (2016). High-resolution interrogation of functional elements in the noncoding genome. *Science*.

