# Supplementary material for: Identification of 170 New Long Noncoding RNAs in Schistosoma mansoni
Source: Biomed Res Int. 2018 Jul 11;2018:1264697. doi: 10.1155/2018/1264697 (PMC6077669; doi:10.1155/2018/1264697)
Supplement: Supplementary Materials — Supplementary Table S1: primers pairs sequences selected for RT-qPCR experiments. Supplementary Table S2: detailed data obtained and mapping conditions for adult worm sample in RNA-seq. Supplementary Table S3: set of 170 putative lncRNAs identified by our computational pipeline. Supplementary Table S4: set of 15 expressed lncRNAs with the neighboring coding genes and their respective GO entries. [file 1264697.f1.docx]

| Primer name | Transcript ID | Forward Primer (5′-3′) | Reverse Primer (5′-3′) | PCR product length (bp) |
| --- | --- | --- | --- | --- |
| Sm-lncRNA 1 | TCONS_00001011 | AAGGGATGAGTTGACTGC | ACACGAAGACACCTATGACC | 119 |
| Sm-lncRNA 2 | TCONS_00012347 | AGACAATGCGATGCCGTTAG | TTTGGAACTCGTCAGCTAGG | 97 |
| Sm-lncRNA 3 | TCONS_00013257 | TCACATCTCGCAACTCAG | TCACATCTCGCAACTCAG | 103 |
| Sm-lncRNA 4 | TCONS_00003004 | TTTCGACACGGCAACTGATC | GCCGATTCAGTGTAGCAAAG | 99 |
| Sm-lncRNA 5 | TCONS_00000625 | GATCGAGCTGTAACTGCAC | GATCCACATCCATATGAGTG | 92 |
| Sm-lncRNA 6 | TCONS_00001840 | GACTGTTGGAAGAGGAAATG | GAGGATTTAAGCGACCATTG | 82 |
| Sm-lncRNA 7 | TCONS_00009100 | CCGATGAGATGCGTATAG | GCAACACAGTGAGGTAGAG | 134 |
| Sm-lncRNA 8 | TCONS_00009852 | CCACACAGGTAGTTCAGC | GAATCACTTGCACTTCGC | 111 |
| Sm-lncRNA 9 | TCONS_00009849 | CTGTGAGAATGGTGGATG | ACGTTTATGAGCCGTAGC | 83 |
| Sm-lncRNA 10 | TCONS_00009851 | GTGATATGCCCGGACAAAG | TTGAACGAGCAGCTGGAC | 108 |
| Sm-lncRNA 11 | TCONS_00012478 | CCTCGTGTTTGTGCTTTG | GGAATGTGATTGCCTAGTCG | 82 |
| Sm-lncRNA 12 | TCONS_00010393 | GCACTTGACACTAACCAGG | GGAGCTGTTCACTCATTG | 125 |
| Sm-lncRNA 13 | TCONS_00010903 | TTCCCTCCAGACTATGATCC | CACGTATTGCACCTGATG | 144 |
| Sm-lncRNA 14 | TCONS_00011021 | GTTGAAGAAGGTGAGTGC | GTGGAGGACTTGGAGATAC | 124 |
| Sm-lncRNA 15 | TCONS_00013835 | CCATGCAAGTGTGATCCG | GTGGGATTATCAGCTGCAGG | 145 |
| LTR-Retrotransposon | Saci-4 LTR | GGGTGCATCAGAGTAATC | ACTTGATCCGCATACTCC | 124 |
| SmEIF4E | Smp_001500 | TGTTCCAACCACGGTCTCG | TCGCCTTCCAATGCTTAGG | 89 |

**Supplementary Table S1:** Primers pairs sequences selected for RT-qPCR experiments.

**Supplementary Table S2**. Detailed data obtained and mapping conditions for adult worm sample in RNA-Seq.

| Sample name | Adult Worm ERR022873 |
| --- | --- |
| Raw reads | 10521255 |
| Clean reads | 6371002 |
| Total mapped | 5235888 (82.18%) |
| Unique mapped | 4721748 (74.11%) |
| Multiple mapped | 514132 (8.07%) |
| Number of splices | 1226166 |

**Supplementary Table S3:** Set of 170 putative lncRNAs identified by our computational pipeline.

|  |
| --- |
| >TCONS_00000002 gene=XLOC_000001 |
| cgttgttcctgtatcccgcttcaatttgcaccatccgacaaactctgtggtcactattac |
| taagtcgggcctacagatcatctgccgtaaacatgggagttcatcagttgagcaaggtaa |
| ttggtgataatgcacagaaggcagtcaaaagctgtgaaataaaatcatatttcggaagga |
| aagtagcaattgacgcttccatgtcaatttaccagtttcttattgctgttagacaagaag |
| ggaatactctaatgaatgcagaaggagagtcgactagtcatttgatgggcatgttttata |
| gaacaataagaatgatagaaaatggaattaaacctgtatatgtttttgaagggaaaccac |
| >TCONS_00000006 gene=XLOC_000004 |
| aggagcgtataaggaatggagcgaaaaagttattaaaagccaagaacacaactactcaag |
| gtagaatagacaactttttcacctgtgttccatcaaaaagtgattcatccatgtcaactc |
| ccaacagcaataacaaaaaggtatccaacagtgcatccgatcgtaaacgtaaaagtaaca |
| ctggcgcatcgaacggatacaaaaggccgaaataaatcaaatatccccacaattcccccg |
| agactgtgtccggtgaattgttaccacttgtcagtttcatgtatccttcacttgtgtatg |
| taatttttcattatagctcatcagatctttagaataaat |
| >TCONS_00001468 gene=XLOC_000739 |
| taaagtcacctttcatctcacatctccgggttttatacttttcagctacattaatgtatt |
| tgacgtcaaacagctaagaagaaaactatcggagttctatgacccttctgcatacctgac |
| attccagatagccccttacgtctgtcagtcttcattgaatgaatgttccgacaaaagtta |
| cagaccaaagtatcagcccctttgggccacctatgaacgtttcgctgttaacagtttgcc |
| tggtgggttccctctaccatcatgggcctggttcgctgttaaagaggagtccatgcctcg |
| gagattaagaggacccagcttatgtaaacaggataggaaactcctgaagtcattgaaagg |
| atatcaaatgggacacatacagagattggtatcagttacacgctcagcttctagcgcttc |
| cgaccgctcaataacgaacattccacccccaccgatagcgcgaatggagtgtcgtggaag |
| tgatcaggttctaggtttgctttgccgtttgcccgatctcgtgcatgtaagaagtgacag |
| gtactcacatttggatgtaatttggtccgaattacccttggtttgcatgcgatactcatg |
| tattgcagcagaatcagtgccacaggatacacgccccagcgacttagaaggcatttcaga |
| tcaactgctaaacattgctccttatggtaactatgccgtccgcttggtttgtcgtatgct |
| catattgcatcaaagtggtaaaattcttcaggaagactggtgcgttactccattgccaag |
| ttcatatttgcagtgcaacatcgttataaaaatgagcatatcagtgtagcaccgttacca |
| tgtgggttagaaacatgcttcactttattgtgggtaattgggtaatatttgttgtattca |
| cataaatgtatctcaaagttgtggtacacaagcataaacctacttagttacggagtgttc |
| actgattatcacagaggctgtgtcaaattttatgacttcaagaacaccgtctacgctagc |
| atcaactctcaaaccaggtggtatgccagacgactctgtttgttcaaaattagttgaaga |
| gctgtccattgcaactggaatgaataaggcttattctttacaatgtttaactgagtgccg |
| gtttgatttagactctgcattacactcatttcaaaaagtacatgaagccggactattgcc |
| cgctgaagcattctcactaatttaactattcatattgttttagtgtcgtaat |
| >TCONS_00001469 gene=XLOC_000739 |
| agcacgtgatccatgaagaaacgacgtgggttcaaaaagaaggatgagttatttgccgaa |
| gatgagggcgaatgcaatctggttaatgccagcatactgcgagtggagcacgttagttca |
| aaaactaagcggcgcaatcgaaaaaagacactaaaaaaacttgaaaacatccagttcctc |
| aatgtctgtgacaaatatttagcttcaaaatcccgtgtgcttcctaaggtcctcccagaa |
| attttgcctcaattctgtagcaatcctgaaatattaaacttcctaagtgcttttgtgaac |
| aactacattaatgtatttgacgtcaaacagctaagaagaaaactatcggagttctatgac |
| ccttctgcatacctgacattccagatagccccttacgtctgtcagtcttcattgaatgaa |
| tgttccgacaaaagttacagaccaaagtatcagcccctttgggccacctatgaacgtttc |
| gctgttaacagtttgcctggtgggttccctctaccatcatgggcctggttcgctgttaaa |
| gaggagtccatgcctcggagattaagaggacccagcttatgtaaacaggataggaaactc |
| ctgaagtcattgaaaggatatcaaatgggacacatacagagattggtatcagttacacgc |
| tcagcttctagcgcttccgaccgctcaataacgaacattccacccccaccgatagcgcga |
| atggagtgtcgtggaagtgatcaggttctaggtttgctttgccgtttgcccgatctcgtg |
| catgtaagaagtgacaggtactcacatttggatgtaatttggtccgaattacccttggtt |
| tgcatgcgatactcatgtattgcagcagaatcagtgccacaggatacacgccccagcgac |
| ttagaaggcatttcagatcaactgctaaacattgctccttatggtaactatgccgtccgc |
| ttggtttgtcgtatgctcatattgcatcaaagtggtaaaattcttcaggaagactggtgc |
| gttactccattgccaagttcatatttgcagtgcaacatcgttataaaaatgagcatatca |
| gtgtagcaccgttaccatgtgggttagaaacatgcttcactttattgtgggtaattgggt |
| aatatttgttgtattcacataaatgtatctcaaagttgtggtacacaagcataaacctac |
| ttagttacggagtgttcactgattatcacagaggctgtgtcaaattttatgacttcaaga |
| acaccgtctacgctagcatcaactctcaaaccaggtggtatgccagacgactctgtttgt |
| tcaaaattagttgaagagctgtccattgcaactggaatgaataaggcttattctttacaa |
| tgtttaactgagtgccggtttgatttagactctgcattacactcatttcaaaaagtacat |
| gaagccggactattgcccgctgaagcattctcactaatttaactattcatattgttttag |
| tgtcgtaat |
| >TCONS_00001470 gene=XLOC_000739 |
| ttgagcacgtgatccatgaagaaacgacgtgggttcaaaaagaagggtagagtatagtca |
| tcaccttcatttataaagagttcacagatgagttatttgccgaagatgagggcgaatgca |
| atctggttaatgccagcatactgcgagtggagcacgttagttcaaaaactaagcggcgca |
| atcgaaaaaagacactaaaaaaacttgaaaacatccagttcctcaatgtctgtgacaaat |
| atttagcttcaaaatcccgtgtgcttcctaaggtcctcccagaaattttgcctcaattct |
| gtagcaatcctgaaatattaaacttcctaagtgcttttgtgaacaactacattaatgtat |
| ttgacgtcaaacagctaagaagaaaactatcggagttctatgacccttctgcatacctga |
| cattccagatagccccttacgtctgtcagtcttcattgaatgaatgttccgacaaaagtt |
| acagaccaaagtatcagcccctttgggccacctatgaacgtttcgctgttaacagtttgc |
| ctggtgggttccctctaccatcatgggcctggttcgctgttaaagaggagtccatgcctc |
| ggagattaagaggacccagcttatgtaaacaggataggaaactcctgaagtcattgaaag |
| gatatcaaatgggacacatacagagattggtatcagttacacgctcagcttctagcgctt |
| ccgaccgctcaataacgaacattccacccccaccgatagcgcgaatggagtgtcgtggaa |
| gtgatcaggttctaggtttgctttgccgtttgcccgatctcgtgcatgtaagaagtgaca |
| ggtactcacatttggatgtaatttggtccgaattacccttggtttgcatgcgatactcat |
| gtattgcagcagaatcagtgccacaggatacacgccccagcgacttagaaggcatttcag |
| atcaactgctaaacattgctccttatggtaactatgccgtccgcttggtttgtcgtatgc |
| tcatattgcatcaaagtggtaaaattcttcaggaagactggtgcgttactccattgccaa |
| gttcatatttgcaggagtgttcactgattatcacagaggctgtgtcaaattttatgactt |
| caagaacaccgtctacgctagcatcaactctcaaaccaggtggtatgccagacgactctg |
| tttgttcaaaattagttgaagagctgtccattgcaactggaatgaataaggcttattctt |
| tacaatgtttaactgagtgccggtttgatttagactctgcattacactcatttcaaaaag |
| tacatgaagccggactattgcccgctgaagcattctcactaatttaactattcatattgt |
| tttagtgtcgtaat |
| >TCONS_00001471 gene=XLOC_000739 |
| ttaacattgagcacgtgatccatgaagaaacgacgtgggttcaaaaagaaggagttcaca |
| gatgagttatttgccgaagatgagggcgaatgcaatctggttaatgccagcatactgcga |
| gtggagcacgttagttcaaaaactaagcggcgcaatcgaaaaaagacactaaaaaaactt |
| gaaaacatccagttcctcaatgtctgtgacaaatatttagcttcaaaatcccgtgtgctt |
| cctaaggtcctcccagaaattttgcctcaattctgtagcaatcctgaaatattaaacttc |
| ctaagtgcttttgtgaacaactacattaatgtatttgacgtcaaacagctaagaagaaaa |
| ctatcggagttctatgacccttctgcatacctgacattccagatagccccttacgtctgt |
| cagtcttcattgaatgaatgttccgacaaaagttacagaccaaagtatcagcccctttgg |
| gccacctatgaacgtttcgctgttaacagtttgcctggtgggttccctctaccatcatgg |
| gcctggttcgctgttaaagaggagtccatgcctcggagattaagaggacccagcttatgt |
| aaacaggataggaaactcctgaagtcattgaaaggatatcaaatgggacacatacagaga |
| ttggtatcagttacacgctcagcttctagcgcttccgaccgctcaataacgaacattcca |
| cccccaccgatagcgcgaatggagtgtcgtggaagtgatcaggttctaggtttgctttgc |
| cgtttgcccgatctcgtgcatgtaagaagtgacaggtactcacatttggatgtaatttgg |
| tccgaattacccttggtttgcatgcgatactcatgtattgcagcagaatcagtgccacag |
| gatacacgccccagcgacttagaaggcatttcagatcaactgctaaacattgctccttat |
| ggtaactatgccgtccgcttggtttgtcgtatgctcatattgcatcaaagtggtaaaatt |
| cttcaggaagactggtgcgttactccattgccaagttcatatttgcaggagtgttcactg |
| attatcacagaggctgtgtcaaattttatgacttcaagaacaccgtctacgctagcatca |
| actctcaaaccaggtggtatgccagacgactctgtttgttcaaaattagttgaagagctg |
| tccattgcaactggaatgaataaggcttattctttacaatgtttaactgagtgccggttt |
| gatttagactctgcattacactcatttcaaaaagtacatgaagccggactattgcccgct |
| gaagcattctcactaatttaactattcatattgttttagtgtcgtaat |
| >TCONS_00001589 gene=XLOC_000805 |
| gtaacaatcaccattttttcttttataggtatactaataatgacattgttctacttattt |
| gacagtcatatgatttatgtctaatttcaactaataagtatgggaggatttgtggagatt |
| gtagaatattcatcgcttaagacaagaactgatctcagctaaactaccattcaaaacctg |
| gaaacactggacggccctctcttcctagtattggattcatcatcaattcacatcttcgac |
| tcgaatctaaaaccttctgtctcgtgtgccaatgcttaacctaatgactactgaatcgac |
| atccaacggtgtacatgtctagcttcaatcaactcaacatgttccacaaccatcttacac |
| acgacatggttgaactccactagccacagcttctcactattactccgagaactccattct |
| aaagcttgtcacttgtgagtatataatggttattagtataaagtaaatttgtgtgaatta |
| ataaacactttagaactttaatgaagtcacacatacattatttctccccatattttaaac |
| acttttcccacccacattgattttattaagagaatataagttttgtttgtgtttaacaaa |
| caattcaattcacccataacatagcaaaatcattacaacactttccactattgagaagag |
| aagttcaattttgaaatgcaagagaacaaaatcacctcaacacagagcataatttatgac |
| gtaatcaaccataaaattcccaattcctttttcatgatgttgattttctctgttgtacac |
| ctttatttatccaaatgtgatattgtgtcaatggatctttattttctcacaaacttta |
| >TCONS_00001708 gene=XLOC_000850 |
| tttgttcactcaatctgagcttattataattgatgatgatgatgaagatgataatgataa |
| tgatgtgtaaatctattcattgtgttatattatattttgttattggtttaataacaatta |
| actgtgcagaatataatgaaggattattatataactccaggtcaaatatattgagtaaac |
| gttggtatcctgtgaaagaatttcattatgatgaaccgttagagattaaaaaaagaccct |
| taatgttccataagcgttggtttccagtgaaagaattccattatgatggaccacttgaag |
| tgaaaaaacgacctaaattctacgataaacgttggtctcctgtcaaagaatttcattatg |
| atgaaccaatagaagtgagaa |
| >TCONS_00000286 gene=XLOC_000138 |
| tttatacgcagtgctgttcaaaagaacatctactttactacgcatcaagtttgtgaagga |
| gaaaatagaatcaatcttcatctatagaatagaaggtgaaaagtggaatgacatacatta |
| tgaggaatatattgatcaaaatacttcaattatttgtaattatagaaattttcattcatt |
| taaatatatatcaaacagcagtacacactgctccaatagaaacatatacaaatgatgatg |
| tgaaattttcaaaaccatgtcctgaagaaaactacagatatcacaatgctactggatatt |
| ttatgtgcattgttgaaacagctgagaaatgcattgacttgtgtcaacaaataggatgca |
| atgatttatattttatgagtgtgatcccttcgaaaaatgataaaatcaaacgaaactatc |
| ggtgtcgttgtttccaggattatcacgtctgcttttataatccattgccaagatatcata |
| acattaattagttgataataataataatggtattagtaatcgctaccattaatattatta |
| ctgttatcaaaaaaacatgtgttttcctctctaaattggtttgtattcgtcgataattct |
| aagaaaaatagtgtaatgtgtatgtgtagaaactgctttcaaactaaatttggatattat |
| ttcattgggattcaacgtatataaaggaaacaaaaatctgtacatttgaatggttgcatc |
| aaatagtgttctatatatttgactgtgtgtgcattttttgtgtaccacttgtttgtttta |
| aaagacaattatatgtttaaccatcccctccccccattttaactgaaaaactcaactggg |
| agattattatctgttttttattgtaataatatatataacgatgtataaaactaagtaagc |
| cacttacataatcgacactgagattaattttttctaaaaataaattacataacatagcat |
| atgtatgctgctagtaaagaataatgcatgtatgtatccttaatgttgtaagagcttgag |
| atttataaatccctatttttctgttttttctgtttgtaacgaaccattattttgtttgtt |
| attggcaacttcatgttgtacactatttgcaaattaagaaaatgtttcggtcctaagtat |
| aaaatttaaatcaggaaaaacgataaaatggtgctatgtgcgatattattgtgaagtttt |
| tccttatttccttctcttcccctttctcattgtaactgttcctaaagtgttgagagcaca |
| ccattcaacattgaatatctcagcaaattaaaacaatgaatggcaacagtataatatatt |
| catatggtggattg |
| >TCONS_00001725 gene=XLOC_000858 |
| aacctactgacaaggaaatttagaagcaaaaccgtacttggtggggacggacaatgggaa |
| tacgaagttggagaaccactgaggagggaaaaccagttttcagagcatctaatggagtca |
| ccacaagctccactgtttattcgtagcgatgttccacgtgcattccaatggcgtatccgt |
| aacttaccttacccattagatgtgtacagagtttccgttgaagatgggaata |
| >TCONS_00000378 gene=XLOC_000186 |
| cgccattaaatctattttgcgctagtccaaagatggcctgtcctttagtgataaacaaga |
| actcagttaaagctttggcagcatggtggctttctaacgagtcatctttgaattatgtag |
| ggcttacaccaaaaagcggacgtgggaaagtgtacattgatatgcacagtccagggatca |
| ttgttgggatgccgttcgtcgatgccattctaaaagaaagcagttgcgaaatagagtggc |
| atgtatgcgagggtcagacagtaaaatcctgtcctgtaagagttgctacgatcagtgggg |
| ctgatgaagatattttcttttgcgaaaacttggttatttcggtcctttcgagagccagtg |
| ggatagcaaccttggccagcagaatccaattaattatacaggaagtttcgtggaagggga |
| caatatatatgcctgatagacggaccccgggttttggtcttgttgaagagtatgcaatga |
| tgatatcaggagtttctgaacgtaaagcatcggttagcgttcgctgccaaaatatggatg |
| ctgagagcttaaaaactgccatagatgaagttcggtctagagttggttctattcccgttc |
| atgttgcctgctcaaggttagatgaagcctgtttagctgctggagctggagcagacatat |
| tacttactgggttgagtgctaaggagattttagatattgctactcaagtgaaagaatcat |
| tcccagaaattcaggttatcgctagcggcttttttgatgactcagacgtaaaactgtgcg |
| caactcgctatgtggatcacttaacatctctcaaactgtgcaacggttatgcttttgttg |
| atttccaaatggcgtatgttaaagacaaacttgttgaaaatgcagaaactacgaacttcg |
| tagttttactagaagaaaatgaatcgccctctcctccaaaatcaaaatcaccaggccctc |
| aacccactgaagaagtggctagctcaattcctgcgccattaaagaagccccgcttagtcg |
| atgatgagggcgacacccctaaatcagctaagctcaatggtactccaactcgcaatacca |
| atccgacggccccagatccgaattggtccacttttcccaatgcggctaatgcacagaatc |
| agaagcgtgctcagcgaattcttcgacatcctcagtacacaccaccacaaaggtcgaatg |
| ttcctcgctcacaaactcctggtggttttccgtttttcatgtcgaacccgcgacttatgc |
| ctccaacatctacgccaacacgggctccggctatgctcttacaaccgggcttaaatatta |
| acaatcaggtgtcaaatgtactgactcctcccagcaaccaacagaatatcagtatgcgtt |
| tacccatggtacctcctcaattgcaacaaccaccacagtcaatgccacaacgtatgccat |
| caaaccctccaggaatgctcaatacaaatcaacctatgggtggtgggtttttgaacaata |
| ttcaaccaccccataatcaaccaccgaactggcaaatgattccaggcggtcccggtgggc |
| atccaatgggcgggatcggcttaggaatgcgtgttggtggtcctggaggacaaaatgtga |
| ataattgccggtcatgtggttttccgaaccctccatcaatgcctttttgtcgaaactgtc |
| gttctggccttcggtgaatttaagggtcctttgtgttattggcgcttaatttactatgta |
| atctgtgtttacacatatttcttt |
| >TCONS_00000379 gene=XLOC_000186 |
| cgccattaaatctattttgcgctagtccaaagatggcctgtcctttagtgataaacaaga |
| actcagttaaagctttggcagcatggtggctttctaacgagtcatctttgaattatgtag |
| ggcttacaccaaaaagcggacgtgggaaagtgtacattgatatgcacagtccagggatca |
| ttgttgggatgccgttcgtcgatgccattctaaaagaaagcagttgcgaaatagagtggc |
| atgtatgcgagggtcagacagtaaaatcctgtcctgtaagagttgctacgatcagtgggg |
| ctgatgaagatattttcttttgcgaaaacttggttatttcggtcctttcgagagccagtg |
| ggatagcaaccttggccagcagaatccaattaattatacaggaagtttcgtggaagggga |
| caatatatatgcctgatagacggaccccgggttttggtcttgttgaagagtatgcaatga |
| tgatatcaggagtttctgaacgtaaagcatcggttagcgttcgctgccaaaatatggatg |
| ctgagagcttaaaaactgccatagatgaagttcggtctagagttggttctattcccgttc |
| atgttgcctgctcaaggttagatgaagcctgtttagctgctggagctggagcagacatat |
| tacttactgggttgagtgctaaggagattttagatattgctactcaagtgaaagaatcat |
| tcccagaaattcaggttatcgctagcggcttttttgatgactcagacgtaaaactgtgcg |
| caactcgctatgtggatcacttaacatctctcaaactgtgcaacggttatgcttttgttg |
| atttccaaatggcgtatgttaaagacaaacttgttgaaaatgcagaaactacgaacttcg |
| tagttttactagaagaaaatgaatcgccctccacaaaggtcgaatgttcctcgctcacaa |
| actcctggtggttttccgtttttcatgtcgaacccgcgacttatgcctccaacatctacg |
| ccaacacgggctccggctatgctcttacaaccgggcttaaatattaacaatcaggtgtca |
| aatgtactgactcctcccagcaaccaacagaatatcagtatgcgtttacccatggtacct |
| cctcaattgcaacaaccaccacagtcaatgccacaacgtatgccatcaaaccctccagga |
| atgctcaatacaaatcaacctatgggtggtgggtttttgaacaatattcaaccaccccat |
| aatcaaccaccgaactggcaaatgattccaggcggtcccggtgggcatccaatgggcggg |
| atcggcttaggaatgcgtgttggtggtcctggaggacaaaatgtgaataattgccggtca |
| tgtggttttccgaaccctccatcaatgcctttttgtcgaaactgtcgttctggccttcgg |
| tgaatttaagggtcctttgtgttattggcgcttaatttactatgtaatctgtgtttacac |
| atatttcttt |
| >TCONS_00000384 gene=XLOC_000189 |
| tactgattgtggattaatgtgtcataacgtagaagctaacaacatttagtgtgtagtgat |
| ggttaagtgatttagcacttatcttccaaccgtaagttcttaggtctgtatcccacttca |
| tctaccttaatttgaacagatgattagtatcaatagtcctcaaatcaaaataaattgaca |
| tatatttggtgagttattattttactcatggtcactgtaataattttagattgtaagttt |
| tggcaaaaaaatattccaatcggtctaaggatactcccatctttttaatatctagtaggc |
| ttaaggacatctgcaccacatttttacaatacaaactgcaagttttgggaggactttatt |
| tcttggatttatgattttaacaacggaatccgtcatagagtgggcaaactaaaatttaga |
| tcactggcgtcatagtaaagtgtaatggtctatccgcaataaatgtgccaatatattttt |
| cttggctttctacatgaggacgcaacgtagattatgtaatgtcccacagttattgcaagt |
| ttaatttatatttatgatttcaacatagtgtgataccaatgaattttctgaacgccgtcg |
| ttttacgctctgctttttaatattttcctcagatccccatagaatccaaactgttttcta |
| gtatgtacttatctgttcaaaagtgtgaccttgccgtcgctttgttcaaaaaattcactg |
| gtcttgtacagttcattgatgtacagaaagccagtcaagatgaaattaattggattaagt |
| acgttttttcaagattcagcctcattttgaaactgttggaattactgctgacaaaggtga |
| accatgggtcaagcgacttgtcaacattgaatgtcctctacgatcctctcactcataatg |
| atcccataagtgcttggtttgcttcaaaacattctgttaaactagttactcctccaagtc |
| catatgaagcgtatctgaccataggcaactgtcctagtactccaggagcaatttcccgtc |
| tattagcgttaacggcatggcctcaaatgtgtgtattcaacggcagtgcgaaacaagcat |
| tattgtttacgaacctttggagtgagcttgaatctgttcaaatagaactcgaacttttaa |
| acacttttaaagcaaacctacctaatttgcttgcggaccatctgttcacgaaaacgatca |
| atcatgcctcggctttccgtgatttatacacactcgtttgtggaaacagtccatcaattt |
| tgaaaagcaacgtataaataacattctacacatttccatatcttgtatatatcatatcta |
| ctatagaaacagatttgcaaacatgtacaaacctttttagctgttatgttcattcttt |
| >TCONS_00000385 gene=XLOC_000190 |
| ttttcaaaaaatattcgaagggacttatttactgtgccggaactgcactatgtgttttaa |
| ggtcatttgtcctaagtgtggaaagactacatggagtggatgtggaaatcatatagaaca |
| agttttgaaagatatacgcccagaagagcgatgtcaatgcccacgttcttaagtaactca |
| catgtttctacattgtacaacaattcagatagttgaggtgtatttttagtttcacactac |
| ttacaagctagcttatttctcttattgataacctgtttatttttggttttaatttgtaga |
| gctcaatacttttgcttgttgtattatactactgtggctttaagtttattat |
| >TCONS_00001840 gene=XLOC_000922 |
| accaaattgagcagaaacttaaatcattaaacccacttcatatcgaaatagttgactttt |
| cagatggatgcggtctgaaatttgatgtaaaagtcgtctcacaagaattcgagggtaaat |
| cgcttgttgacagacacagactgttggaagaggaaatgaaatcagtacatgcccttactc |
| taaaaacgctagctccttcacaatggtcgcttaaatcctcctaacacccttatcatcggg |
| ctcgataattggaaattgtcatatatttttgtaaataa |
| >TCONS_00001841 gene=XLOC_000922 |
| accaaattgagcagaaacttaaatcattaaacccacttcatatcgaaatagttgactttt |
| cagatggatgcggtctgaaatttgatgtaaaagtcgtctcacaagaattcgagggtaaat |
| cgcttgttgacagacacagaattgttcatagactgttggaagaggaaatgaaatcagtac |
| atgcccttactctaaaaacgctagctccttcacaatggtcgcttaaatcctcctaacacc |
| cttatcatcgggctcgataattggaaattgtcatatatttttgtaaataa |
| >TCONS_00001888 gene=XLOC_000947 |
| aatctgctttttcggaagatgggactaaaaattaattttccgtgctttaaaaatatcact |
| gttcgtgctcctcgatcatcattcccctggtgtcaaaaacaatatggacgaaaggtaacc |
| tccacccatccagaaaacaatgtctaccatgattatcaaaaccatatcacagaggaactt |
| atacaagataaacaagaaaacagtcaatctctcgtaaccgatgaagacgacaacaaaagt |
| gaccctcttgagaatacgaattcaagatactgttgcgaaaatatgtaagtgttttgtacg |
| aaaccgcaatatatttacgtttgaattccttttgaatagattaccgaggtcaagtacttt |
| tttgtctttatggtacaaaagaccattaattttcatcagaaacgctataaatggccgcac |
| tttttcccatgttgaacaagcctctgtcaatcatattcaagatatatataagtgacagtt |
| cactatagttgtttataaacagtccatttttattctgatgctggatgaaccagaagttgc |
| aatggtttttaagttcaaaatatatgccagatatccgaaaatgaatgatgaaacag |
| >TCONS_00001955 gene=XLOC_000981 |
| aattataacgatttagttttagaacttggttcgtaatttatagatgcatcgttcatgaaa |
| acttcagaattttcgaaaacactttcacagcctgagaggattaacggcgatgtgatttat |
| tgtcgcatatgcttggggtcgagtgactttgaggaattgataagtccttgctattgtgca |
| gataataggaacaattggaattgttcatcaacggtgtttggagaagtggttaaacttgtc |
| tcgatcaagagcctgtgaaatttgtggtttcacatttgaggttttaaaacattatcctca |
| cttttgcaaggtctgtctcatatttaactatgtcgacgagttttcattttcaatgtgtcc |
| actccacagaataaaatatatttccatagtctaagtgaccatgcattcgacttactgtct |
| ttgctgagaacactgaaagaccgtgttgggtgcacaagaaaggtgaaggctgactttttg |
| atggttttcataagcttttacaagagcgtcggatattacttcacttacta |
| >TCONS_00001956 gene=XLOC_000981 |
| ccgaacaattataacgatttagttttagaacttggttcgtaatttatagatgcatcgttc |
| atgaaaacttcagaattttcgaaaacactttcacagcctgagaggattaacggcgatgtg |
| atttattgtcgcatatgcttggggtcgagtgactttgaggaattgataagtccttgctat |
| tgtgcaggaacaattggaattgttcatcaacggtgtttggagaagtggttaaacttgtct |
| cgatcaagagcctgtgaaatttgtggtttcacatttgaggttttaaaacattatcctcac |
| ttttgcaaggtctgtctcatatttaactatgtcgacgagttttcattttcaatgtgtcca |
| ctccacagaataaaatatatttccatagtctaagtgaccatgcattcgacttactgtctt |
| tgctgagaacactgaaagaccgtgttgggtgcacaagaaaggtgaaggctgactttttga |
| tggttttcataagcttttacaagagcgtcggatattacttcacttacta |
| >TCONS_00000565 gene=XLOC_000281 |
| ttataactacgaagaggataaagtagcgggttcaaaaggcggtaagaacagaacgaaaag |
| ggatgtagaggaacaccataaaaagtcagaaaacaggacaaatcgtatccacattgacta |
| tcaagtcaataatgaggaaaaacaacgagaacaaaattctaaacatggctcggagaacaa |
| aaaataaccatatttgttcgtaatcacatattcgtaataaaacatttctatgcatcttgc |
| ttgtgagatgttcatactataggagtccttagtcttttttctgtagctgcattcatttga |
| caatcatgactattacggccttcgagtttttggaacaatgagtatgtgagaaacgtgcga |
| tcagtgtttagaagaatatataagattcagatcagatttatgctttagggacagaaagcc |
| tgagttaatattcgcattgtacatttctgtgggtgtcttatctccttctagttgcttcag |
| agatatgatcaattggtaatttaccttgt |
| >TCONS_00002017 gene=XLOC_001014 |
| gattggtcttgtttcacaagaacccactttattcgatctcaccataagagagaatattgc |
| gtatggagacaatagtcgagaagtgacaatggaagagattattgaggcagcacgtgcagc |
| taacatccatgattttattacaacacttccagagcaatatgaaaccaaagttggacaacg |
| tggatcaaagctatctggaggacagaaacagaggatagccattgcacgagctttagttcg |
| taaacctgttttgcttgtgttggacgaagccacttcagctttagacaatgagagtgagcg |
| tatagttcaagaagctcttgatgctgccatggggtcgagaacatcattagttgttgccca |
| tcgtctatcgacaattgtcaatgctgatctggtagtagtattacaggatggacgtaagat |
| agagtcaggacctccagcagcactgctcgctaagaaaggagctttctatgcattgcatca |
| catagagaattagattatctacctgaacttttctgagatcctactattttcaaacacttt |
| ataatatatctggaacacttttaattttttggtgtatctagaattcatttgtataaaaaa |
| >TCONS_00000604 gene=XLOC_000301 |
| caaaccgccatctaataaacttaaagtggagcaatctatcaggattaacacacgttcacc |
| aacctactatccttattactgaaaactggagccttgagccactctactaagtattggttt |
| ctgtgaagaacatttgcgtcttattcttgtagtcgacaaactactaggaagaccttaacc |
| caatttatcagggctgagttttcctagttttaatagaagtctgttatgtggtgccttatt |
| aaatgataactccgtccatctgtcttaccattaggttagtcgtatgcggtgttcctttct |
| tgaggccatttcattcactatgtaacaagtatgttggttttcacttaagtcatcatcttt |
| ttgttgattccttataatattttgactacgacaccactgaggttgatgagtctgcacttc |
| gagagtaattgtcttgatcctgtcctgtgaaatggagtgttagttggactctttcagtcc |
| acaggtaactctggctgaatgacgctgaatatcgtagtgagcagggtagcaatgtatggt |
| gcaattagtcccatgattctagtcctgttaacctctccgtgcttagagagtctagtgccc |
| ttgggacgtcgtcctggttgaagtctgcagcgagtagtcgatctagtaatattgattctg |
| gctctagtctttcgtcaaattca |
| >TCONS_00002051 gene=XLOC_001033 |
| ctgctttattggccacggcatttgattgaagattgtaatagtgctgtaacatccttttag |
| gccatcctacactacgttggagttttagaccggattattatcaaataatattgtcggatt |
| taatctttctcattacaccattggtgccactacatagagagaccttgttgaccaagtcaa |
| ctcatttgtggttgttaccttacagagcagcttacaacgccacgagtgtaacatgcaaag |
| gtaaaatttggagcagatgtgcgttcgtcaaggttacagattgtatgatgtgccactttt |
| aagcagattataacaactgtgaacacaaacctattaacatgctgacttatctgcatggat |
| attctatcaaaaattttcactccctatctgtttataatgcctgtaggaatgacttgcacc |
| tactatatattatgatcgttcataacattatttatattcttatctacgcaaatttacccc |
| cgctgtttctatgtcatattgtgtatcaacctaactgagtaacgtattacggtaaaccat |
| ataacgcctgtaacattttaatcttgcctgtaaactgtcatacattctattacatattgt |
| ta |
| >TCONS_00000625 gene=XLOC_000312 |
| acaatttcgtacatttgagctaatacaatgtctgactgctgtgaaggaaaatgtggttgc |
| ggatcgagctgtaactgcacctctggcacctgtaaatgtgatggcaaatgttcgggatcc |
| aaataaccaaacacactcatatggatgtggatcaagaaatcaacgagtaggagtacagtttcgacagcttgtcaacacaa |
| >TCONS_00000693 gene=XLOC_000350 |
| cattatagtctcgccgatttctgaaaagaagtagaaaatgaagacggaaatctatcctcg |
| caagcgattatgtacgtacgtaacataaaatcatagcatgcactatgaacgaattaactg |
| acgtcagttctcaaaataacctacatgcgacataagatgcgtaagggtttaactgagaaa |
| tggaaacaggtcatgtatactttgtgcagtaatttatcatttaaaatacaaccacaagtg |
| cactatgaaacatttaagatagtttatactacttttggttaatttttgtctactctatgt |
| cttacatcatgtaagtaagtattgtatattgaatccgtcttctttattatatcactctca |
| ccaaatgaataactagtcgagaacttaaaacatatgatcaatgatgttacccaatacaaa |
| tccttccgtaaagtaggtaattactaataccgatattgcttcttgttccatcctatgtgt |
| attgaatggttatttgctgtgacaaaattcaggccaacaatagttcagttttgaaagtat |
| ctgctccatgtttacttcttgacacaggtctccaacttgtgtagtcggttcgattgttcg |
| ttcatgtccagcattactttagtgatcgtctgcatcacaaatataatacaagtataaaaa |
| taaatattgatgtagccttattattatttattatttaaacataaatattggtacaaggaa |
| gcaccagatacatatgcgccgcctgtgaccgacgattgattcatacgccatttgttcact |
| caggataatggagcccatgtgcaccattggtttcgaatcagggttttccaactcccctag |
| gtggactcgccgtgtccaccaaaccggttaaagcgccgggcattcgcttttcgtcccctc |
| aatttcttaaacaacacccgtggtgcgagaaggcagtgagtaggacttccctgtcagagg |
| ctatatacgcgtggccatgtgagagcatttcgggaggaagagcggactctccccactctc |
| agctgtaccagggcattgatttagcctacttacttgacacagcctatattatgttagact |
| gtagatttgatgggctaagcggtgataggatttcagttttggaaagactgaatactaatg |
| acttactaaatatatacctagtcaacgataggtaagttgtctgtgcaaatattgacgttc |
| ggatactggactggattgcaacgaaggtctttttctatagcttctaattcctcggggttt |
| tatacgtgaccgatctctattttcctcggttctcttgcaatttttttattggacgcaact |
| acgagtgtacccacttggtctctttgaatgatacgataatgtgtcgtcctggtccatatt |
| ttttgtaagataattaaatagatttccgaataatgattccaggtaaaggcctacctgaaa |
| gtctcctgtagcaaaattgtgtaatcttacatagatacaataatgcgattattccattta |
| gcacactagacttctacatcaataaggccaattatttgcttatgtgttttatcccttttt |
| ctcaaccgtgggaaaccttgatcctgagggaatagatagtgtatgaaccaattattgacc |
| accggctaccatgggactgcatctccttacgttgctccactgccttgtggttcagacttt |
| taggtagagggctccgggtgtggccccttaagaaaaccacttaattcggtctgggcaccc |
| >TCONS_00000717 gene=XLOC_000360 |
| caagatgatttaagcggttgttcgtttgaagttcacggtaaagttcaaggtgtatttttt |
| agaaagtatgcggctgaattcgccaaggttaatggccttgtcggttggattatgaatact |
| gaaagtggtactgtggtaggtgaattcgaaggcccaagtgtaggtgtcgatgcattcaaa |
| cattggctttgcaacattggaagtcccaaatcgcaaatcgacaggtgccagttcaagaac |
| gaaagaaggatatctcaattacatttccaaagttttaatatacgtcgctagccagaacat |
| aatctttggttcttattaatccaataaccagcaacatataaaaatcagctagtggccaac |
| aactagacgtgcgtacaaattttgatggatttaatcccgctttattggttacttggttgg |
| gttatatacatagcaaaggctcttataaataccatttttttgtactctgcgggatcagta |
| aaaatcaatcttttgaatacaaaggaaggtggtatacgactatggatgactgagaaacat |
| taggcaacaacgtagcacagtaatagtgtcctcataatcgtagttcatactgataaaggg |
| acaagaaaaaggactcatatcaatgggaacgattatcagaaaacctgtcaattaaggcga |
| ggactgaaagggggggggatgaagcattttggggtttagagtttaattgctttaccgtcc |
| acaagcaaccaataagggggaaataaatggtagctcaagggaaataagagagacgacagt |
| aaatactgtggttcctcaaatttgtcattcttactatcccagtcatgtcatctcacagct |
| atttcgaacaacaaagccgagaatccagtccacctgcttaataatggggttctacagttg |
| tgaggttcagacaatgtctttcattgagtatcgtttggatatgatgttcccaacctaatc |
| gctctatgcaactgacccatacctgtcagcgggtacgaatcatttgactgaacagactat |
| tttcgattcatatttgttatatcagaagaataagtagatgataaccgccaggaattatgt |
| gtagactattattacatatgtccctattgggtaactacaaagtaactagattatatgtgt |
| attcgttttccttttatcgtaagctttcatttgacttattggctactgttgtacgatcta |
| ccacttaatttatccccggtctattagttacagtttcatatactca |
| >TCONS_00000718 gene=XLOC_000360 |
| caagatgatttaagcggttgttcgtttgaagttcacggtaaagttcaaggtgtatttttt |
| agaaaggtctgtacctagctaatataatcatctgtacttagtatgcggctgaattcgcca |
| aggttaatggccttgtcggttggattatgaatactgaaagtggtactgtggtaggtgaat |
| tcgaaggcccaagtgtaggtgtcgatgcattcaaacattggctttgcaacattggaagtc |
| ccaaatcgcaaatcgacaggtgccagttcaagaacgaaagaaggatatctcaattacatt |
| tccaaagttttaatatacgtcgctagccagaacataatctttggttcttattaatccaat |
| aaccagcaacatataaaaatcagctagtggccaacaactagactttcgtcatacgtactc |
| gcctaggaatgaaacttcatgtctaactatttggagatcgatttcctgtaaggatacaaa |
| catcgggcacacgaacaattcagatcttcatcagcagaaaatatcaggactaaagtttta |
| cttcccttctgagaaa |
| >TCONS_00000719 gene=XLOC_000360 |
| atttaagcggttgttcgtttgaagttcacggtaaagttcaagaaaggtctgtacctagct |
| aatataatcatctgtacttagtatgcggctgaattcgccaaggttaatggccttgtcggt |
| tggattatgaatactgaaagtggtactgtggtaggtgaattcgaaggcccaagtgtaggt |
| gtcgatgcattcaaacattggctttgcaacattggaagtcccaaatcgcaaatcgacagg |
| tgccagttcaagaacgaaagaaggatatctcaattacatttccaaagttttaatatacgt |
| cgctagccagaacataatctttggttcttattaatccaataaccagcaacatataaaaat |
| cagctagtggccaacaactagacgtgcgtacaaattttgatggatttaatcccgctttat |
| tggttacttggttgggttatatacatagcaaaggctcttataaataccatttttttgtac |
| tctgcgggatcagtaaaaatcaatcttttgaatacaaaggaaggtggtatacgactatgg |
| atgactgagaaacattaggcaacaacgtagcacagtaatagtgtcctcataatcgtagtt |
| catactgataaagggacaagaaaaaggactcatatcaatgggaacgattatcagaaaacc |
| tgtcaattaaggcgaggactgaaagggggggggatgaagcattttggggtttagagttta |
| attgctttaccgtccacaagcaaccaataagggggaaataaatggtagctcaagggaaat |
| aagagagacgacagtaaatactgtggttcctcaaatttgtcattcttactatcccagtca |
| tgtcatctcacagctatttcgaacaacaaagccgagaatccagtccacctgcttaataat |
| ggggttctacagttgtgaggttcagacaatgtctttcattgagtatcgtttggatatgat |
| gttcccaacctaatcgctctatgcaactgacccatacctgtcagcgggtacgaatcattt |
| gactgaacagactattttcgattcatatttgttatatcagaagaataagtagatgataac |
| cgccaggaattatgtgtagactattattacatatgtccctattgggtaactacaaagtaa |
| ctagattatatgtgtattcgttttccttttatcgtaagctttcatttgacttattggcta |
| ctgttgtacgatctaccacttaatttatccccggtctattagttacagtttcatatactc |
| a |
| >TCONS_00000814 gene=XLOC_000403 |
| tcttatactgcagaatcatgaataaatcattaggagaaggtagtgatatagacgattcgt |
| ctcatctgagaaagccaactacgatagatgagagttgctctagttgtcgtgcagtcagct |
| caattgtccctttaactctatctgcttatataatgtatgtgtgtaaaggtcaagcctcca |
| agtatgctggtgtaaaaaaagtgtcatatttgactctatgcactagtatgtcattgggtg |
| tttactgtacattggagggagtcagttgttctctcgatgaaatgcaaaactaccaatgaa |
| agccttctagagttttctatgaatgaatgtatataacattttgtcttttgtaatgttatt |
| ttacctcaaaatagtattgttttccaacatt |
| >TCONS_00000821 gene=XLOC_000409 |
| atgctattctattgatactcttattcaaatttgtttataacaacacatcattatattcag |
| cttgtacaaaaaggttggaaatattgtttagacattgtttgctttccagtttaatttcaa |
| agcgttgtcttggtttgaacgaaggtgtcatttcatcttcttatagtggcattgaagaat |
| tgaagaaactttgtaattcatgtagaggctgtgaagtttcatttatgagttgttcagtta |
| ataaactggactctattgacaacagagaatgttcacaagctaaaatgtattcaaatacgt |
| ttcgt |
| >TCONS_00002267 gene=XLOC_001150 |
| tgggaatgaagatttttgcagtatttggtattatgttaacttatttctgtgcagtaactt |
| tcggagctgaaagtcttcatctatgtaacgaaaaacttagggcaacaatggaatactgtt |
| ttcaaaagaacggacgttattcaataccgttgacggagttaaagagattgagagatgctt |
| gcatgaatgatagaaattgcaaaactaaagcaaaggattgtcttctatcaaaacttaaaa |
| gttcggaactcggaaactgtccttcacttaggacctatatacaatccatagatcgtatgt |
| tcaaatagacttctggtcatctcttagtccactatccaa |
| >TCONS_00000940 gene=XLOC_000460 |
| cggatgttcgcactcagtgaactttgacagtagctgcttcatatttgcactcaatttaga |
| aatacctgacggtgactacctacacgcctagattcgaacgttgatggcaacgtatctagt |
| tgtatggaactactcaattcacatctacaaaataaagaaacctatttggcaatcagcgag |
| acagcactgtcgaacaaccgaaatttgggatatcaaacgcggctattttcagcctgcatc |
| tcagc |
| >TCONS_00002298 gene=XLOC_001165 |
| ttgctcagtgcggtacactgtatggtaacctactttcctgtcaaagtacatcaaaacgtc |
| gatgtcagaaactcccgacagattgattagtcttgaggagttggaaactatgtttaagga |
| caggtatacggacaaggatgttaactatcaaaagtatttgtcgaatgccaatgtgccacc |
| tcctatcatacctcactggggtactcttgtttgctattaagttaaaacgaattctagatg |
| aaataaatcgagaaagattcaatcatcggagtcagagaaacaataactacagattccaaa |
| gaaataatttccgacgcaacagtttggattaattcgattcattatcagat |
| >TCONS_00000950 gene=XLOC_000465 |
| actctcatgttctttatctacctcctgataatgtcaccaatttttggttttatgccgtcc |
| tggtagcaatgataattttttcatatttcgtcgcgagagggataagagaaatttattttg |
| aaacaataactccaaatttatgtcgtgttcaccaatcacataaaccaacaagttatggtt |
| ctgatgaaaatggaacgagaactggcccacattcatgataaactgccttctattaactat |
| ttcggtcgatttatttttcgttatgtattccgaaagttgtctaaaattactggtcatgcc |
| attttcctttcaatttatttgtaagaagatgggtatttcccaactactttacttctaaaa |
| atactactgttaccaggaatatttacctattgtcactaaagccatggttttggatatgta |
| tatttcacacatgaagagaatt |
| >TCONS_00002329 gene=XLOC_001176 |
| aatatattttaaacatactctatataggtacgagctaagattgaaacacggagattttga |
| atggagtgtttttcgcaccctggcagaattaaaatccttccatacttctttcatgctaca |
| gagagctttgttatctataagtaatataagatagccgcagaaaactcaatccgtttcctc |
| caacttttgaatttgccaatgataagagactatatgatcgcagggttatgttggaaaagt |
| acattcagagtattattgatgtacgagaatatcgagaaacagatcttgttgtgagtttat |
| cgttccagttttgcatgtaatgaagttgtcttgatgtttgggactgaaattcgaggtttg |
| tttcaactatcaaattggaaaatgtatacatgatcacagacatttaatatgacaaggacc |
| caatgtagtttgtgtgtaattaaatgtaaataaatccaaacactaaaacttccgtagatt |
| ctcatcacaattggaaggcgataaatgattagaatttcaaaacgagattggatcaactga |
| gagatcaccattgctaggattaacacaggatatctaatcccccttccctttcaaactgaa |
| gttagaactaactgaatacaaactttaaaattttaagctcctgggaacttaaacatttcc |
| caagaatcttcaactccct |
| >TCONS_00001011 gene=XLOC_000494 |
| tgcggatcacgcggttcccttcctgtcccagcgtcccatttgctcttattctgcgagttt |
| tggttagacgagtgcctataaatcacagcgagtcgattgcgaagattgtttggaaacata |
| tccaatgaaaattgtagttaaatactaggagtcgtcctaccgggagattcacttattgac |
| tcggaataccccaataatatagttctgtttgaagatgctgacaaagtgcaatcttctaac |
| cacattaagtaataacgcaagcatgtttcggatgcggttctaaccctccaaatccaaaat |
| gttacctcaggattaacctgcgttcgagtctgagttagtcatagggagtgaattattcgg |
| acgtatcgaccgcctcacttatcttggaagtgtcatcagcagtggtgtttgaagaaatct |
| cagcacgaattcaaagggttttattggtatttgccaacttgctgtgtggggaagatatcg |
| tttatacttgcttgatgagtacatttatcaagtgatcataattttttctgctttgacaat |
| cctaattttccgagagtttattattatgta |
| >TCONS_00002399 gene=XLOC_001216 |
| agggcggtacaatttgtggtgattgtcacgttgtttttggagtactttttagcatggacc |
| ctactagggtggttgaggcattaagtaatactctattggcagaaaaacaagaaaatggaa |
| caaagatactcgatgagatgcataaaattattggatttgttcccactctattgaaaatca |
| tccttgaagagactgttgatgtgggagtacgtcaagcagcagctctttattttaaaaaca |
| acatctctgaatggtggaagcccgatgagccggatgaacctggcgagttaagattttgta |
| tacatgagcaagataagcaagcaatcagaagttctattgtagcagctcttgtttcggcgc |
| caattcctttacaaactcatctccaggtcgccctttctaaaataattaaacatgattttc |
| ctactcgtttcgaagagttccctgaacaggtgaaacgtttcttagaatcaaatgatcgtc |
| atcatcttcgaggagctcttcgctgcctctatgcttttatggaagtttatacatacaaga |
| aaaacgatgagcgtgcaaacacggtttccgcgatgcaagtttttttcccaattttatatt |
| caacgttgtcgagtctcgtcgttgaggaatccgaggattcttatgtacttcaaacactca |
| ttatcaaaatattcttttcatttgtaaattatcacttcccactggatgcaatgagtaaac |
| agttgtttactcaatggattgatatcctctgtaatgtactcggtgatttcaaagtaactc |
| attcggacacttcaagttctacctggaaacgacaaaaatgggctttaaaaatacttaaca |
| gggttttcacccgttatggtagtcctggttcagtcattaagctatatcaaccattcgctg |
| actggtatcttaaggccttctcagggcaaataatatcagtacttctaaatatatgtgaag |
| cgtacagacaaaaatcattcgtctccaaacctgtgcttagtcaaacgctggactattttt |
| cttcggcactggctaactcattttcatggaagttactacgaccacatttttctcttttgg |
| tccgtgaagttatattcccactaatgtcatatactgaagaagatgcagaattatggcaag |
| acgatccaatagaatacattcgagctgaagctggtgattggggttcggtatctagtccag |
| catgtgcagcatctacacttttaagtgaagcatgtattaaacgacgtggtgttctcaata |
| atataatgccgttctgcatccatatattaagttccgagagttctcctgttgaaaaggatg |
| ctgttttgcacatgtacatagctatagctgaaatcttactaaaaaaagctgcatacaagt |
| cacaattagaatcatttcttgtcggtcatgtccttccgactttgcatgctcctgaaggat |
| atcgtcgtgctagggcttacagattgctggaaaaattatcagaagctaaattcaacgacc |
| agaatatttttgcccaagttgttgacgaagtaagaaaagctgcatgttttgattcggaat |
| tacccgttcgtgcgtttgctgcactctgtctctcagaattagttcggtgtcaatatttag |
| ctcatcaatttgtggctcctcatctacacgaactctt |
| >TCONS_00001137 gene=XLOC_000568 |
| aatgtcattaaccagattatatcacagttgacaagccacggaacatttaaccaggttttc |
| agaggaatccaagctgtttcactaacagataactgcctaacatgtagattcaaagttaca |
| aattctgaagccaactcactaagcactttacatggtggatacatccttggggctatcgat |
| tttataacttctgtcgatctgatgagattagggtgtatgaaacacgttagtgttaatctt |
| gaagcatcatttataaacccaggaaaattggattcatggattcggtcagattcatatata |
| ctaaagaaagggaaccgtatagccttctgtgaaatcaaattcgtgaacgaacagtcaggg |
| gagctagttgcacgtgggactcacacaaaatacattatagaagagggaaacctatctcat |
| aggaagtagcaatgtcaacttgtttcttcaatccttctcttgctttactatatgtcacgg |
| ttggttagaataaa |
| >TCONS_00001180 gene=XLOC_000592 |
| ctttagtatattcttaattgttttatgtttaacaatgaaaaatgtttattctatcacctg |
| ttacgtatgcgaaaattgtttgagcgtagacggaagtacttcaactgaatctggctgcgg |
| aggatgtattcgaactggagtgaagggaatttatgttaaacgacaatgcgttgccaactg |
| ttcagatattgcagctaattttcctgttaaagatcttttatcatgttgtacaactgatta |
| ttgtaatcattcaagacaattgaaaccatttattactatgggtttaataatttactctgc |
| ttggtatatatttaata |
| >TCONS_00001276 gene=XLOC_000636 |
| taaagtggtgataaacaactttgtattatagaagttgtatttaagacaattatatctatg |
| cttatataattttcaactctgcaattatcgtcaaaatttcatcattcaactgcaacatat |
| tattacgtcctgtatttcttcgattatgtcaaagtaattcactatctaattttccattgg |
| agaaatataatgcttatgatatattaggtgtaaaacaatctgcaacttctagtgaaatta |
| aatccgcttattataatttgtcgaaatgtttacatccagaccgattacttcatactaatc |
| cggagaaattgaggaaggaagaatttcagttggtcacaactgcctatgaattacttcgtg |
| atcaggaaactcgatcacagtatgatcgatatttagcaagtagttgcttgatccaaacag |
| ataaaattgataaatcggcaaatttcgatgctgaatttcaacatgcacataagttatata |
| tgcgacaccggaaatccaaaacatataaatcatatcgatgtggaccggatgaagttgtgg |
| aagaatttagaagtaaaattaaaacaaacgtctatcctaaaaatatatcggattattatg |
| cacgtcaacctcaatcacatatatacactccatcatttttattaactattatgttaagta |
| cctttttagtgt |
| >TCONS_00001280 gene=XLOC_000638 |
| ttgctaatttaaaggttcagttttcaacttaaaatatactcctaatccttggtttattaa |
| atttatttggaaggcactaagacattggtttcgtgaaggatttcgggtaacaatatatct |
| tggagttccattcacaatgctttttttaagcaatttgccatctacgccagaatacgttca |
| taattgggtgagaggtcgttatttgaagtcaacaggaaatgatataggtgaattcggttc |
| tagacctccagtgaggccgatcgattaaatatgatgcatgcagttttcttcttatactat |
| tgtaaaatgaaaagagtgaccggtagactttgttggtacga |
| >TCONS_00002752 gene=XLOC_001400 |
| cctgattgaatcatattctatcaataaaacgttgatatatgtttgtttctttattccctc |
| tctctctctctctctcatgatcgttcgaaattttaggtaaaacatggtttagaaagttta |
| tgtaaaaaagttgaaaaacatttatcggatgaagaaaatttatttcctgttgtttggcgg |
| tcaatacaaacagaatttattacacaatgtttacgttttagtaatttaattcaacaatgt |
| tatcctgattctggtatccatcttgaatttactatcacagatttacaaaattattttact |
| gaaattgcatgttcacgttgaaactatgctttttttttcatttccctccctcacttttac |
| agtttgtatatgcaataaaactgtacttatatatttgtatattgttgccttagctacctt |
| attgtttacccacctgagttacaaatcttctccaccaccttactgttcaaatacacacac |
| acacacaccatatcgctgatggataattcagtttctttagcgcacaacgaatatgatttg |
| atcaagcaagattcaactggtgaacatcaccttgatctggcatgaaaccatgcacataca |
| tacggaattctacagaaattgttatttcttattctcagttttttgttttgttttaatttt |
| gttttgtaatttttttcgtttgtttttttaacttactct |
| >TCONS_00003004 gene=XLOC_001638 |
| agaagttctaaaatcatagtacatccggtacaggaactcatccatatggctccatagtagtcggcctctg |
| gagccatgataaggtctcaaaaattctttcagcctcgaccacatagcctcaatgttgttggtgtgcaccc |
| cagttgtagggtctacaaaatggcgcttgtgtataacaacttcatgcacgtagctaagtctacttaggca |
| cgtatatgctctccaatcgtctgtatacaccgtagtgcccggctacacatattgctgtataatcggtgtt |
| gttgttggtgcatgtcggttcctaa |
| >TCONS_00003599 gene=XLOC_002172 |
| ccttctaagattttcccaccttttgtacacccatccaggacacaaattatctttaggcca |
| gaatctaaccaaacgagtggtgaatattatatataatgttgtttttttcattttttaaaa |
| ctttagttgggaaggatgtgttaattgaactgaagaatgacctatgtatatgtggtacgt |
| tacactcggtcgaccagtaccacaacttcaaactcaccgatattagcgtaactgatcctg |
| aaaaacaccctcatatgttgtccgtaaaaaactgtttcataagagggtctattgtgagat |
| atgtccagctccctgctgatgaatgcgatacgattgaatta |
| >TCONS_00003600 gene=XLOC_002172 |
| ttgaaagactgacttttttttaccgtgccttctaagattttcccaccttttgtacaccca |
| tccaggacacaaattatctttaggccagaatctaaccaaacgagtggtgaatattatata |
| taatgttgtttttttcattttttaaaactttagttgggaaggatgtgttaattgaactga |
| agaatgacctatggtgagtggtacgacatatttgtttcatataatacagtatatgtggta |
| cgttacactcggtcgaccagtaccacaacttcaaactcaccgatattagcgtaactgatc |
| ctgaaaaacaccctcatatgttgtccgtaaaaaactgtttcataagagggtctattgtga |
| gatatgtccagctccctgctgatgaatgcgatacgattgaatta |
| >TCONS_00003616 gene=XLOC_002180 |
| tactctactcagtttgatactgttagagggcattcagtgttagaatcatcaaaagttata |
| tttggatatggactgaatgtcatgtttccaaggaaatggaggaaacatgtttttacagta |
| aagaagactgtggaaataaaattatcagtgatatcctttcgatattgcatacacggatgg |
| aaaagaagctaaatgaaagactggtattatttggtaacgacaatcaagatcaatctcaaa |
| gcaatcattcagttggagttgaaaagcttcaaaagaggaaaaatgaactttctacggaac |
| ttagaaggttacaggcatcattcacagaacatcgaacttgctaattcacgttgtgtttta |
| caaacagaggtgaaggaattagaagcattgcttgctacatatgaaaataaagatggcgag |
| tatcacaaattagtaaaaagatatgttgattgtcaacaacaattatctttacgttctaca |
| ctattacacgacactgataatgtacagc |
| >TCONS_00004037 gene=XLOC_002464 |
| cagcaacttccctcagcaaacgataaaccctgaagcgttgccgaacgttaacttcgtatt |
| attccaacttcctatcacaaatagatctattcatacataaactaaatgacaaactttaat |
| gtaatatgaatcccgattgtcgaaagcccgttttattatggggtggtcttggcgggttta |
| ctattggaattgtcgattttctgcgaacaagtaaacaacgaataatttttctacaatttc |
| accaccttaggtaacctccgtcgtggtgtgcaagttggtttagtgtccatattatttata |
| tccacatgtgctaagtaaatcctattacgtaacataacgttactccagtataatatgcca |
| tcatttaaagttcaagcgtaaccaagaaggtcggatgtcatattttgattttaaaaagcg |
| gttggatgaagaactagctcaaaagctttcgttatcacaaaaaagaatgtgatgaaagac |
| ctttaaatgattgtagactatgtataataattcgaatc |
| >TCONS_00004038 gene=XLOC_002464 |
| gataaaccctgaagcgttgccgaacgttaacttcgtattattccaacttcctatcacaaa |
| tagatctattcatacataaactaaatgacaaactttaatgtaatatgaatcccgattgtc |
| gaaagcccgttttattatggggtggtcttggcgggtttactattggaattgtcgattttc |
| tgcgaacaagtaacctccgtcgtggtgtgcaagttggtttagtgtccatattatttatat |
| ccacatgtgctaatataatatgccatcatttaaagttcaagcgtaaccaagaaggtcgga |
| tgtcatattttgattttaaaaagcggttggatgaagaactagctcaaaagctttcgttat |
| cacaaaaaagaatgtgatgaaagacctttaaatgattgtagactatgtataataattcga |
| atc |
| >TCONS_00004194 gene=XLOC_002566 |
| aaggtctacgtaaacgttctcagttctacccattcggctacgattcccgcccttacactg |
| atggatcacctttgaaacgtttgaagcgaatacttcatatttcttcatcttattatctca |
| tcgaaatttgtcacaaatatgtactctgctcctgacaatcgccttagttgttcgagcata |
| tgtgatgaccagtcagtttgccgagactatttgcgaaacgtctgtaaacgtggcaaaaaa |
| tgcaagttcaaacacccatctttggaagaatgtgaaaggttacgtcgatttgacaaaact |
| ttctgccacgacttccaaaattcaacatgtcgccgaccaaactgcaaatttttacactac |
| acaaaagaggaagaggaaatgtttcgacgcactggttatttacccaacgatgtacaatgg |
| tcatatcgtcctgcttcaggcacatttgatgagaaaactccaatttgcaagg |
| >TCONS_00004933 gene=XLOC_002970 |
| taaccgcgcttttgaatcctcaacatgggttcacacaaaacgttcaggatgaaaaccaag |
| ctcgggaaaaaactgaaacaaaacagaactttgccttattgggttcgattaaaaactggc |
| aacagaataaagtataatgccaaaagacgacattggagaaggacaaaattgaaattgtag |
| ttgtttgacctttc |
| >TCONS_00004966 gene=XLOC_002995 |
| tgttcttcttattaaaagaatctgtttaaatcatcaagttgtcattccaaacgtttgtca |
| tctttgaataatggccaccgtgtctaaacacaagaaagtgagaatcatgaagcagaagtt |
| aaaacttttccgagccaatgaaccattaaagagtgtactgatgtggggcataaactattc |
| gttttctgctttaaatcatgttaagcaacgtgcactgttattgaaagacgatttcaaagc |
| atatatgaaagtgaaggttaataaccaccattttaacaaggagaatatgccaagtagatt |
| caagtttaaggagtactgcccaatggtgttcaagtcattgcgagatcgatttggagttga |
| caagatggat |
| >TCONS_00005018 gene=XLOC_003021 |
| ttcaattatgcttaaccgacaatttcaattcagaattatcttaattttccttattgttat |
| ctgtttatattatattaaaggatttactattcataatgaatatcttcaaaatgaaaagtc |
| attggatctatgtggtcgttgcaaaaagacaattggtaatataataaatgatttatcaaa |
| ttttgattttcaattgaaatgggaaataaaacttgtcaatggatgtaaatatactggccc |
| gcttcgtgataattgtgcatcattattcagttcaacaatcattaaaattatcaacaaatt |
| tataggaaatataaaggctgacaaagtttgtgaggatatatttgtatgttctatataagt |
| atcgtcatggtacaatcaattaaatcagttctttttataccgacaacttttagccatttg |
| aattttttctttcaacaaagtttactactgaaatatacgtaaagtagtgtaaagttatg |
| >TCONS_00005092 gene=XLOC_003061 |
| gtaaatgattcaattgttagtcaaattggtcgtggaatacttgttcttattggcttatca |
| agacgcgatacagaaagtgatatggagtatatagttaggaaaatactgaacattcgttta |
| ttcccatcactagatggaataagacgttgggataaaagtgtaaaagatttaaatctggaa |
| attctttgtgttagtcagttcacattatatagtgaattaaaaggtaataaattagatttt |
| cataatgctatggatccaaaattatctaaagatatttattcacaactgattaatcgatta |
| aagaagaattataatgaagaaaaagttaaagatggaatttttggcgcaatgatggatgtc |
| agtcttattaacgatggacccgttactattacacttgattcaaatccagatattagtcga |
| aacattacaatgaattcacaagatgatctagaaacttcgaatacacaataacttaaagct |
| gtagagttatgttggttgtttaaatttgtaacatttgtaaaaagaa |
| >TCONS_00005145 gene=XLOC_003087 |
| tcatcggttaacaatttactgacttaagttactgccgttcatggaatcgcaatgattgac |
| gatgtatagtgaattcagtagtaatacaggatcttcttcacagactactgctgtcatcca |
| ggtttttgaagatatctcctcgaaaatacttaaaatacgtttactggcaaatgagttgga |
| aacgctgaacaaaagcataagcattagaggaaattcgaaggctcaatccgaccttctaaa |
| taaaagagagaaagatgctctcgacctggttgacgacactaaaaacgtgttcattagtct |
| ttcgaagcagcctttatcggatcagataaccaaaattcatgttgagaagctcaaagttga |
| ctttcagcgtgtacttcaccggctccaaagaactcaagct |
| >TCONS_00005182 gene=XLOC_003099 |
| cctctacttgaccttgaggacctgtaaacggtctaccgatcgtcatgaaagtggttttgc |
| agccaactgagctgatcgtgttcgtccgtgcaataaattctcttggaaaactgggacatg |
| aggtttactttgaatgcggcccaaatgatttaaaggtcaaaacagtcaacgcctcgaggt |
| cttcttttgcttcggttcactttagagaagtgtttttcgacaagttctcaagccctttac |
| ctaacgggtctctgcaacgttttaaaatccctagtagttcatgttccaacgtctttagac |
| ttacttccg |
| >TCONS_00005213 gene=XLOC_003114 |
| ggagattaattatgaaactgtaaacataaggcggagtttcctgcttcttccagttataag |
| aatagactcatagtcattcattgtaaaacaatgaactcatcagaaggtttgattcagcat |
| gctagaggaaattttagagaaagcttcttatcctctgaactgggagaaaaaattggtctg |
| gtgcgtgaatggatcatcggtacgggaacaaagttattggactctcagacttctaaaccg |
| tataattttgaagaagcctgtcagattcgtaaatcacacgaacaattcgagtttaagtgt |
| atgaaagctttggaggtcttcgctgaactttgtgattacagaaagcaaagtagcatcaat |
| ttggaactgagtgagctggactatatggtacagagttttgtggaaaagctaaataggaga |
| acgttttttgttgtatcgtgctatacttatttcagattggttgaagaactttggaatatt |
| ttggaggagttacaaaagagggaaaaggaggtcgatacctacgatggaacgtccataaga |
| aacgcgatatctgatttagagcacggagttgaaaagtgtgaagtgagactaaacggtctt |
| gctcatgaacttgaaagactgagaataattttgaaaccttctgatccggattatcttgga |
| cagttggaagatggattcagagacgcatgtcgagctgccactgaagcaacacaatccctt |
| aaaattcgcaaacttgtcctcaagaagcactccaagttacttgaatgtgaggaaaatgtt |
| tacgaatcaatcggttggatagaagagctttatgaaaatattgaagtaatgtatgaagaa |
| aattgtgttggaaaaaaccagttagagtccgaagatcttttgaaaaagcataatgatata |
| >TCONS_00005214 gene=XLOC_003114 |
| ggagattaattatgaaactgtaaacataaggcggagtttcctgcttcttccagttataag |
| aatagactcatagtcattcattgtaaaacaatgaactcatcagaaggtttgattcagcat |
| gctagaggaaattttagagaaagcttcttatcctctgaactgggagaaaaaattggtctg |
| gtgcgtgaatggatcatcggtacgggaacaaagttattggactctcagacttctaaaccg |
| tataattttgaagaagcctgtcagattcgtaaatcacacgaacaattcgagtttaagtgt |
| atgaaagctttggaggtcttcgctgaactttgtgattacagaaagcaaagtagcatcaat |
| ttggaactgagtgagctggactatatggtacagagttttgtggaaaagctaaataggaga |
| acgttttttgttgtatcgtgctatacttatttcagattggttgaagaactttggaatatt |
| ttggaggagttacaaaagagggaaaaggaggtcgatacctacgatggaacgtccataaga |
| aacgcgatatctgatttagagcacggagttgaaaagtgtgaagtgagactaaacggtctt |
| gctcatgaacttgaaagactgagaataattttgaaaccttctgatccggattatcttgga |
| cagttggaagatggattcagagacgcatgtcgagctgccactgaagcaacacaatccctt |
| aaaattcgcaaacttgtcctcaagaagcactccaaggtttgttgacgatttgtatttaaa |
| aggatagcatcttaaaacctttgtcaaaaaatacatacgggacaaacaatcttgacaaaa |
| ttttaaaaaactgctaagtatcaaaaaaactattcgatattttgatgacttaaaacttt |
| >TCONS_00005215 gene=XLOC_003114 |
| ggagattaattatgaaactgtaaacataaggcggagtttcctgcttcttccagttataag |
| aatagactcatagtcattcattgtaaaacaatgaactcatcagaaggtttgattcagcat |
| gctagaggaaattttagagaaagcttcttatcctctgaactgggagaaaaaattggtctg |
| gtgcgtgaatggatcatcggtacgggaacaaagttattggactctcagacttctaaaccg |
| tataattttgaagaagcctgtcagattcgtaaatcacacgaacaattcgagtttaagtgt |
| atgaaagctttggaggtcttcgctgaactttgtgattacagaaagcaaagtagcatcaat |
| ttggaactgagtgagctggactatatggtacagagttttgtggaaaagctaaataggaga |
| acgttttttgttgtatcgtgctatacttatttcagattggttgaagaactttggaatatt |
| ttggaggagttacaaaagagggaaaaggaggtcgatacctacgatggaacgtccataaga |
| aacgcgatatctgatttagagcacggagttgaaaagtgtgaagtgagactaaacggtctt |
| gctcatgaacttgaaagactgagaataattttgaaaccttctgatccggattatcttgga |
| cagttggaagatggattcagagacgcatgtcgagctgccactgaagcaacacaatccctt |
| aaaattcgcaaacttgtcctcaagaagcactccaagcatcttaaaacctttgtcaaaaaa |
| tacatacgggacaaacaatcttgacaaaattttaaaaaactgctaagtatcaaaaaaact |
| attcgatattttgatgacttaaaacttt |
| >TCONS_00004568 gene=XLOC_002771 |
| cttaattttgaccgagagaaaaagtccctgttcgagctgatttcttcttccaacatgaaa |
| aacatcgaaagataatatcatcacgatagtctgcacaccgtacaatggtcatttgactac |
| cgcagtcacaagtataggagcttcttaataatccctcttattgtagacgctcaattatca |
| agtcttctctaaaatccactgtaaagcgatcgtacgtaagcatcatgttccgaaattagc |
| gccgtgaccttaaaaacccgcgagggcttctgattggctcgtccatatagtttagtctcg |
| ccgacttaagcgagacttttcaacgcagaatactgatcaggcgttttggaagctggactg |
| aatttggtggtgtcttaaaggagtttcaaaaaactacccatactcattacattcacggcg |
| aaggtaaactgctgaaggacataagatataaatacatgtttggtgtttcattgctcattt |
| ggtcatagacttaaacaacaagatctaacagtgaataaaaaaccctgaaaactgtaacta |
| acatgtgacatttaaggtctaagtttcgaaagtgctaattaaggttccttgtaaacttgg |
| acaatgaggaatttgtcataaaatcatacaagatgcgtcacaatcatccttgtaatagtc |
| catggatggtttgtgatccatgcaccagaagattgtcctcaaaagaaaatttgaagccgg |
| tattaaagatcgaactggtaagcaggttactgctgctaatgtggagactatggaaattca |
| actcttcacagtatttgatatattgcggcaaaacagtgaactaacagagtattcagaata |
| cgaatgtcgccagaatctgttttagtacttatgatcagataggatcatataacaaatatg |
| cagaagtagtttgtatcaatccaacttacaacaccaataatagaaggttctttttcagtt |
| ctactttgttgatattcgttattctagttggtaataagggataattttggactggagcgt |
| cctgttctgttagcatggacgcaaaaagaatttccagcagatgtaacctggatcttggat |
| aagttcagaaggcgaactcttagagctcatcatattcttagacaatgcaggaggataaat |
| aacacacataacttccataataacacatcattacccagacggagtaaacaatatatacag |
| attcagcgaatccaaatgatatgcacaaacaacagattattgagaaatatggtgaacact |
| ttgcgatgatggtgtgaaggagagtcgacaacttttacctaaggatcatcgataactagt |
| attccttacgatagaaaatacggatcggagtacaacaaagtgtggagacgtgacaggaag |
| tgtttactataagaactttgtagtagtattttaacttaactacatatccaacctgtaaac |
| tgatttttctgcatcaaatgttgctgtttctgtataagtagtagtgatacatc |
| >TCONS_00004569 gene=XLOC_002771 |
| atataaatacatgtttggtgtttcattgctcatttggtcatagacttaaacaacaagatc |
| taacagtgaataaaaaaccctgaaaacttgctaattaaggttccttgtaaacttggacaa |
| tgaggaatttgtcataaaatcatacaagatgcgtcacaatcatccttgtaatagtccatg |
| gatggtttgtgatccatgcaccagaagattgtcctcaaaagaaaatttgaagccggtatt |
| aaagatcgaactggtagttgtctgaaagtatgttttaggttgttataaacgactgcaagt |
| atttgatatattgcggcaaaacagtgaactaacagagtattcagaatacgaatgtcgcca |
| gaatctgttttagtacttatgatcagataggatcatataacaaatatgcagaagtagttt |
| gtatcaatccaacttacaacaccaataatagaagttggtaataagggataattttggact |
| ggagcgtcctgttctgttagcatggacgcaaaaagaatttccagcagatgtaacctggat |
| cttggataagttcagaaggcgaactcttagag |
| >TCONS_00004675 gene=XLOC_002833 |
| cttaaatcaatttacaaatcactggataccaacgctgaacctgacgctttcagttttatg |
| ccgtacatatctggttcgtttgaaaataaacttattaagatatctgccggtctattaaac |
| ccaccatacctcaaagaaggatattcaatgtctgaaaaatatgggactattggttggttt |
| attggtcgccagcttatttatgaagggaataataacaaggaagaagataatcatgtgaat |
| tcgcagtcctcctgtttatcaaaagaagcaacagattctgaagctcaaatatgttgcctg |
| caggaacaagacagcttcaaagactacaacaaaacggatttgagatctttgtcagccgat |
| attaatggtctcatgttatcatttaaaacatacgcgagtgatggagatttggatggaaag |
| aagttgtttttctcatcgtttgcaaaaatggtgtgtactagatactccgcagatgtcatt |
| cacacaaacttacagtcttccaactccagatacaaattcagtgtgaatgaaattctgaaa |
| cacagtcaagaattttccaatatacaccaatgtccatctggttcaaaaatgaatcctgag |
| aataaatgtctcttgtggagcgaagcataaatcatccgtgtgtgtatttggaaatgtctg |
| cttccgtgtgtcctcactttttttgaactttacatgttttcttttcataacaataaacat |
| atttcgtaacatgacaaaattgcacatgaatatatgatattattacacctaga |
| >TCONS_00005477 gene=XLOC_003258 |
| gtttcagtcagatgggcgaggacttgaagtcagaagatatacttggatttaaacttgata |
| ggtgtatttcagataccttaattaagacaggtgctggtttatttgggggaatcatatttt |
| ccgtagtgcttgctaaaaggcgtccatggccgcttattttcgggactggttttggtctcg |
| gaatgggaatctccaattgtaataacgattttaaacaaccacttcctttagtttctcacc |
| gtattgtgagttcaaatcaagttccggaggaaaagaaaagctcatagtactatggcacaa |
| cccttctagtctgtatttaaaccaaattgtttttctagaaatgactttcgactagtagat |
| tgttatgtgaataaagc |
| >TCONS_00005484 gene=XLOC_003261 |
| caatcttcaagtagaaacgtgctttgatcttagattttaacgtactaactccaggacttt |
| tcgatctcatgtctcaccaaggagctgcacttcaaaactataataatgaacttgtaaaat |
| gtatggcattacttgcttactgatcgaaattttaggctttgaagagctttgtaaaagacg |
| agaagatttacagcgacagatccaacaagatgaaactgaacgcgctaaacttcaaagaga |
| aataagtgttctcaatgataagcttagcagagtaaatgaatcgttggataaaaaactgtc |
| cacacggaatgagtatgacagaacgatcgcagagtctgaggcagcttatatgaagattct |
| ggagagttctcagactcttttaactgtcctaaagagggaaggtcaaaatattgtccaaaa |
| agcaacggcgaaaaatgttccgttttagaaagagaattctttagtcatataaacttgcaa |
| tatgtcaattatttgtaaatattgtattatttactactgaagctcctcatatggattttg |
| ttaccggtcgatatttcaaactttgaataatactttcaggtcaaattatagtcattttaa |
| taattttcagctttggttttaataattaaaggtggattctatgcttattgaaaaa |
| >TCONS_00004838 gene=XLOC_002919 |
| cactatcaagatgctacatttggaaaaggaaatagatgaagcaaccttccaaaagtttct |
| gctatttaaaaccacttctagtgattacggaaagtttgcaccaaacgtccacactatgcc |
| aaatgtctactttccattgaaaggtgatttttctcagcatttaggaaaatgtggtatgta |
| tcgcaatcactcacttaacacatcaatgaagaagtaaagctttactagtcatctgatcaa |
| ttccgttaattacatattttctcaaaaatgatatcacgtaatttttatttccatgagttc |
| agtcttataaa |
| >TCONS_00006314 gene=XLOC_003903 |
| tccggtttagttggtcttggcgacatgtcgggaggtagcagtgtgcctgattcggcgttc |
| actggttggaaatactattttaattcttacaccttgaaaggccgttttaatatagtaatg |
| gccaattatgcaatcttgttcgcgggcatcgcaatttggcgaatgaggtcaaagaagaaa |
| aaagccctaaaaaaagatgagacctgatgatattttggagtagaatagaaggcttctata |
| aattagcattcgtgtaaccagttgcgtcttgtcctactgaaatagagattttttagaaat |
| acaattggtgttctcaatgattcagggactgatcttttt |
| >TCONS_00006315 gene=XLOC_003903 |
| tccggtttagttggtcttggcgacatgtcgggaggtagcagtgtgcctgattcggcgttc |
| actggttggaaatactattttaattcttacaccttgaaaggccgttttaattaatggcca |
| attatgcaatcttgttcgcgggcatcgcaatttggcgaatgaggtcaaagaagaaaaaag |
| ccctaaaaaaagatgagacctgatgatattttggagtagaatagaaggcttctataaatt |
| agcattcgtgtaaccagttgcgtcttgtcctactgaaatagagattttttagaaatacaa |
| ttggtgttctcaatgattcagggactgatcttttt |
| >TCONS_00006947 gene=XLOC_004232 |
| ttgacttacgtgatctaattctgggttcgttttgtcattgtatcgataggatgtgttatt |
| gttcattttgatcatttcagctgcattggaaaaggtattgtcgaacgaacctttgggcga |
| gatggagcccacaaccaagaggcaatgttttgatgatgtattagatttaagtcggaagtc |
| taaccatgttactaatggaaatgacaagctctccagcaggaatgatgatgatgtcattat |
| gctgagtgaggatgaggatgatcgtctacttgggtctctctgcgatgaaatggaaaaaga |
| aaacgctcccaagcaatcacttctatccaagtcgcgtgcatttcttttcaaactggaaag |
| tgaacttagaaacgaagaaagtgcattgctgttgcttcagcagttacgagccaatcagcg |
| ttcgcatgcgttacagccgaaaagtactaaagtctcgtcaagtgtaacaactacagtgcg |
| tagtacacaatcacctgttaaccaaggtacatgcttattatttgttcatttcacttagtt |
| cggccggctgtcagtcaagctgtgaccagaaatgtcccgtccacagctggtagtcgagca |
| acgcaatctccagctcagaacactacggcggtgaatccaacaaacgcgacgccaataact |
| aaagtaacgaaaggaatggctatccatgctctcga |
| >TCONS_00006948 gene=XLOC_004232 |
| atttgacttacgtgatctaattctggctgcattggaaaaggtattgtcgaacgaaccttt |
| gggcgagatggagcccacaaccaagaggcaatgttttgatgatgtattagatttaagtcg |
| gaagtctaaccatgttactaatggaaatgacaagctctccagcaggaatgatgatgatgt |
| cattatgctgagtgaggatgaggatgatcgtctacttgggtctctctgcgatgaaatgga |
| aaaagaaaacgctcccaagcaatcacttctatccaagtcgcgtgcatttcttttcaaact |
| ggaaagtgaacttagaaacgaagaaagtgcattgctgttgcttcagcagttacgagccaa |
| tcagcgttcgcatgcgttacagccgaaaagtactaaagtctcgtcaagtgtaacaactac |
| agtgcgtagtacacaatcacctgttaaccaaggtacatgcttattatttgttcatttcac |
| ttagttcggccggctgtcagtcaagctgtgaccagaaatgtcccgtccacagctggtagt |
| cgagcaacgcaatctccagctcagaacactacggcggtgaatccaacaaacgcgacgcca |
| ataactaaagtaacgaaaggaatggctatccatgctctcga |
| >TCONS_00006328 gene=XLOC_003909 |
| tcatttgtacattataggacttcgatccattgaacacggataagagttctgaacaatctc |
| gctttattaacccattctcatgttcacgttgtggtacggacttcacgcctgtttggaaac |
| gcaaacgcccaggttcatctgaagttgtttgtgaagcctgtatagttgacagtcaacgtt |
| cagtcattcataaagcatacagtagtgttatttcagctgcacttaaacaacacgctgctt |
| cagaacgtgaaatcgagcatgaatatcaagacgtagtcaattcaccagctaagttggaag |
| cttttattaaagaacatgaacgtaagcttctggttactcaacaagctcaagtggcacaac |
| aacagcagcaacaaatgcagcaagttgcttcagctactggtttacataatcaaaggtacc |
| accaacaacagcaagggttccagtctcagaatgtatttaataatgctagtagctcaaggc |
| atggacaaacaaatgtggttaacagcgcttcttcatctcagttaggaatgccaaatgttt |
| cgtctcgtcgacaagctgcatctaataacgtcagtgtcaattctcctgtttccctaccta |
| ttcagcagcagggacatgcagcaggaaatgttgttcagcagttggcatctcgctatcaac |
| aacttacaaaagcggctgttgctgcggttgttggtggtggtcaaagtccagttaatcatt |
| ctgttgctaataattctgccactgcaaacttgatgatgactgcagccacaaatcctatgc |
| ttgcagcagcggcagcagtttcggcaactaatcagcaacagcaatcggtcgcccaacaac |
| aattaactgctgcagcattggcacaacaaaggctagctgcagtagcagctctaaacttcc |
| agcatcaacaaccacaccacacacaaccttcagctggagcagtaacacctaattcaccta |
| ttgatcacttagctcagtttactcagatgcagacattgttaatgagcagtctcttgggaa |
| atgcagctgctgctactggggcgcaagcaacagctaatactagacaacaacaacagcaaa |
| tattgcagcatgcacttttacagtacacctacttactacaacagcaacaggctgctgcag |
| ctgctcaagctcaacaacaacaaaatcagcaagttaatgctgccgcactgcaacagctaa |
| tgagcggtgctgcagctaacccggcagcttgtatgacgttgttacaaaatttgtgggcac |
| taaatgctgcgtcaaacggtgttaagaagtagtactttgatgccacaactttattcctaa |
| tctaatgacttcactgttttgtgtgtctgtttgctattgatatctctaaatttttatgat |
| ttgactaacacacgtttcagtttgctgtgccttagtgactacaacgcgtacagcgtactt |
| cgacata |
| >TCONS_00006986 gene=XLOC_004254 |
| ttacctctccacataactaaaatcccctttaggaggtataattgcctaaatattactcca |
| tacagcctcataaagagtttattccacgaggggttattacgacgtccgacaacataagta |
| atatcccagaagtttgcttgccagatttcgcaaatatgcttcacctcataagatattcag |
| agctgtagttttttgtagtgcctagcattctctcattttatttacaagaagcagttcaca |
| tcatgcaaatatgtacttgcttaatactataacattttagtcgttctgaatttctttagt |
| cacgattatttcgtcctgaattaatatactgattttgctcaggtgccacctagcttcgtc |
| gtatcgagggtttggattaagtcggggttttggtctaactctccaactaaaaaagcatgc |
| acaaagtgttatacaattattaaatcagaagcttgtgagagaccgagatctttgtagtcc |
| tgaaagtaaggcaacgctaatatattatgcttgtggtagtagtatctttgtttcgttcaa |
| atcctagtcatgctggcttaaaatttagtgatttacatcataaattactaagcagacttt |
| agagtcagcgttgataactattggtcagttgtttcacctctcaaaatatcgacgttattg |
| gcgttccagtcactggggctatactggcgtaatatgtcagatgtctagtatgtcaagatc |
| cttataaatagcaattgctgaaccaattcagctgcagaaatatctcttcggacagttcga |
| aaatcgacttaaacaaagcaagtacctgtgagcgttaccatcaataaaatgtatttttgt |
| ggattgttatcttgttccagtttaaatatcacgaatgtgctattcgattgctaagcctgc |
| agggttaaacctagtgtttcttcaaagaatatcccttaccatctagaacggaaattttgt |
| gtaatttcagtatatgcagtgaagttttatatgttgcttttacactcctcaacaagcaaa |
| taactacccaatggtgcttgcattacaatcatat |
| >TCONS_00007067 gene=XLOC_004293 |
| gtaaagcaacttttcaaatatagaaaaaagaaatatcgtaaatgaattgtattttcatga |
| atctaccaaaatgtatgccccatttaatgtcatataaacaaaagtgtaacgctgtttatc |
| atctgctgaccacttcctccctattattattatcaccatcaccgttatcatcgaagtctg |
| atgaaataaacaaatcaaatggttataaagcattaatgaaatcgtcatcagttcgatttc |
| gattagataatctaccaacacatgtacaacaatattttaatcttactcctgataattcat |
| ctgaatttattactgtattatcaagtgatgtacaaaagctgattaagaaaactcattgtt |
| cagttaacgttattagtgataatttacaacctccattacttcaactggcttctaaaggtg |
| atacatcaaatcccaacaagtcgttatgtaaatatgggattaatttgcctgggaaaactc |
| gagtaaaagtgattgaatttaattctaatatagacgataatatgtgtagagtccgagtga |
| aacaggcagaagcttttttacaagctgatcattttgtagtcataattatcaatttgaaac |
| atctgaggaaattattgtctaagaaaaatatacagtctacggctacgaaggaggatggcg |
| atgaacagcagttgcaaaatttggggaaggtggaatacgagaaaaatgttgcgaagtaca |
| ctaaaatatttgaaggaatacctcattgtaaagtacacaatattaatcgacacaacgtgt |
| ttagtgttgccctgactttagaacgtacaaaa |
| >TCONS_00007131 gene=XLOC_004328 |
| cattttacaaattattttctaattataaagtaatcaagattgttatctattgctaaatga |
| tagaaaaaatgataacatgtttcatccgttcacattcatgatggtatatatctcctattg |
| aattactgtctatgctagaaacattcgttgaattgaaaaaagaagtaaagaccgagcaca |
| agagagatatgtagaacgaaatactcttgttttctggacctgacattcatcatcactgat |
| gagacaatatatatcttaccttcataaatcagttcgttctttcttctcgttttactgtca |
| taaatctttgtaaaatagactattacttgtgtcaatcttaatcgcagcaataattaacaa |
| tttttattatctttgttattgttatttaacattgaaagatatctattgttgccgtttttt |
| gctgggggatggaaaagattcttccattcatttctaacattcagtttatcgtgcagttct |
| attgatttgttattatattttgctgacattgcttcagaactatttttttaaactcttgtt |
| taatgccaatttgtttttttggatatctcataggcatttactatcatcatcaccgtcata |
| ataataattatatcatcatttatttatttaacacagcgtttctgtttgtttatttgtcac |
| attgtaatttctcttttttgttttatctctgtgagctgaaatctatttgaacaatgtagt |
| atcaatggtaattatatatttgtaatttttatcccaggtaattccccttatacaccatgt |
| aaagacatcgttccttttccctattttctt |
| >TCONS_00006560 gene=XLOC_004020 |
| tttggttggtaggactttatgtgacgtaatttatgatgaggtggattccatatgaagaga |
| atataaaatgggctgtggttgttaatccgttccggagtgatacccgcgataaacttgata |
| tttcaattggtgatgacgtatttattttacgacagtgtgaggattggttttatggattta |
| aaatggacgtttgttcgaagtttggtgtgtttcctaaggcatgtgttgcatttaaacgag |
| aagatctatctgatggggtcagtgctgagttacagatgatggtagaaaaactaatatcca |
| tgtttgtggaaaatatggcagaatgagta |
| >TCONS_00006561 gene=XLOC_004020 |
| tttggttggtaggactttatgtgacgtaatttatgatgaggtggattccatatgaagaga |
| atataaaatgggctgtggttgttaatccgttccggagtgatacccgcgataaacttgata |
| tttcaattggtgatgacgtatttattttacgacagtgtgaggattggttttatggattta |
| aaatggacgtttgttcgaagtttggtgtgtttcctaaggcatgtgttgcatttaaacgag |
| aagatctatctgatggggtcagtgctgagttacagatgatggtagaaaaactaatatcca |
| tgtttgtgtcaggaaaatatggcagaatgagtaaacactgtatggctctccgtgatgttg |
| ttagtttgaagtactccctttcgggaagaattacaaagttcgacattattgctagtctaa |
| ggaagcttcatactcatatgtcgatggtgcaaagtaaactatttttgccattcatcattc |
| gggatgagaattttgaatatatcaatccgtactccatatctcccatagacttacactgga |
| agcacaaaaagctcataaaagaacttgaagattcagaactttctgctcaatgccctgcgt |
| atcatcctttcaatctgcgtgttcgttttaaaaatttacctaaagcacttctgacaatgt |
| acttagtgagtcaagtcttacaaagttctcagaatgtagtcagttcaccagctaatttaa |
| ttccaaatgtggatatgaattttgctgtcaatgc |
| >TCONS_00007250 gene=XLOC_004389 |
| gataatgattctacaattcattgtgaaaaatctgaaactttgaaatcaactatgatgaaa |
| agcgaagagttggaaccaacaaataaagaacatgttaattcacaaagcgccacgatgatt |
| gataaggaagttacattgagcaaacgtggaaaatgttccatatccatttcacgagatact |
| gatccaaatcaaacgtataagtgcggttctatcatattggttggttcatcggaaaccata |
| aagccgaatccag |
| >TCONS_00007851 gene=XLOC_004882 |
| ttatttagacaacttaatgagaatttaacttcaaccaacgacttagagtctatttcaaag |
| acaattgcaaacgataaaaatggtggtgtttcacctcattccgatattaaattaaacatt |
| cagcaagtgttaaatgaagaaaaatctattcagacagggacagggcttacaacaagtgcg |
| tcgacttttaccagtggacgaccatgtttagctggtactcaaacactgaagcacctgtta |
| actaaaaatcaggaaactaatctagacattacaaatacaagtattcttaatatgaatgca |
| tgtgactttacacaagactctagtggactagggtctatgatttctgaacattgtaacacg |
| agttatttcaaggacggagaccggtctgactcgcatttacactggcctaatcacagtctt |
| ataactttagatgaggatgcaagtgatatgagtttggataagagtgatgattatgcaaac |
| gggccatctgataataacagagaagccccaatcattaagacagaatatccacatcttgaa |
| tctcaaataaaatctcccgagttggctatgcgaacttcaccatctagcgaaggaaaatca |
| gccattcaatatggttctggtgatgaatttgactatgacgcaatggttgctcaaacacga |
| atgatcgctaatcagaattttcaaacaatggacgagaatttcgcatctgtggcacgttac |
| gttgaagagctgacaggctgtagagagttgaaaaaaacaactaacacgaaggacagccag |
| attacaaagatttcgtccaatagta |
| >TCONS_00008158 gene=XLOC_005077 |
| ttacagttcactcttgtcttatacaattgtggggacactactctttctttttgtaacaat |
| aatatgcatatgttgccttcgaagaaagtgtaaaaagaaggctgtaaaaatgtacagcgt |
| tatcgaatctggtgataactctgaggacgaaatgatgtgaggcaactcgtgactaatatt |
| ttgggtttaaacgtattctaacgcaagtactgacgacaaactagatgaacattcggtttt |
| accaaatcaataatgatatataactagggtgatgtatcctcaatattttactgttggttc |
| gttcgctaataaacttgattaaaacatttgtcacgtatttttt |
| >TCONS_00008839 gene=XLOC_005432 |
| tataccaatggaatggtagtaaatcaggcaaatatgaacgatttaaaagtggagaatatt |
| tattaagacttaaaggtgaacgtagtggaagatgtcaaattcaagtaatagaagaaaatg |
| aaagctcgcaggaactaggagaatttttatccaagctgccagataccgaaattactgaac |
| ctcctaaatttgaccgtggaacgaaagcagttcatcgcctttcagatgaaagtggggaaa |
| ttaaattatcattgatatgtaaagatgtgcttccacgatctgtgatttctcaagatgatg |
| tttacttcattgacaatgggtctcatttatacgtctatattggagatcagtgttcaaatc |
| aggaaaagcaaaatgctttatcgaatgctca |
| >TCONS_00008840 gene=XLOC_005433 |
| atggctttattcggttcagatcaagaaaggtttgtcaaaaaggaaagtgctgaaactgaa |
| aaagcatggcaacaaataagatctatcagacaatcaactcttttggtttggagaataaat |
| aaatttaatctagaagtcgtaaaaccagatgattttggaaccttttattctggagattca |
| tatattatcttgaatatagagaaagttggcaatgattatgactatgatgttcatttctgg |
| ataggaaagaaaagtactcaagatgaatatgtgactgcagcttacaaaactgtagagttg |
| gatacatttctagacgacagagctgttcagcatcgtgaagtcgatggattggagtcgaac |
| cagtttaaaatgtattttaaaaggtttaaaacacttgaaggtggttatgaaagtggattt |
| aatcgtgcaaaatcgaatgaatttaaaacaagacttttacactttcgagatattgattct |
| tca |
| >TCONS_00008888 gene=XLOC_005459 |
| tgttcttctaaatataatggttatcttggggtgattgcagtaacgccggctacataaatc |
| cttctgggatcatgtcatttaatcctcaaaaaataaatcagacgataaaatgtatcaacc |
| tgaaacgtagcaagactttcagcccgtcacctgttcctgataatttggattctgcttcgc |
| ccaccgttcaacagtgcatttcgtcttactactcacctatacactgttttcgaattcatg |
| atgagcaaaggtttcataagaaatacgcttgtttagccccctcgaatttggactttcttg |
| aaagtaaaagcataactcatgagaaacaaccgaataacataacaaaagaaatttcaaaaa |
| gtgacacctcccaaataaaagattataatgtttttgggtattcaggcagtcccgtaataa |
| ctctaaccaagtcactgccaggtata |
| >TCONS_00008899 gene=XLOC_005464 |
| atccctttcaagtctgtaagatttccgaagctttaacgtggtttgttaaatggtccaatg |
| tgtgttgatttactaacaccaccgtttattttacggcattccattcagatataatctcac |
| gtattgattttcaggtttgtttgctccgagtcatttggcttaccgactggaaaatcaaga |
| ccaaccaagtttggcagaaatgactgaagctgctatcaaagtactttcgcgcaatcctaa |
| aggatttcttttgttagtcgaagggggcagaatcgatcatgggaatcacgaaaatcgagc |
| acaatacgcgttaactgaaacgttggagttcgaaaaagccgtcgagaaagcattgtcact |
| tgttgatcaacaagaaacgttactattagtaacagcggaccattctcatgcatacggagt |
| tgtcggctatccgacaagaaatacaagtgtgctagatgtggataatactgcaaaaggaga |
| tgataataaatcatatctcatatcctcctttttcaatggaccaagaggtatattaggtgc |
| acctcgatcggatccggcaacagaagatagattcgcaagcaactatacagcagaatcact |
| ggtgagactcagtacttctacacacagtgctgaggatgtaccgatttatgctagtggacc |
| tttcagtgatctatttcactcttcattggataatacgtttattgcacatgcaactatgta |
| ctcgctttgtattggaccatacacgaatcagtcacactgtgacaaaggaaacaatggatc |
| tcgactcagaggactttatctgaataacttttattatttactatttctcagtgttatctt |
| gtacactttcataca |
| >TCONS_00009006 gene=XLOC_005519 |
| ttgctatattttggtgtggattataaatttatatgttgtttcgtcgttatcttgttatca |
| atgcaattcatcgcatcattctaattgcttagaacacttaactgatttaggcaaacattc |
| actacaaccaaccccttgtacaacttatggagcacgtttttgtatcaaaactactggaat |
| ctacggagctgtcatgggtattacacgattttgtagtgcatgggatatgggtaatgaatg |
| tcaatttttagattttcctgatcatgatcgtat |
| >TCONS_00009010 gene=XLOC_005522 |
| ctctgtcttgactaacatagtaaagattaaagagaaagtataatcttaataacgatgatg |
| atcatgatgatgatgagatcgttcaacttggtgaagatgatctctttgttattattcagt |
| aatctattaatcatttctatacaaggagtcgttataccagaggctaatccaattgattat |
| caatcaaaacgttggactgattttaaacgagctatcagatggtcagaatatccattcaca |
| tttacaccagagattaaacgtcattatttatatgaacaacgaccatattataatgatatt |
| ttagtataaataatcaatctatattgattgatg |
| >TCONS_00009061 gene=XLOC_005551 |
| cttattgcgcgtcggattagaaatttgcgcttcacggtccttaccaacatcctcctgatg |
| tttcagtttcctccagagtatagtagagtatcagccgttgaaatagaaaggatggcagtt |
| caacacttccgcagagaacttgccagtttcgcaatctcggtcgcagctattttcatagtg |
| ggtttggttttcacggacaaatagttctct |
| >TCONS_00009100 gene=XLOC_005569 |
| gccgcaaaatttcaaaccccagttttgtaacgttagcaacacagtgaggtagagacgact |
| tttgcactaaaatcactgattaattggtcggttgaattaatacattggactgagaagtta |
| cagttcgttggaggccaattgagccgtgaatctatacgcatctcatcggggggattgact |
| ccaacgacaattgcagacaagtgatcgagttacacactacagtttatttatcctctggac |
| taaacttctcacctgctgatgtcgtgttcacagattgcagtagagtgcactagagaacga |
| tttaggcttgtatatccccatcctaacgttctttatcagtggagggcaatacatatgttt |
| tagtcgaggaaatgcatttgcaatatatcgatgtatttttgcttccttgtttgcaactct |
| actcattttggattatatgtggataattctacgtgatccaaaatttccagactttctatc |
| ctggtgtttggatgcatctcagtgggctttgttaagcgcaactgtatgttatataatatt |
| ggcgtgtaatgtggcgtgcatatctagacgaaatgaggaatcaaacaacgaaagtaagta |
| ttaccaatcaataaagctaaactatgagggcgatccagtgattattatgcaaaccaatt |
| >TCONS_00008611 gene=XLOC_005307 |
| ttgtccagaagttctcaattcctttagatacagttactgaatatatgaaatgcatgaaat |
| ttttgatgaaacaggtgtcaagttctgaattttcaacaaaagatataacagtccgagtat |
| cttctactcttcgaaggtgcttcaacagtggaagtttcagtttatacgagttaacagaaa |
| taagatgtgaaataaatggactggaagaagaagaagaggtttcccagataattgcattga |
| ttttcaaaaaagacgagattacaagaaattacaataaacatttatggatgcatcgattac |
| taacaaatattgccaatgaagataatcgtcgaaacacataagtgtgaattgtgagtcaaa |
| taaatttatttcatcgactatattctgttgtgtaaatatcc |
| >TCONS_00009653 gene=XLOC_006012 |
| cttgctgaccagatacgtataaacaacactggacagatcactaccatcgcactactacaa |
| atgaaattcttaacatatgttaacatatgtatatacatattttgctacttgtttcaggtg |
| atatactcaattttccgtcctatgataaagtatatttctcgattagtaacagggagatgt |
| gaactaagtcgcataattgccagttatcctaaaggagcacccaggactctcagaatagaa |
| aactcgctgaggaattcgaaaaataaatctattcaaaatggattgcttttggggaaatgt |
| acagatgccaagtcacatgtgcgacttgtcattagtgctaagcatatcgatctaaatgat |
| caaccaaattttcctaccgattatttgtactgcatcaatcaaatcaactcttattgggaa |
| ctaatagacaaattggatgcctcgagatgtactgcgttcaattctgatgatcattaccac |
| tccgaattactttcaagg |
| >TCONS_00010035 gene=XLOC_006228 |
| ctacatgctgttggccgttacatattcaattggggaacatggacaaggctgtcgtccaga |
| ccaagcttgcaagggtgaaagcggttgttggcagaactggttctcagggtcaatgtacac |
| aggtccgtgtagagtttctggatgatactaataggtcaatcataagaaacgtgaaaggtc |
| ctattcgtgaaggcgatattttaaccttgttggaaacggaaagagaagccagaagattac |
| gttaatgca |
| >TCONS_00009849 gene=XLOC_006129 |
| tcttgagtttaggccttacgcgcctgctttgttttcgtatccttcgatgcatattttacg |
| tcctgtatttcatgccttctgtcgttataacctatagtatacgcaaacctcaaagttcgt |
| tttacctagaaatcagatatgcacatcaagaaaagacttcgccgctacataagtaacatt |
| ataagctttactaattcccacactcgaaacacgcttcgtgatcgatcttctgatttttat |
| gcaaatgaagttacagatgaattggttgatctcatgcgtaaagtttggctaccgattgtt |
| ggtcctggttatgacatcatagttttacatattcataaatctcgaggaacatcaaatttg |
| ggcatcagcatagaaggtgtgacctatgtggcagaaccagatttcgatcatgaaaattta |
| ccatctacattggttacaactaatagtcaaattgaagataaatctaatccaagtagtaaa |
| ctgttgccaaatggtatggcaaaatctgcccattcaaatgatatcaagtatagtgatata |
| ccttcacgccatttcgttcaatatattgtacctgatggtctgatcggtagtttgggtgtt |
| gtgcaaaagggtgatgagttacttcaggctaatggtcatcgtatacatggtacaacgcat |
| actagtactcttcgttatttacgcaatttaccatcacgcattgaattagtctttgcccgt |
| agaaagtctacatatgagaatggagatgatgacgtgtttagtattacgggtggtaaggat |
| cagttgattgatgtcgtaggggaatcactacttgaagttgctgctagtgaaatcggttcc |
| gtagatactggctcatctgtgagaatggtggatgcttacgataaagttgatcggtcaaat |
| tattccagtccagtatcacctgctacggctcataaacgtgtcactgaatggattcgaaaa |
| tctcaaggagatttaagcgcgaatattcactatccatcttcacctgaatcttctgtaact |
| atgcaaactaataataaatcagaaagatttagttgtgatttaaaacatgcacaatcgtac |
| aaaattcaccccctccataactatataaatccgaattacactagtaaattgcaacaacag |
| tatctagtaaatacaatgaaaaacgatcgtaaatattcattaccaagtacagtacagccg |
| tcagaaacacaaatccattctaggactttagggcgtttgccaatatcaagatgtagtaaa |
| gtgtgtataggaaaacaatcatctactgaacaacacaatcatcgccgttgtcaaactcta |
| ccacattatcatcataatcgaccaaatcgtattgatcctcgttacggctttaaacgacca |
| tgttggtcttcagtgcctttaattattcaactgaataaaacatctcatggttttggattt |
| agtatagctgaatatgaggaactacctgttactgacttagaaggcaatacactccgtaaa |
| gcttattcacttgatagaaggagttctaatatgattgaatctgatcgtcgatcatttcat |
| tcctattattctaccggttcgaccattgcatcattatcatcgttacctggtaaaaattcc |
| aaatcttccagtggattaaaacgaagatccacgtggtctagtcgatccaaagcacacggt |
| atcttattagttgacagtttaactccaggtggtattgcacagttggatggacgtatatca |
| atcggtgacaggcttttatttgttaatgataaaaatttaatgaaatctagtgtatttgag |
| gccgctaatacactaaagtcccttcctaatggaccgtgtttaattggtattgcaaaaatg |
| cagttagaatcaaacgaaacagatgaaattcacgaaaaaaaccagcaacaattattactg |
| ccataccctgttgtctctcctagaccttctgttattggaatgtttccagatactggtgaa |
| atgaaaaagatcagtgttaagtctgatctatcatatccgctacacgtaaacattggtgac |
| agcactatcgataatggtgataataatacgattatggtaagtattataacatggtgtt |
| >TCONS_00009851 gene=XLOC_006130 |
| tcagacggttttggtattttcatcgttaatttgagtccaaacaatgaacctggtgtattc |
| gttagcgaaatccgtccaaacagcccagcatcacaacaaggagttcttagacctcatgat |
| cgtatactagctattgatggccaacttcaatgtgattatgaaagtacactggaattgttg |
| caaaaatcaagaaaatctgtacgtctaaccatcggtagacaaataccttatcacagtgac |
| tcacaacaaattcaacaaattggacatgtaactacaacagtggataatccagacaatgat |
| ataaaacataaattacccattatacctgggattcctgctacagttactctgtacaagact |
| gacggaggtcttggattctccattgtcggtggaagtgacacagttttgagtaatattttg |
| gtccatgaagtacactctggtggagctgctgcacgtgatggtcggcttcaagttggtgac |
| cgtttattagctgtcaatggaatcgatcttcgtgaagctactcagaaagatgctacaaag |
| attatacgaacagctgatgactgtatccaattggttgtgtacagagatcctgaaccacaa |
| tatataaatcaaggtgtattcgagtgtcatagtgttcacttgaaacgtgatatgcccgga |
| caaagttttggtctgtcattgattggtcgacctcattattcaaccggcacggctattggc |
| ggcattatagagaatagtccagctgctcgttcaaatcttttagaagttggtgatataatt |
| ttagaaattaatggttgggatatgcgtttggctaaatcagatgaagtagtcaatttatta |
| aagaatgcacataacgatgtgaaattattgattgg |
| >TCONS_00009852 gene=XLOC_006131 |
| aggaatatgcacttgggttcttaataacgccacattaaggatatctcaattacaagttac |
| tctttttgttcttttattgatttgatataatctgcacatatgttgctagattatagttta |
| tcacacttagaaatttgtcatttattacatgaacctcatttttacgtctagttttttcag |
| tgttatgatttgttttctgctttttgatgctttaatttattcattcaaaagtagtgagta |
| aattccaacaaaatggggaagttgggcatcctaattcagatgatgaggtacatccaaatt |
| catcagcattatataaccacagctatgaattgtctcttgagccaacccatgatgttatac |
| atcatttgccgaataaaaatctgtttgtagatgatacaatgcacgtgatatgtgaagatg |
| ctagtactactaccaacaaaaataatcggaaacactcattatgtgttgaattggtatcat |
| cactttcagcaccgccacctgaagatgatggcgttatgtgcaatcgtccattgatgaata |
| taaatcgtaaattttctattcagaataatcaacatacctatgataacaatgggattatga |
| ataacaaagcatgcctttagcaccaacaaatatacaatcatatatccaaacaaacaggct |
| cgtataaacataaatagaatgaatacaatgatttgtgttcggtttctccctcccctattt |
| cttcttgtaatattttattataaacacttgatttgttcgtttcaaattgttaatatcttg |
| atttatttttctgattgagatttgtgtcaagaacaatactgttattatctctgtgttttt |
| tggatgacatcagagtgatacttgtttttcttgtaatgtgtttcctctattttttctgaa |
| taaaaaaccgttaattattattttcaagatgaaaaatattttcccttatcatcaattacc |
| ctctaccgaaaaaccacacaggtagttcagccaagtagtagtaactgttgttggtgcaaa |
| tgggactaggaatttcaaaacaatttatcctctctgtaattcaattgcgaagtgcaagtg |
| attcgtctttgaaaaatattaaaattatcagtttcattcctttattcaactacaccttgt |
| gtctaccataattaaaa |
| >TCONS_00010132 gene=XLOC_006275 |
| gtcaccagacggtgtcggtttggcgtgttaacttactgttcgtgcttgttcataatcatc |
| tggtcactctgtgttgcaagatcagttttcattaaaccaaacaccattagttggcgggat |
| atgaagttcaacacttgttttgttttggggggaccgggtgctgggaaaggtacagtatgc |
| cagcaaattgtgcag |
| >TCONS_00010181 gene=XLOC_006304 |
| acccactgacacaaaaatcttccttcctaccgttgactctaaagttttcatccgaccttc |
| ttttgaggtggcagcaaaacgttccatcattgactgaaccgaaatggtttgctcgtattt |
| ttttaaacatcttcgaaatttaccatcgtggaatgttctctttggtgcatttttgaattc |
| agtaaaagtttgtgtacctcacaagttgttgattatcattccgtagattttagaaatact |
| ttatgaaatttgcgagtcaactaactagaatgtgagactcactcaaaccatgccaatgga |
| aggtcgggatccgaacgtttgtactcctgtgtttcttcgctcgcttaaatagagtttcca |
| gagtaacaataagagaacaaatttaaaatgtatatagccaatgtaatgttgttgggccct |
| agccaaatcatctggcagtttggtcaatgaatattgaacagttcatcgatcctcatcaag |
| gtcaagaacaaccaaagcacatcagttaatt |
| >TCONS_00010393 gene=XLOC_006480 |
| tgcacttgacactaaccaggctgatgttaagcaaaatccagaagagattcaagacttgac |
| tgcatccatttcagttatgctgaaaaatatccaggaatcattcaatacaatgagtgaaca |
| gctcctagaaaaaatagatgatttatcaaagagagtagatgatgttgaaaagaatattgg |
| tgaaattatcagtagtcttgatgatgatggcggtaatgaataataataataaacgtc |
| >TCONS_00010836 gene=XLOC_006723 |
| taaggaatatcattccaatgttcatgatttggaagatacttgtattgtgtatgattgttg |
| aattgatttcatcacaacatgaacccgaaactgttgactcaagccatgatactcacaatg |
| tatctattcaaactgactatgattcatcattgaatgatccagttgcaattctacaagaat |
| caatcaatgaacttaaattattgagaaaagatcttagtgatttaatcaataaattagatt |
| atgaatctccatc |
| >TCONS_00010902 gene=XLOC_006766 |
| aggaggctgaaaaagaagaagatgatgcactaataaaggagttactcgcttcaaaatccg |
| agcaagtccattcattaccattggagtttggaggtgaaaattcctctagaccaatcaaga |
| tgccagctttgaaagatttgatgaaagccaataaaaaggaaccagtagaaattgggaaat |
| cttacctaaagcgacaacttcaaggtgttttacgggtgcaaaagaggccagtatcagaaa |
| ctaaaattccaaagcttgatggtgattctgaagcatcagccatgaataacataaatgttt |
| caaattcctcaacgaataattcgttagcaaatgataagtcaaaagatagtaatgttaatc |
| aacaagtgtcatgtaccagtgatgataacaatactagtactcaattgtatcaaccattac |
| ctggaatggtttattcagaccatagtgatagtgattgagagggaaaagacagacaattgt |
| a |
| >TCONS_00010903 gene=XLOC_006766 |
| aatagaaaagtgcattctatctcaatctggtacttttcttctggttgagttttaataatg |
| actgaacgcaaagtcctaaataaatatttccctccagactatgatccttcaaaaatacct |
| cgtcttagaagaggtgaccggcgtaaacagtttaatattagaacaatggctccatttaat |
| atgaggtgacttcatttacagtgttgtcattagcatcaggtgcaatacgtgcaatggtta |
| tatatacaaagctaaaaagttcaattcgcgaatggaaactgccgaaaatgtagattactt |
| gggcctaagacattatcgattttacatccgatgtcctttatgctgtgctgagattatttg |
| gcgtactgatttagaaagtggcgactatgttctagaaagtggggctaaaagaaacttcga |
| agcattaaaaacagcggaagaactagaagctaaacgtcaagctgaagaggaagaagaact |
| tgctaataatccaatgaaattattggaaaaaagaactgatcaaagcaaacaagaaatgga |
| aatggtcgaagtaattgaagacttgaaacagttaaatcaacgtcaagccaccatggaagc |
| agatcatgttcttttgaggcaaatgtggcgagaagaggaagccctcaaggaggctgaaaa |
| agaagaagatgatgcactaataaaggagttactcgcttcaaaatccgagcaagtccattc |
| attaccattggagtttggaggtgaaaattcctctagaccaatcaagatgccaggttcatg |
| acgttttatctaattgttaatatttctctagctttgaaagatttgatgaaagccaataaa |
| aaggaaccagtagaaattgggaaatcttacctaaagcgacaacttcaaggtgttttacgg |
| gtgcaaaagaggccagtatcagaaactaaaattccaaagcttgatggtgattctgaagca |
| tcagccatgaataacataaatgtttcaaattcctcaacgaataattcgttagcaaatgat |
| aagtcaaaagatagtaatgttaatcaacaagtgtcatgtaccagtgatgataacaatact |
| agtactcaattgtatcaaccattacctggaatggtttattcagaccatagtgatagtgat |
| tgagagggaaaagacagacaattgta |
| >TCONS_00010546 gene=XLOC_006561 |
| gacaacgatgagattgttcagcttcctttttgctatcgttttggcattttcatggcaagc |
| aaaccataacattgatgcttgtaaaggaattggtgaaatatgttcaaaaactatttttga |
| tagatgttgcgatggtagtgtatgtaaactacgtggaccattttatggagaatgtgtaga |
| ttgtttaacttcggggaatagatgttggagaaattcagaatgttgttcagggtattgtaa |
| ttggtttacatgtcgagatatataaatattatcgtttgagtgat |
| >TCONS_00010554 gene=XLOC_006566 |
| gggccaggcgttgcgtgacctgcgttcatacagatataaatggatataaacgcagtcctt |
| ttgggcttgtataatatctcctagtttcatactgtgattcatgagctagtgaattggcct |
| tgatatccgataacacatcccatctaataactaggaaaaaaacgggtcatgttgttgtac |
| ccgaacgactgacgttaaactatggtgagccacaacatcatatatgtctggactgatgac |
| aatcctgtgattcagcttaagagaccggatcatatttgtctggactgacgacaatcctgt |
| gattcagcttaagagaccggatatttggctgacctggtgactcccatgtggtcacgccga |
| ggtctgttcgttttttcaacatatgcatttgttttagcgcaacctgtaca |
| >TCONS_00010573 gene=XLOC_006576 |
| cttattcggcgaggaatataacttcatatttttattctgttcattttatcgtgtttgtat |
| gctacaacacacgtactttatgtatttacaggatctattcggatctctacctaatatcta |
| gaaatatatataatcgtgtatgacaaagcactgtactcactattccatatgctttcatta |
| aaatcgtatttggataatataactcacttaatttctggaatcacctgtccaattcgttta |
| cgaaaaaaacacagtctttcgttgttttgtggccttgaacttactcacctacacacggca |
| gtctcacctgtaaactttggcgcgatctcgatatcattttgataccacgagattgccaac |
| aaatgaatcttactacaaccttcaactggcattctatatttggccttctgggtagtctga |
| cctagtaactggcacgtgatttttctgaagtggtgattatagtcagtcgcggctttgcac |
| tatcacatctagtgggcgacaactttgaacacgcctcaactggagaatttcccgaagaag |
| ccaattacgatgatttggcgtttcagcacatttaggcaagcattgacttcggaaataatg |
| cataccaagaagtcgtaacatttttgaggaaccaggtttatatcattgtctgggatggga |
| taatataccttctttctcgaaatgctcttatagagccacgtatgtatagtctcagcttga |
| ggactccagctgattatctcctctcggtggatgtgttgtttgtaaaagtgagagaacgag |
| gagttgacgtccaggcttttactttttggttatcagctgtcgtgggaatgaatccccctg |
| atgttgctcctctgccccatggattagacttcaattgatgaatcacgaagtgactttgaa |
| aatatcatccgcttcgatctgggcgcgcaggcataaccctacaaatgatattcacagtta |
| tttcgttatgttcgtttatgattcacattgagttacttaactgttctttattcccatttt |
| gtgtac |
| >TCONS_00010574 gene=XLOC_006576 |
| cttattcggcgaggaatataacttcatatttttattctgttcattttatcgtgtttgtat |
| gctacaacacacgtactttatgtatttacaggatctattcggatctctacctaatatcta |
| gaaatatatataatcgtgtatgacaaagcactgtactcactattccatatgctttcatta |
| aaatcgtatttggataatataactcacttaatttctggaatcacctgtccaattcgttta |
| cgaaaaaaacacagtctttcgttgttttgtggccttgaacttactcacctacacacggca |
| gtctcacctgtaaactttggcgcgatctcgatatcattttgataccacgagattgccaac |
| aaatgaatcttactacaaccttcaactggcattctatatttggccttctgggtagtctga |
| cctagtaactggcacgtgatttttctgaagtggtgattatagtcagtcgcggctttgcac |
| tatcacatctagtgggcgacaactttgaacacgcctcaactggagaatttcccgaagaag |
| ccaattacggaggtgctgtgaatagtgtttggacagcattgtcaaggacatcactaccta |
| ttattacctcaaagttgaagaaaattcagtctccttaatcaaataaatagaataacatca |
| catcaccaaatatagtaagatgatatgggaaccgggactgcatttgtgaagttggtgaag |
| tgtgaaacgagctagttccgtcgttggaaacaggtaaatactggagtgtttgtatgaact |
| aattatt |
| >TCONS_00010641 gene=XLOC_006622 |
| ttaaggtcttttggaaatgggttacgaagttccattaacagtaatgactgttttctggct |
| gcttgttggctgtggaggacctcttatagtccccaaaggtcctaacagaccgttgattca |
| actgatgcttgcaacctcttcagtattttgctacctcttttggctgatgtcatccatggc |
| tcaagtcaaccctcttttcggaccgatattgcatcgagataccattcgcattctccaacg |
| tgaatgggagccaattagagtgctttaaaattcatcagtaatcatcgcatatcttctgcc |
| tgttgtcaacgtatctcatcacagttctaatgcatttatatgaagctcagttttttcgtg |
| tcaattgtgatcatcatatagttattcctctcataatcatcaatgtaaatgattaatttt |
| tcg |
| >TCONS_00011004 gene=XLOC_006831 |
| ggaaccagctcatatattgatacctgcccagccaaattctaatataaataataataataa |
| ttctatcgcttcatattctattggtccatgtacatatattttatcaatatctgatcagta |
| tttaatatctggtcacttgaatggctggttaattatttggtcaattaaaaaatatcgtcc |
| tttacgtcaatggattgggcataatggatatcaaattacaagtttacatttttggcctcg |
| aaatcataatgataataataatgtgataatatcccacggtcgtgatggctttattcgttt |
| ctggggtttaaacactttaaaatttgatacaactgaagtgtttaaacctcttgatcaaat |
| ttttggtgaaattcatacatacgatatttcgttttgcaattccgatttatggcgtgcatt |
| tactacaacaagtaatagtaacagcaacaattcaacggatgtttatttcttggcccattt |
| gtgtcaag |
| >TCONS_00011021 gene=XLOC_006840 |
| ttcaggatgatcagatctactggtagtttaccggaatcatccacaacttttaataaacca |
| aactgtgacagagacggccaacgcaatacagttaacagtttgaataatgttgttgttgtt |
| acagtaccatcagttaatctgacaaatccatctacatcagtgattgttgttagtcaatcg |
| actactacgatgtctagttctaatcgtaattctcgaaataattttaattacaaatgttca |
| tgcaaagagttagcgtctcatccttctcatcgttccaatcactattcatctggtgttcat |
| tcctcacattttcaacatcatggtaatcatcatcatcatcatcaccatcatcactataat |
| catcctggtcataaagaaaatcacacctctcatagtgttgttgaaagtagtaattcatct |
| cccaccagtgggacaagttcaagtcatcattattcttcacattattttacatctaatcaa |
| actgttccaacttttcaacacaacataaagcataattcattccccattaaatggaaacca |
| gcgataagtagacacttacaagagttcatgatcagtcaatgggaacagtttcatgcacat |
| ctgttacattcgaataatattgttacaaaatcctgctgtaccggagcaacaacaataaat |
| actggcacactgacaacagtagccattaacaatcagcttaccacatctggtgattctact |
| aataataatgacagcccatcatcatcaaatgacatgacttcatcaagaactagtttatct |
| gataattcttcgtctggacaaattgccaacacccaatctaataataatggtagtaataat |
| aatagtggtaataattcaccttcgataattgaatcaaatattattggtcgtagtagcggt |
| agtagagtgaatgatttacctgtcacactagctactattactactactattagtaataca |
| acacatggacccggtagtaataatgtcagtagtaacacttgtggtgttgcgatgagcagt |
| tgtaatagttatcctagacgtaaacaaagttgtggatatgttacaaaccaaatggggaag |
| ggtatgaatacaggttcattgggatggaaaagaaatcaaacttcaggaagaaaaatctct |
| tcacctgggaattttaacaatcgatatcctgtaaattacttcagtaatcgaaactcttca |
| ttcattatatctactactactactactactactactactgctacatcaaatagtgtgctt |
| cctgtcgtcacgtatatttcatcatctataaccaccacaaccatcacaacaacaacaaca |
| actccaacagtagtgaattcatcaaacattgatcaaatgagtatcaccactgtaaccata |
| aataatcaatccaatttgcagagttcaaatgtgattgattttaaaacgaatacttcagta |
| cccttactattgtcatcatcctcagagggtcgtatacaacagaaagtttcttcttctctt |
| cctgaatccaattctatttttaaaattgatatttacaactcttcgactgtaacgaattca |
| ataataaattcacaaaaattggaaataaaaacagaggatgttgataaagtttcagatacg |
| aaaattcatgttaacaacaactgtgttgttatagcacaaaaacatttgtctgatttacac |
| cttaccagtggtgataataacaatacaattgataagttgaaagaagatattccaattgat |
| gaatcgacgaaaaattcatgtattactttatccaattcatcattattattattgcctgaa |
| gaagaaagaacattaaccattgcatcaattttgaaagttgatcaagaaattggaagttct |
| attagctgtggaaaagttgaagaaggtgagtgctactggactccggatcctccagcctct |
| cctgaaaataacactaccccacatcatgaaaattattccagtagttatcattcaactagt |
| gtatctccaagtcctccacaactatcagtttcattaccatgctcaaaagatttgcacact |
| actgcctatcatccaccgcttagtattcgcacagaattatcttcaatgcttgattttcgt |
| cgattcaatcaacccagagattctccgcttatctcatcatctaggttagaaagcctcaat |
| gatgtcgattaaaagtttaataagaatttttttttctattgtctattataatcaccaata |
| tttaattgcctccaggttttctttatttcaaagaactaaaaccctccttacttttatttt |
| >TCONS_00011136 gene=XLOC_006904 |
| ttgctacccacttctcgttatcaccataatatgtcagcgtccttgttccgtggtttgaca |
| aaagccggtgttggcttgttggctgcaagcagtatacttccacttgttctttacaatgtt |
| gatggtgggcaccgcgccgttatatttgaccgatttaaaggtgttaggcccgatgtaaga |
| ggggaaggaacacactttattattccatgggttcaaaagcctattatttttgatattcga |
| tcgcgaccgagaaatgtgcctgtgatgactggttcaaaagg |
| >TCONS_00010764 gene=XLOC_006689 |
| attattcgccttatatccatacaatacatttttacatcattatgtaactgatattattaa |
| atcaatatttaaacattcaggacttgtcaacaacgacaataataataatggtaataactc |
| tactaatgtatcggataaaacaacaactaatcttatcaatactactactactagtaataa |
| tactgttaaactatcttctaatcataaagacaatcaacttacagaatcgttatcctcttc |
| aatattatcagcagcagctgattcgtctactaatactgttccaactgaaccagttaattc |
| taatgtaatccattcaacaaattcatccagtatcaatgaacttgatccagatgatcagtg |
| tataccaccacctattaataaaattaatcaacattgtactgaattcagtaaaaatataat |
| aattaatttaattaaagatcatcatcttattgattggtgcttaaaattatcccctttacc |
| atcattagatgaacgtccgaatgatggggattcaccgaattgtttagttcgactatcaaa |
| agaccctgtaaaatcaggttattcaggacatatctggcaaattggtaacctaattgtatc |
| agcaatgaatggtccgtatggagaatttttaaaatctctcattcaagatttagatccgaa |
| gactcaaaatctatggtcagattttgtcaaagatctcaatgtgatcaactctgtagaagt |
| tgctgagacatctcaatctgccattgaaggtcttaatcattctgatgtcccatttgttct |
| ggcacctgttacatcatcaaatttaatgcaaaataaattattagatctcactgaaaaatc |
| accatcttatttatttgatgatgttgacaataataacatacatgaagaatatgataaaaa |
| taatcatgaatatcttgcttatcatccagtaatatcagcatctacattagttaatcttca |
| acgacaatctattttacaaaattttatggattcatggttagatccagatgaagaatctga |
| acataatgataatgctaatagtaatagtatttatgatcagaataataatcaatctgaaga |
| aaaagatcaatttaaaatatctgatgatatccatattggggttcctggacctttaggaga |
| aatgtattttaatcataatagtgatagcgatgatgattatttcaatagtgatgataataa |
| agatgatgataaagatgagaatgatgaattagacaaaagcaaagacaatattcacagtag |
| aggttactgtaacagtgatgaggacgacgatgatgacgatgatgaagaagatcttaagtc |
| accagttcaaattaaacaacaacattccagtaaacaatcaattattaatgctaatagttc |
| aacatctgtggtgtataatcattcaaatgagttcactgaagtatcttctgtgacaactca |
| taataatgaatct |
| >TCONS_00011454 gene=XLOC_007172 |
| attagagaatcattatttttaatttcataaatgttcacaaatcaatttataatgtgaact |
| gataaatgttatgcgtttgatgaaacccacagtgtacaagagaactgttgatcttcaata |
| atacacatcaacaactgtaagctggaagctggtttgttctcccccacagtaggctattgc |
| tgatggtatccagccaacggacattgagcatcttgtgtagacaactatttataaatactt |
| gtaccttcgtggtgatggttgtagtaattctggaagcttcagctccgtacaatagaacta |
| ttttgacgccgttactgaagataatgattttgatattttctggcagttgttttaagttgc |
| atatattcttcaaatgcaggaattctgagcttactttgccactccttgccttcacatcta |
| catccggtcctccttgttcatcgatgatgcttctcagctatgaaactgatataaaatatc |
| agtgtagaaccc |
| >TCONS_00011501 gene=XLOC_007196 |
| agacaagaagatgaaagaagaacgaccgataattcgaacggaatctataaatgtgacggt |
| ggggatcaaggaggacgcgacaactctcaatacatatatccttataccaatattggttgt |
| ggtagtttttgtggtcagcatagttttgtttcttcttatcaaagtgttctaacaacaact |
| ctgtcgtgtattcgtacatttgtgtgcaa |
| >TCONS_00011522 gene=XLOC_007205 |
| taataatcgtatcaataataatcgtcatttagataaattacatacaagacaattatcaga |
| aattactggagaagaaagtgttggattactttcagttgctggaatgtcaaatttatctaa |
| tccaaatccaattgatatttgtaattctacagttcatggtcaagtctatactactactaa |
| cgctactactacttcgactgctactgttaacactggagctcgatggaataaaatttctac |
| aaatattattaattcttcaaatggaataaattatcatgatggtgatattgattgtagcgg |
| cggtggtggtggcggcgtcggtggtagtggtggcggtagcggtgttggtactgttggata |
| tcagggaaattcttcttcaactattactacacatcatccaactgaa |
| >TCONS_00013192 gene=XLOC_008076 |
| accttaaggattattacataaaattggcgaagatatggatatcactctagatcaaatgga |
| gagatacgtgcctccaatcaatcctgccacttatcccgtactcactctcttgcttttgac |
| catcggggcattctttatggcgtggtttagtgtttatgaattaacagcaaacaaattttc |
| ccgagttttgctgaaagaattgcttctgtcatttgtagcatccatcttccttggctgtgg |
| aacattgtttcttctgttatgggtcggaatctacgtatgagttgtgaagtctact |
| >TCONS_00011874 gene=XLOC_007432 |
| ttctgtcttacagtttctctctctttatatatatatatatacctgttctgctattcactt |
| cagaccatgacaatcatattctatttgctccgtcttttatgtacgtgtatgtgtgtatgt |
| atgtatatgcgagcagtggtttttctagtcattataatattatgttaaaccaatagtcct |
| tcttagatggttgttgttgtcgttcacattatgatcttttacatgctcttctgaattagc |
| actgctgtcgtcttcttgacttactacaccttgtctactcatttcacatcactattcatc |
| attattgaataaattcattattaataatagtaatgacaataataataatcacctcatata |
| cacaaacacacaccatccacagtacgacaactataaataatgttttcgataatcctgttt |
| aattttctgcactacaattcgaagactggctattgatgtttctcgtttagattatcctat |
| cttcttgtgggtgtgtgttggtgtgtttcgtaaatgaattcgcactgctaacacttttat |
| catattgtaacatttatctattgatgacaaatggctttatgatgacgaggatgattactt |
| cgtgtttcatttccgtctttctttttttgtatatccaattatgttctgtattgttcagat |
| atataacccttttttaga |
| >TCONS_00013209 gene=XLOC_008085 |
| acttcttgttggagcagtttgggatcatggttaacagtgatactgtccttcgaacatcat |
| attttaaatattatcatcctaaagaagcattgatcccgaatggagtactaaataggtatt |
| tctcaacgtattttatattgccatacagaggaatagcggaaaccttgggaaaaatgtcca |
| tatcagctacgcaaatggatgtatcacataaacaggcgttacaattcttatatccttatg |
| gagcttctctcaatgttgccaaacctgcagttgctctactgtcgacagggagtgttgcgt |
| ttccacttaatagaccagtttgtgccgtttataaatttccctcacctaacggtgggactt |
| tagctgttgttgggagcgcagccatgttctctgatccatatataaaaaaagaagataatt |
| ttaaaatatttgagtttttattcagatatcttacagaggaatccgtcacgctaaactcca |
| taga |
| >TCONS_00013257 gene=XLOC_008107 |
| atattctccaagcctcatcaatacttcatcatctagttagcagggcccgggagaatggat |
| tagaataacctaccgtgtcactgtgtcttgacctgcattccgtttaccgaataaggtgaa |
| ctcgtgatctcatcaaacatatcaataacaaaccaccaatttccatcaaatacccgaaat |
| gctaaaccaaaggggcgggataaaagctagcttgaaattgaagttcagactttttcattt |
| cagcgaccctgagaggcttgacttagggcagtcattccatgattgaagtgcctcagttca |
| gtgatgcttttaaaggatggctgtaattagtaatatgtgtgccgttggcggagctgcatg |
| tctcagaagacaccaaacagtgccatttattgtacgttatttcctgcctttcagctagag |
| aaaacttatgtgttgagctttttaattttcggcctttggtttgtagtagtggtggcactg |
| tgttaaacacgtctgaaagtattttgacagacctaaccgatacttcttggcaggcgatcc |
| tatctcaaaggttcttgacatgtggtgaattcagagatttcttgaactttttccagcata |
| taaggaaagcataattctgatggttgcaaatgacatgtactcatgacatccacaaacaaa |
| gtaagcactccttttttcctgtgatgtttctcaataacaattatgtggaactttcaggtc |
| cagcacatcatgcttgtagggg |
| >TCONS_00011915 gene=XLOC_007457 |
| acattcacatcatttttctcttgtttcgttcaatctggtagtctttctcgtagtatggta |
| ttagcgggaacaaaaaccaggacaccgctatcaggtatattcagttcagtattgattgta |
| tttgtactattatatttgggcccgtattttgaagcaactccatcatgtattttatctgct |
| ataattgtggtggcattgaaaaatatcttgacacaaccgaagaaactgccctatctatgg |
| aatacttataaaccagatttttttctattcaccgtaacatttttgggaactctcatactt |
| gatgtaacttatggtctgttagttggtctgatctcttgtcttattgtattaacggaaaga |
| cagcgaagcatcaaactactggaattgtgcaatatctcaggcacagaactttatgtccat |
| aaacattatgaagtaagctaaactgttaataatttattttgtattggttgtgtgaatctt |
| cccattgatgcttagggttgcaattgatcagtctcttattggcatatgagcatcctgcgc |
| gggtcgcctcgatattggcttaagtcacgtgcataataaatagggatggacggtggat |
| >TCONS_00013368 gene=XLOC_008161 |
| accttggggatttctctactgttatcagatccctagttacgttttatctaaatccgtgtg |
| gtttgttagattgtatgatgtctcccataagcaagcaatacgaaattgaaatcagaagac |
| gagaagttcgagatttaagaatccattgtgcttgtgaaaggcaaaagttcagccgtactg |
| ttttagacctaattaattattgtcagcaaaatatcttatcagatcctcttattcatcgtg |
| ttaaagataatcctttcaatgagaagaagtggacgtgcgggatgttctaaaacgttggac |
| aggatgagtgtaaggaagcccatt |
| >TCONS_00013369 gene=XLOC_008161 |
| accttggggatttctctactgtattttgacagttttagtaacgatcgtgttatcagatcc |
| ctagttacgttttatctaaatccgtgtggtttgttagattgtatgatgtctcccataagc |
| aagcaatacgaaattgaaatcagaagacgagaagttcgagatttaagaatccattgtgct |
| tgtgaaaggcaaaagttcagccgtactgttttagacctaattaattattgtcagcaaaat |
| atcttatcagatcctcttattcatcgtgttaaagataatcctttcaatgagaagaagtgg |
| acgtgcgggatgttctaaaacgttggacaggatgagtgtaaggaagcccatt |
| >TCONS_00012078 gene=XLOC_007533 |
| agctgttcagtcctccttcagtgataaggttatatcactagtgccttgaagacaggtacg |
| caaatatgtaaccgagtgtacccttaaaataaggtggcaattattttgatgaccgactct |
| tacttacggactcaagatctgtcaagtctattttgtttacactttccaatataaccttca |
| caaacggccttggttgtcagccagcacataagcacacagcttgtaaattccaataagact |
| tgactttaacccaaatattaatataaacagaccctcgtcaaagctgttttgtaagtgatt |
| gagttttttttcgtgattgcctaaaattcatttggaaagaagtattttaggatctcctaa |
| gctcagttccaaactaaacatatactacccatcctctggacaaagaaggcccaatggcat |
| gtttctttatggattttttcccagcctttgtaagtacacctgcgacatactttaaatctt |
| ttatgatctcgtcgcccagataagtactgtcgaaatgccgctaatggaaatgtgcagagc |
| aatacaatgaaatttgatttttcattcatacggctactcagaaatctgtagtatctagct |
| tagcattcagtctcaaaccaatgattttggaaattttttcaactcattttgcgttcaagg |
| ctcgtaacagtaacaattaaaaattagtggtagtaagtatcataatgtacgataggctgt |
| agctgaatatcacatgcttgaatattcacctatttagatccataactgcgactaagatag |
| ctcagtatctgtgttttaggagcgatctcatcttcacaatgatggctggagtccattcat |
| agtattacatctttaatttcgtgaaatccagagtaagacggacacattcctagactcacg |
| cgataaatttaataagcgtacagtttctgtcttaataagattaatatcacaagtttcaga |
| gaaaaatcgataacaaagttgtaagtttgaacatttttgttacatatcactgtccacttt |
| aaagaacacttcctaattaacgtaaattattattaataattttataaatccgttctcgat |
| cggtaatgtcaaatagcctcatctgatgaaaaggtgactcgagttgtgcttctgttatgt |
| ctaggtgagtgcacataaaactaaacgtagctaaaattgccttgtaaacattactagcta |
| agaaattatgactgtcaactcagttccctgtcctagttgataacttgatactagcccatc |
| acttttacctagtgactcagaaaattactttttatgagggatcgccataggttttcttct |
| ctatccataaccgctcagcgtaatttctattaccatgtcatccagctatgtgttcatagg |
| tctgcatacataaattaactcagaaaagactgtcattcataattattcgatatcataatc |
| tgacattttatttcgttattgttacaggttaataacattattaatcggtctgaaatagta |
| aagtcaaatccggttttgctcagaataagaaccttcatggacgcggtaattatgcatagt |
| tcttcgagatctacagtgattttacaaaaatcccacacttataatattctacagtcatac |
| cccaagtttgtgattaaaaacattgctctggggatctgttcagtgagtgcttgtagagat |
| aagttcttttcatggtgagatgatctgtagttgcaatcaaagcaaatgttcctctttgta |
| gaaaaagtattccagttatgtatcatgtaagacttcatgctagatttggactccaaaatg |
| tcatcgaacatggtctcaaccgcgttttcgatagaactaagggatgcattttttaagtgc |
| gttaacaaagttgtgaactggcatcagccattcatttgaacaaacctgagtttgatttca |
| tttcgaggtgtaatttctaaaaatcgaaggtgttgcttcgtctgtaccacctgaaaatac |
| taccaattggttagatgaaaaaagaaaaacttaactatagtaaaggaaacgggctctatt |
| gaatgtgtacataccatttacgcttattgacaacctaatctaaagtgttgttctcgaatt |
| gtcagattttaactgcattagctttggcgtaaccgagttcaattcctagccatatggcac |
| aaaaggtatcaatgactcaaaatgtaaggtttgacatcaatgtccgttagcttcagttgt |
| tgattcatgtaaacataacaagaaagcaagagtagaaataatatacaaaaatttaactta |
| agaatgctgaaaataaattag |
| >TCONS_00012081 gene=XLOC_007533 |
| tccaccggaaatgcaatgacttgcaccgacattatcttcagcctttgtggttcaatacaa |
| tacattcgactataaacacaatcagtttgtttttcttaacaaatgaagacttcttatata |
| ttgtgtattgcagccggtcacgctctttaaatgaagccaaaagacctgcattctgacaac |
| ggctttagtatattcttcaagtatataacattggttgaattatactgttcatcaaaacga |
| gttctcaatctaagaaaaataattatctgcgtctttgatatcaacacgtgtttccatgta |
| cgaatgtgaactaacagttgaaaaagtagggtctgtactgtacgataagaagaatttctc |
| ctaattatatctatccccatctagttgatgaatggagatgaatcacactggtgggtggga |
| ttgcatccagaggaacaaccactgaaatttcctcgtcaaaccatttatggctaatcacct |
| ataaataaaatgtctgatgtgtgcatatccgacatctaccctgaaacggaatcgttaaac |
| agacctcagtaaatctggacgagttctgctatttaactggaagaaccttgctggactata |
| ttctgatgttaaatcagtcagtgtgagccgcctaatcgcagtttcagtatgattctatag |
| ttttcaaatgtatatcgagttgcatgtttcgtaaatatcaattaatcttaatgaacgcct |
| ggctgccgaccttatgtaccatggctcaatggatagaaatttgtccgagacttctagaac |
| ggtgacagaccacgtctctcgttttcgacagagtatttacttttctagaaatgtagttac |
| tttgcttctagaattttctagacaatgttacatcaagtagcactaacacacacgacttcc |
| tgtacataccta |
| >TCONS_00012151 gene=XLOC_007563 |
| tttccaattttactagtaaaacatcacgtaaaactacttatcgaggcaacattttcaaca |
| ataagtgatacttcacaagaaggaaaatcaaaattttaatcgtttgaacgaataataaat |
| atcagaataataaaatatcagtaatacgtgattattactctgtaaataattatgggtaaa |
| attcattttaatacgtctgtacctgattcggatcaaataacaaaaaataaaaatgtagaa |
| ggtgatgggggaccattaaaagtgtccgcgaattcagcaaggaaagcaaagaatcatttg |
| caacgtgttgtttcaatgttttattgctttctaatatttgccattggtttatctattaca |
| ctttacaataccggagaaaaagtagctgaaactgaaaactcatttgtttcattttatacc |
| ataatgtttgtcagctctatcattgttatgatttttacactgggctacgtgtatcaacat |
| cgcaatgctagtttggaagcaatcgaaaaaggagagctatcagctcctagaatagccttc |
| tcatttcgtggcgaagatgtaaacttgtatctgcgtttcggaatcggattgtttgcggct |
| atgtctatcgttcactcagctacaagatgttatgaaattgcgaagaagtatccgttacat |
| gcggcctctatcatttacatatttttcaaaattctgttctttatcactcagactgtat |
| >TCONS_00012152 gene=XLOC_007563 |
| aagacgatgtgtatatttttattcatcagtcctaaaattaaaatgtagaaattttatgaa |
| attagcataaaggagttcatatttcacctaagtaactagtttgtttttctactttttctt |
| acatttttatagtcattttataccataatgtttgtcagctctatcattgttatgattttt |
| acactgggctacgtgtatcaacatcgcaatgctagtttggaagcaatcgaaaaaggagag |
| ctatcagctcctagaatagccttctcatttcgtggcgaagatgtaaacttgtatctgcgt |
| ttcggaatcggattgtttgcggctatgtctatcgttcactcagctacaagatgttatgaa |
| attgcgaagaagtatccgttacatgcggcctctatcatttacatatttttcaaaattctg |
| ttctttatcactcagactgtat |
| >TCONS_00012178 gene=XLOC_007573 |
| tatggaaataaagtaaaagtagaaaaaaagcataaaatattgctggtttgtacatacaca |
| gacacatggatacggtagtacgtaccccaagcatatttttacacataaaatagccttttg |
| tgatatattggaatttgctaaactgaacttcaatattggtcgactacaatggattgataa |
| atctggatatgattggtacccattctttagaagcaatttttcaacatacgaaaatcaaag |
| cattttgaatgattacagttacacaattcgcagaaaattacaggatattcaacgtctccg |
| acttctacaaaaatttgaaaaaggttcaagaaaaacagaagcgttactatgctacttaat |
| agtaatattattatataaaattcttagacagtaatgcatgtctatacgtgtattttttaa |
| aaatggcttttatcgaattcaacggaaaaaaaaaccgttcgtactatctaacgcacaatc |
| tcttagtcactaaacaagctggtcaaatctcactgaagtcgtcaaaactagagatcacaa |
| aacatgtttgcaaaaaaatatttctatattattcaactgaaatctgcttcacctccgttt |
| gcattatatgatgttgcttattcctaaaatgtcagtccatgatgagagatttatttgtac |
| tcaaaatttaatctaaagaaaataatcgcatcattaaaatacctcattatttgttgaacc |
| tttgagatcgtctcactctaaaactctggataagcttagttaaaaagtaagcagatttaa |
| cgaataatcataagggtagaagaaaacttaaactgcacccaacatctttactaaaccatc |
| tactccaacgtaatttaacacacaactaaacatgacgaaatgtgcatgctttatttgaat |
| tatcgcatttgtattcatctacttcactacccgttactgcaaacactcacaaagattgtg |
| ttactctttaatatgttttaggttttcttgttttctcattatttcctcatctaatttgtc |
| ctttcaatcacttcatattagtatttgtgttcgagtttgaaataattttaatcagaaaac |
| cctatgaaaaaccatttgtgtcatcaaaaatatgcttactcattgtgtcatctgttccga |
| agttcctctgaggaatgtatcttttatacctctcattctctcatggaatacgaaaaacag |
| aaagcctcttcattttccttttcatacgaagtgaattttgtcttgaattcatttttattt |
| gcaatttcaactgtccgcttttggatgtttttcatttttggatttcaattcacttgtcta |
| tttttatttccccaactatttacgtaaaatgctttttgtgtttgctttttaaaatttgtt |
| tacttccctaatttttattttttttggaggggtgagatgagaaagaagaagagggggtag |
| aagtagaaaggaagttaatgaagaaaatgatacacacagattatttatatccacttctat |
| gtaagatataaatattaaagtagatgtactttttttattcacatttctgtctaaactcgt |
| acttt |
| >TCONS_00013464 gene=XLOC_008202 |
| gcatactcacgcggcttagtgtctgtcgggatagttggatacaaaatggccatcagacct |
| ttacatcacccaagaataataaaaaaacaccccaagaaatggcatcgtcaccacagtgat |
| ctgtacaaacgacttggggacaaatggaggaaaccaagaggtattgataacagagttcga |
| cgaagattcaaggggcaggtgcgtatgcccaaaattggttatggaagtgccaaacgaact |
| cgtcatatccaccctgatggcttcaagcatgttgtgatccgcaatgtgaaggagctggag |
| gtcctacttatgcaacatcgtactcatgctgcagtaatcgcacatactgtggcaagaaaa |
| aatcgtgtcaaaattgttgagcgtgccaaacaactcagcataagagttgttaacgccaac |
| gctggcttgagatccactgagaatgagtgatcaataaatacaggcttatattttggatat |
| ggatccaaaagaagaatacataaaccatgcaatgaatatggcataaattgatagattggt |
| tgaaaagccttatttgagcctgctagatgactttcatcaatcaag |
| >TCONS_00012271 gene=XLOC_007623 |
| cactagaattgtacactaaaagactcaacgaaatcgttcgtccatcaggggacgttaaag |
| aacaacttttgtcagcaatcaaggacatcgaagcaaaaatagagtatctgaaagaaataa |
| tctttctttcagaaaattgtgagatctctcatttttgtcatgcgtctgaagatctaaaag |
| ttcgtatggaaaagttgtttgagggagtatcaaaaaatcaagaacagatgatgggcctac |
| ttcctaaagtatcccctttgcacgatgtagcatatccagtaatacaagaatgtgaagcct |
| taacaaagcaattgaggatcttggaattcgctgaacacttgtcatttcttgagtaatctc |
| acttcatctatatattatctgtaaggtctggtgtattatctgctaggataattaatgaca |
| aagacactctaattgggtcaattaaacaatatttttccttccttaatactcagttatcga |
| ctgatccggacattttgccctctattttggaacaatc |
| >TCONS_00012289 gene=XLOC_007632 |
| catcacattttcccatatgatctgttccatcaaacctttccataggatttttattcgacc |
| actgaaacaccatcctttgttgggaaaagcgtacagggctgcatacgaccctacacaagt |
| ttaataattgaaacaagtcattaaaggatcgcttcactgcatttataaaccgtctacgat |
| atattcgccgtcaatccaaaaggacatttggatcaatatatctacccaacaaagtgtgaa |
| ataaaagatttgtctgcatcaatgtactcataacaattatgtcgggctcagaagaggaca |
| gtagcatttttgttaaaagactgtatgctcgctataccaaagatgagattataactccac |
| aagctcttgtcacttggtttgttgattatttaaattatccatttatgtatgatgaaaaat |
| gtaagtctaattttggcttcttcaaagatcctaaagatacaaagaaccatccatttcaga |
| ttgtttcttttattactcgtgatgccttacaaagcaacacgactctacggttttcagaaa |
| agacagagctgttttgtttatataattcttaccgatttacttttgtcattgactttagtt |
| ggtcgactttgtgttcaagcgcagatggaacatgttttgtaaacagtattgtaaaaacaa |
| tcaaccaaattttttacatcctgtctgaacccagttcttatagctttcctgggactggtt |
| acaagttgtccaattttacagtattttgttcggtgatcgttgttgttcctaactcaaagt |
| gctacacgttattgtctggttgggttgttaacactaaagaaattcccaataaaatagcat |
| atatttctcgttcacttgtgaaacttgagcgtaagtttttggaagacattaaaaataagc |
| gtgatgactctaaatgtactggcagacgatacgttttcttatcagacctacttagacatg |
| gggctctatctgtgtcattactgacccctacctcaggacctgccacaatcttgtttttta |
| cacacggctgttttgcaacatctgattttggtgttccagaattagtaatgtctcatttaa |
| atgtagaacatgttcgatgtgttttcattttgatgacagacaatattacccctgatttcc |
| acaaaccatatactccccataacacgtctaaaccatcttctttcatgaccgcgggtattc |
| agcaagtagagttgtgtagtgtcttagccaataacaccgatggattcgtcgtcaatatcc |
| cgaatgaatctcaacatttagcggttcagatgaattcatggaataatttggccgaatctt |
| tgttagccgtccccgtatttcgtcgtagcgagtatacagaatgttatagtgattttaacc |
| gtgactcccgag |
| >TCONS_00012295 gene=XLOC_007634 |
| tgccttggagctgttgttaccatttcgttaccaatcaccatgccatattaatcactgtac |
| ctctttacaattgatctagaaaatctcgcatatttttgtcgacatgcctgcgttgatttt |
| cttgggtaggacgtgggcttttgcgtctgatgactttgtttgtactggtgttgttaactt |
| tttattgaggattttgtgtgctttcatcatggttatgcctcttacgttagccagcgaccc |
| aactgtttgtcttccaaatgtaatagacaaggttttctgtgtgcttatgtttcttgtaat |
| gggagctttactggtttttgacacagcgttatcatattcaagcagcaagggtggtgtgat |
| ggaaaccagtttacgttcacctgtccctttttatctcagtgccgtttgcgtattttccct |
| tatatcgtcagtgcttcattgtgttaacttgtatttagttgtcaataggtacacattatg |
| tgacaaacaggtcatattttcaccctttgcgaaatttctgagcaccgctatacctattac |
| aggtttacaatcattgtgcgctgttttgttggacattattgtgccctgcgattgtctact |
| gaggaaggaagtgcctgagatgcttatgaagatcttaaaaacaatgtagtcacgcacctc |
| agagtgattcagggaatacaatattgcaagaggatataaggaattcagaagcttagaatg |
| t |
| >TCONS_00013580 gene=XLOC_008264 |
| ggaatactcctgagtcaaatttgtttcagattggttacagaaaaatgaaattcggctcca |
| aaatacaataatttacacctctggatacgtgcaacgattatttaccatgttgacttacca |
| tcgtctgttaaaaatttcacgaaaagatgctagttctggattatggttgggacttattgt |
| ccctgctttattacttaaaacagacaataaagcacttgcctacgaattttttctatctgt |
| aattactgtactcttcttaacaaagaataatcattttaaacatgctgtcattcctatatg |
| tatatggttaaa |
| >TCONS_00012347 gene=XLOC_007663 |
| ggcaaagttgacaaaatcggcttggaggtcccactctgtaattatgtagcaagcgataaa |
| atctggcgcgataactgcattaaggaggcgagcgcagctaaagcatggtccaagaattgg |
| agcttccttactttaacccctcgggagctcctaaaagatgaactccacgaactgattgat |
| ccaaacagaaaacctattgaaatcccacaatacttgaaggtggccgaagcagtacctatc |
| tcggcctatataaaggtggaaccttcgccgaagcccatcccgcaaacaacaagtagaatg |
| attggatggagatctggactgcctcagtacaaactagacaagtatgaagtagcaaaaaga |
| cctcaaggttcgcttcttaagaggtttaactggccaatagaagctttatactgatgttaa |
| atttgacttgggtagtggattggaagcgccagtctcataagtccctgagagagatatgtg |
| aaaaacccgtccttcgtacgtaagagtactatgcctccatttctcccatagttcaaaatt |
| gacattgaagttcaat |
| >TCONS_00012375 gene=XLOC_007679 |
| atgtgactgtttggaagtcatgaacgtaaaactaaaacttcaaaaattttggataactgc |
| acttaataaatacccacattttgtgtatattgtaccactagggtcagtatgtgtctatgt |
| ttcgattacaaagcatctccgtttgcgtgctttgggaccagaagaagcttataagtataa |
| aaaaatgtatgtagttaaacgagctgaggatgttgaactaactgaggacaatgcacacct |
| gtacaactgactagcgatcacacagtgtttcatgttcagtttagctttagtactagaatg |
| aaaggtttcgtggtgctaaactgtgtttttggctt |
| >TCONS_00012376 gene=XLOC_007679 |
| acgtaaaactaaaacgtgaggattaagaattgggtgacttatattgctattgtagttcaa |
| aaattttggataactgcacttaataaatacccacattttgtgtatattgtaccactaggg |
| tcagtatgtgtctatgtttcgattacaaagcatctccgtttgcgtgctttgggaccagaa |
| gaagcttataagtataaaaaaatgtatgtagttaaacgagctgaggatgttgaactaact |
| gaggacaatgcacacctgtacaactgactagcgatcacacagtgtttcatgttcagttta |
| gctttagtactagaatgaaaggtttcgtggtgctaaactgtgtttttggctt |
| >TCONS_00013638 gene=XLOC_008293 |
| gatcatatcaacgtttgtactgtgttcactgatcacttatttctccttatgctccaggaa |
| tatcttttcattgcatcctctgtttatgagttttggatttctgttctttatgtttaatgg |
| tatagatgtactaagtgaaaacattttcagtcccttgaaatccaggtctaaaaagataaa |
| agcccactggatactcgagtctctttcagtattatttattttcattggttttattggaat |
| ttacgccaacaaaatattaaacggaaaaccgcatttcgccacatggcacggacttatagg |
| cgcagtcgcagtattttactctgtcttccaactatttatgggtttagtgtttcactttac |
| ttataatctctggagaagcttattggcttacagtaatggaaggtttcttcatgctgcatc |
| tggtgtggtgttgtcttgtcttttatctctcactttcatacttggtctttatacaacttg |
| gtttaaaacatatgtcgttttatattttaattcatttagtcctttagtcttgtatttatg |
| tgtagttgtagttaccctgagcactttcaaagtttttctgcaggtagttacgaagttctg |
| caaatgtttcaaaataaaactacttaattctcagagagacgtacaaagataaaatttata |
| tggtattttaattgcgcctgagtccttatgtttatcgctattattgcttgtactaatttc |
| gtctatttgtcttaatattagtt |
| >TCONS_00012476 gene=XLOC_007735 |
| cggtgggcaacttggaggaggtctgacctttttacagtgccttgcatgatgaatgaatct |
| gtctgttctgtctttcgtgacaaactaaagcgtttacttcactggggtcccatctctacg |
| atctggattatattttttattacactcacgagtttatacagtaccttacatgttgcccct |
| ccctgttcatcacttcttggtctgtttctatctacgtgcatatttagttccttttatatg |
| attctgaagtcttacctttgtgctgtatttgttggcccaggatttgttcctctgggttgg |
| aggccgtgtgacccttcagctgaacagaagcttcagttctgtaatgtttgtagaggtttt |
| aagcccccacgagcccatcattgtcgagcttgcaaccggtgcataatgaaaatggaccat |
| cattgtccctggataaacacatgttgcggtcatcttaaccataaatattttttgatattt |
| ttgttgtttgctccatttggatgcatcacctcttgtatagcactattgctttctatatat |
| caaagtccagcaatcatgcggcttgtaattggttttcagtagcagaactaatatgtactg |
| tcttattccagctacatttttcactccataagtcatgtattagtctcttgagaggatata |
| gatatggcgtgaacgttttggtggtatcagtgtttctaacttcctttggcagattagtct |
| atcctgttacaagattttgtgacttttgtggtgacattgttttcttccaattacgaagtt |
| gtgttcatggtttttcaagatccgtaagtgattttcgtggaaaaagacccatgcccacgg |
| ttaattatagtttctttttacgtataatttggtttgcacgaaaatccatgagtactaaat |
| ttatgttaaaaatgctggtcttcataaaacgtaagcattttcgtttgatttcagtttcct |
| agtttacttttgcgaagatttcaatcaagcgttttgttcgtcatagctgatctaatgata |
| acattatttgctttgggtttagctgtgggtgttgcactgtctgttgggattttagccatt |
| ttccagttgaaagctgttgcaaggaatcaaacgggaatagaatcatggatcgtagcgaaa |
| gcaaacgtttggcgaaaagatgtaggggaaaaaaaaccatttcgctatccatatgacctt |
| ggaaagatcggaaattttcagcagatttttctgtggtctggtaaagtcttaggagatgga |
| tactactggcctgttgtgaaaggctgcactcagtatgacttgactttggaacagatctat |
| caaaaaaggttgaagcagaaaattcagcgcacattcaaaattacccgaaattatgatggg |
| agtcggtgtttgtgttttcgttatggatgtctaactgcgatacgttccccatgttttgaa |
| gaacccagaatacctgtacgtgtcggtgatgttcttatggtgacaagaggaacaaagtac |
| tggatttatggtcatttggtcccttctgagagttttggtgatttttcagattctgtcgaa |
| actcgtggatgggttcctcgtgtttgtgctttggaagtcggtttcaagcacaaaaacgac |
| aagttctctcttaaaaacgactaggcaatcacattccatcgttcagaggacgatccgttt |
| tctattttgtaaattactacagtgttcgtactaattatcgttactttactcacttttgta |
| atttcttttcttgttctgttctttcattttattttcctatttactttctcttatctaaaa |
| gattgagtgggatgaaattattctaataacacaaactgaacatctcagtctaagatttgt |
| ctagatgtattgtttaccatcattctaaccaccaatcttatgaagtgacccaaatgttag |
| aggagcgtactagtagttggtgtttgtttaacacaagcttttcatatag |
| >TCONS_00012477 gene=XLOC_007735 |
| cggtgggcaacttggaggaggtctgacctttttacagtgccttgcatgatgaatgaatct |
| gtctgttctgtctttcgtgacaaactaaagcgtttacttcactggggtcccatctctacg |
| atctggattatattttttattacactcacgagtttatacagtaccttacatgttgcccct |
| ccctgttcatcacttcttggtctgtttctatctacgtgcatatttagttccttttatatg |
| attctgaagtcttacctttgtgctgtatttgttggcccaggatttgttcctctgggttgg |
| aggccgtgtgacccttcagctgaacagaagcttcagttctgtaatgtttgtagaggtttt |
| aagcccccacgagcccatcattgtcgagcttgcaaccggtgcataatgaaaatggaccat |
| cattgtccctggataaacacatgttgcggtcatcttaaccataaatattttttgatattt |
| ttgttgtttgctccatttggatgcatcacctcttgtatagcactattgctttctatatat |
| caaagtccagcaatcctgtgggtgttgcactgtctgttgggattttagccattttccagt |
| tgaaagctgttgcaaggaatcaaacgggaatagaatcatggatcgtagcgaaagcaaacg |
| tttggcgaaaagatgtaggggaaaaaaaaccatttcgctatccatatgaccttggaaaga |
| tcggaaattttcagcagatttttctgtggtctggtaaagtcttaggagatggatactact |
| ggcctgttgtgaaaggctgcactcagtatgacttgactttggaacagatctatcaaaaaa |
| ggttgaagcagaaaattcagcgcacattcaaaattacccgaaattatgatgggagtcggt |
| gtttgtgttttcgttatggatgtctaactgcgatacgttccccatgttttgaagaaccca |
| gaatacctgtacgtgtcggtgatgttcttatggtgacaagaggaacaaagtactggattt |
| atggtcatttggtcccttctgagagttttggtgatttttcagattctgtcgaaactcgtg |
| gatgggttcctcgtgtttgtgctttggaagtcggtttcaagcacaaaaacgacaagttct |
| ctcttaaaaacgactaggcaatcacattccatcgttcagaggacgatccgttttctattt |
| tgtaaattactacagtgttcgtactaattatcgttactttactcacttttgtaatttctt |
| ttcttgttctgttctttcattttattttcctatttactttctcttatctaaaagattgag |
| tgggatgaaattattctaataacacaaactgaacatctcagtctaagatttgtctagatg |
| tattgtttaccatcattctaaccaccaatcttatgaagtgacccaaatgttagaggagcg |
| tactagtagttggtgtttgtttaacacaagcttttcatatag |
| >TCONS_00012478 gene=XLOC_007735 |
| cggtgggcaacttggaggaggtctgacctttttacagtgccttgcatgatgaatgaatct |
| gtctgttctgtctttcgtgacaaactaaagcgtttacttcactggggtcccatctctacg |
| atctggattatattttttattacactcacgagtttatacagtaccttacatgttgcccct |
| ccctgttcatcacttcttggtctgtttctatctacgtgcatatttagttccttttatatg |
| attctgaagtcttacctttgtgctgtatttgttggcccaggatttgttcctctgggttgg |
| aggccgtgtgacccttcagctgaacagaagcttcagttctgtaatgtttgtagaggtttt |
| aagcccccacgagcccatcattgtcgagcttgcaaccggtgcataatgaaaatggaccat |
| cattgtccctggataaacacatgttgcggtcatcttaaccataaatattttttgatattt |
| ttgttgtttgctccatttggatgcatcacctcttgtatagcactattgctttctatatat |
| caaagtccagcaatcctgtgggtgttgcactgtctgttgggattttagccattttccagt |
| tgaaagctgttgcaaggaatcaaacgggaatagaatcatggatcgtagcgaaaattgatt |
| gaatcagtggcttgtctatatgtgaagtttcatcactgtgactattttaatctatttagt |
| ctcattatttttgaaggcaaacgtttggcgaaaagatgtaggggaaaaaaaaccatttcg |
| ctatccatatgaccttggaaagatcggaaattttcagcagatttttctgtggtctggtaa |
| agtcttaggagatggatactactggcctgttgtgaaaggctgcactcagtatgacttgac |
| tttggaacagatctatcaaaaaaggttgaagcagaaaattcagcgcacattcaaaattac |
| ccgaaattatgatgggagtcggtgtttgtgttttcgttatggatgtctaactgcgatacg |
| ttccccatgttttgaagaacccagaatacctgtacgtgtcggtgatgttcttatggtgac |
| aagaggaacaaagtactggatttatggtcatttggtcccttctgagagttttggtgattt |
| ttcagattctgtcgaaactcgtggatgggttcctcgtgtttgtgctttggaagtcggttt |
| caagcacaaaaacgacaagttctctcttaaaaacgactaggcaatcacattccatcgttc |
| agaggacgatccgttttctattttgtaaattactacagtgttcgtactaattatcgttac |
| tttactcacttttgtaatttcttttcttgttctgttctttcattttattttcctatttac |
| tttctcttatctaaaagattgagtgggatgaaattattctaataacacaaactgaacatc |
| tcagtctaagatttgtctagatgtattgtttaccatcattctaaccaccaatcttatgaa |
| gtgacccaaatgttagaggagcgtactagtagttggtgtttgtttaacacaagcttttca |
| tatag |
| >TCONS_00012506 gene=XLOC_007748 |
| ggatacaaagggacttaggaagttgattgaggataaggcaaacgatgcaatagaaggaat |
| acaaagatgtcgcgatacatatcgaagagagatggctgagtgtaagcactttgtgatcga |
| taaagtatctactttttttgctgatttagaaatgcgaagtcaaaaacaagaaaacgtcga |
| tgacg |
| >TCONS_00012515 gene=XLOC_007754 |
| gaccttgcgcgcaaatcaatagtcgtaatcttaggacctaatgacgaaaataattatttt |
| gtttgtcagtgcatacttttaactaatcagaagacagactaccttttgggactgaagtat |
| cgctgatgtttatcattatcattaaagatgggtgggcatgttagtcgtggtcgagataat |
| cagtcgctcattgacgaactggtgcgaaacggtctcacattaaacccagagactgagcgt |
| gctctacgtctagtcgacagagggggttacttcagcgagaaaagcccgcgagcatacatg |
| gatatggcatggaggtctggttctctccatctttcagcacccagcatctacattgtagct |
| ctcaaaaacctcgatattcaaccaggaaattgctttttaaatgttggtagtggaacagga |
| taccttagtacagtcatt |
| >TCONS_00013779 gene=XLOC_008361 |
| attcactaatcggttattcaagcgaaaagttgattttgttttaaaagctatccactctat |
| cccccatacatccgcacataaataatatatacattcatatatgtaggatacttggataca |
| cacatgatattttatacaacttcaatcattttcattcaattatcttgtatttattcacaa |
| acttatgaatctactcagctgatcatagataggtgctttgattaagaaagatgatgtagc |
| tcaagaaatatgtttcaagaaaagtatacaagcagcaaacaaactattacagcaacacga |
| ttcaacgaatcaccggtttcaattaataccaataatagaaacaattgatggagatgacag |
| tttcgaagcaacgcgaaaagcctgtcagttaattgaacgtcaagttatcgccatttatgg |
| tccatctacaccgtacgcatcgtcggctgtacaagctttatgcaatcagttcggtatacc |
| acacttacaaattgattggggttaccatcaaacgtctcgcggttatgcattaaatgtaca |
| tccacattatttagcatttgggcaagctttatatgattatgtacagaaggcggaatattg |
| ggatacagtagctgtgatttattctagagaagaaagt |
| >TCONS_00012537 gene=XLOC_007769 |
| tttgttgcatcttaataaccatgtcacttacacaaacatatcagtctggtcaagtcactt |
| ctctggccgtatacaatctacccagttcggcagacgaaacagaagttcagtcggtattcc |
| cttcggccaggtcagtcaacttcgtacgaagtcattctgtgtcttcgcaatctagaggca |
| tgtgcatcttgcaattcaacaatgctcgtgactgtcaacaagcctacgatgaatgtcttc |
| agggaaaagaaattggtggacagctggtccatgcagaactgaacggagattatcgttcca |
| gttcgtcagaaaatttaaatgtcagccagcaccatggagtcaattccagggatttcagac |
| gttcacatgccgataatgagtacagccagcccacatatgatccgcatggtagaactgaaa |
| gtgaaggttctgccaacgcctgtacacttactgtgtcaaatcttccctatacagcatctg |
| aaagagatattatgcgcgagttcccggaagccctccgagtagcactttcgttagacgagc |
| agggtcgttctagaggcgttgctcacgtgactttctcaaactccgaccaatgcagcgccg |
| ctcttacttcctgtggcaataagatgatgggcggtagacctgtgcgtggacgaatccaga |
| gagaccaggagcacagccaaccgagttataataatcaacgctcttataaccgtgaccaaa |
| gagaatacggtcaacgtaatcaacgtgaatacaatcaacgtaatttcaataatcgggacc |
| acaccaaccagcgtgaaaacggtaaccaaagagatggcaatcaacgtgagtacaatcagc |
| gtgattatagtggtcaacgtgactataacagacaaggtaggcagttcgactacaattcag |
| gcagaggcgaaagagacgtaaaccgcttccaaggtgcaccagctaatcgaggcgggtcac |
| gcgagtttggtagaaacgatcgcttcgaacgaacagatcaaagacgagatcgcagcccag |
| ccagaggagaaccagttcgcggatacggtagttcaaaggggccccgaataacatcagctg |
| taattcaccgtccaggaaatgccagcccttcggagtcctcctcttcagatgaggactaac |
| catcctggtattttttcagggatttccttactggtgtggcgtgcaaatacttttattaac |
| gtacatatgactttaacattaaaacactaaaatatttatgtt |
| >TCONS_00013812 gene=XLOC_008377 |
| tcagattttaaaacataagcactcttcattacactttactctagcgagattgttctactt |
| atatttaaaatccgattggaccgatcaaaagttgttaaagtagtaattcaaataatgaat |
| ccctggaaaatatatattccatttataaagcctctatacttaagtgattgcggttgcata |
| atgccttgtattcagccgacatcttacgtagttacattgaattcggtttgtcagtggtaa |
| aaatttctcatttttgtaccatactggacagtaccctcagaatgatctactatcagctta |
| gtgaatagtgttcaccttagttgaaagttattaaaattctttctacacatttcaaactat |
| ttcgtatatcgatgttagcgcttactctcgttacattttttgttataatcagtgattata |
| ctgaccatatgagaagcaaaatttactgataacgacttctccagaccataaaagcatgag |
| ttatttagctgcatagttacaattatcgttagaacgaattgttccaataataaacaacgt |
| gaagcctggcacaattttccgtcggatccatttggcacacataacatctgaacagtaaaa |
| gagagaaggctaacaagttaagctcggaagaatcgtgttgataatagcaatagcaggaac |
| acagaattgagtttctaagaggacaaatgacaatgtggatagaaaagctaaagaatgtat |
| acatctacactaactcaatcgatttcgagccacgatttaatttcagttttttgtttcctg |
| agcaatttcagatatgtaattcacttgttttggcaaagaaaaatactaaatctgttgggt |
| ttgatgtaaaaaat |
| >TCONS_00013835 gene=XLOC_008390 |
| aggcaaagactattgggttcgagtacttgacagtatttcacactcagacatttcgccacc |
| tgctttggtttaaacagctacatagtaccgctggtccccatgcaagtgtgatccgaagcc |
| gaatacataaacctgtacatggtaaatgagtgtaataacttgattgtgttttgtaatgga |
| tacatcataaaggcattctgtttttgttttgcagatgttttgcctgcagctgataatccc |
| actgctttttttaccgaaaccatctaacccggccgcagatatctatcatgcaatatatat |
| acttttatttcttgtttgtttcttcttagagcgtaaaccatgtggaatatgtgctattat |
| tttgtttattttcattattcttccatgttatagttccttagataatttatgtattttcac |
| cacatgcagtaaaggccgaactttgacgtagtgtctgacgtatatatatattctgacttg |
| accattgttatggtgtaatgtaaatattgttccccgagtagagaacccttctcttgcttt |
| catttgaaatttccgagatttcatgatggattagccgatttttacgagttggtatggata |
| tttacaccaacctttgtgcgcatagtttttcaatttacaatagatttttggtgactatat |
| acct |
| >TCONS_00013851 gene=XLOC_008398 |
| ctcaacgtaacggtttatcctaggctattgtgtcatatactacagagtcatttgatgagt |
| atttgtacgacagttttttccatctttctgcgtatttaatccctcacaacgaatccctgt |
| acctgtcttatcatcagcctatgacttcacatcaccgctagcacagaacataatacattg |
| tctctaaaagacgtcatgagttgcgctgttatgtgcttttgaattctttcaacaattaga |
| attcacactttttaattgcaaggattccaatcattcatcactgtgtttatgagatttatt |
| attattttatcgtagtatcatttttgtcacaaaccttacaattggatcctaatgttcatc |
| tctcacagcatatttgtttgtt |
| >TCONS_00012611 gene=XLOC_007810 |
| gaagcttgtgaggtttcttatgaaattgtcccatgagacggtaaccattgaactaaaaaa |
| tggtacacaggttcacggaagcattgctggtgttgatgtgtcaatgaatacacacatgag |
| gtccgttactttgacacttaaaaatcgggatgcgattaacttggacacattaactgttcg |
| cggaaacaatatccgatatttcattctcccggaaagtcttccacttgatacacttttaat |
| tgatgaaggccctccaaaaagaaaacctggacgggaagaacgtcctactattcgtggacg |
| gggtcgcggtcttgttcgtggtcgtggacgcggtggacctcgaggacgtggtcgtggtcc |
| tccgatgcgttactaaaatctttttacattatgtatatgtctaatcactgagcact |
| >TCONS_00012629 gene=XLOC_007819 |
| gtcatgattacgctcccgcatgacagtaataatctccgacgttatagcaagaaggtccac |
| aaaccgcgatctaaaaaactaaaggtggggtcccgtctttctcatcgagtggtcaatgaa |
| tggaacgcctacccgaaaaaatggtatcagcctcatcagcgaacattttcaaggaggagc |
| tggatcatcactggaaggcgaactgtcaggattaacacatgttcaccaaactaatatcct |
| tatcgctgaaaactaaggacatactttatctagatttatatctcggactatggtaataat |
| agttgaaattactaattgtggataacgggacccaaagaaagtcgcacacgcttattcctg |
| acaatttaagtaacgttctgtatatttttcttgtgtgtg |
| >TCONS_00013977 gene=XLOC_008463 |
| ggaaccaaaaactgaaaaacgattccatgttgaattacactttagtcctggtgcttttgc |
| attatgtcacgatcttcctgaagggagtggttttcgatctggaaaattggtgcgtctgaa |
| ttctcaaatgagcgtaatgagtagcggtacatctgtggataaaggaagttctgactcagt |
| gccacaatcagcaatcccttctgttacacctattgttcctgctagtgaacaagacaagaa |
| aggtctgtttgatagatgtagtagtagacaaacagaacaaagtagtgaatatcaccagaa |
| tcttgggtcatttgactcaagtgaatgttcattctcggaatcttccgctaacgataaagc |
| ttcagatttgtttcgttggagttttgatagtgataggaatacgtatgaagtatttaagaa |
| tcaagcagagacttttaatgattcctctcttatcgaggaatgtccagttcgcaggtcaca |
| tagtgttcgttgtaacctcgcttcaaataattctcgagaaaacattgtaaatcatgaaga |
| aactttgattgtcaatcacagatcacaatctcaaggtcgtccttctgttttgccattatc |
| acctgtaccatcaacctctacgctagaattaactggaactcctacagcgtcaaatacgga |
| agttggtattggacgattcactgtcacgcggttcactgataataaaagtggttcgggtcc |
| aacttttccaagtgaatcaaaaactgataaattattatcgacatcaccttatacgaataa |
| actgaaaacgtaccaccttagacatttttcacgttcaaatttctgccaacattctgcccg |
| taataggtctaattcatgtcatcctgatgtttacagaaggacaaatttaactacaaccct |
| attatcaaaacctagatattcatgtatttcaaaactacttcaaacgacaggtaagcttaa |
| tgattttggcaagtttaagaaatatgattataatgaacaactgtcttttgaatgttcctg |
| ttcaaatactaattcgtcacatttcgctcctgtaattcaatggctttcaaagcagtcaga |
| acacttagtgaaatcagatgaaaaagatgacaatgaggatcacaaatctaatccaatcaa |
| catagaagacaagaaagttcgggaaatgcctaaatggctaacctcggggactcgtaaaat |
| gtacgattcagctggttcaaataattctactagtttgaatcaacataatagcacatgttc |
| tgtccattcatcaaaaaatcaagatttaacttccacatctactcgagttcgtttaactcc |
| actttcttctgatgaaccggtgtctttcaatacaaatgaatcgactcaatttacaatgga |
| tttaccactcaatcaaattaattctcaaccagtcaacaattttttgtcatctggggctga |
| cagtggtgtagatgatgatcctgttatcaatcaaactttgaatgctatcacttatcctag |
| tcgtcaaagcccatatcatttagatattgggcgtcttgtaagagctgttgcctcgggagt |
| gccgtttgcaccgccaatatgtcgaagtcttataagtacagcggttatacgtggttcacg |
| tgatgataaccaagcatctagtagtgttcctgatttcaagagattaacagaggatataag |
| cgtggcaccacgaacattaacacctggaagctctcaagaatttttggttcccagtgctcc |
| atgtgtcccagaagtttatcccctggaaacactacataacgctttgactttaagtcaatt |
| ggaagcttttcttgttcgtttaactactcataagtttccgactccattcacatcaccgaa |
| acacccatctactcctgtaaattattatcaccatcaccatcaacctcagttaagtcaaac |
| tacccttgagtccttctctcagtatcatgacttatgtactaatctatcttcctcacttcc |
| aaattctactggagatgatactgcaacacaggattgtcccagtgttgctgatgaagcctc |
| tgctcaactacagcaacctgtcttatcaaatgaaacaccaaacgttatgtcaataatccc |
| atccgcctcactgtttcatgaaattacttccagaaatacagatgaccaacagtaaatcaa |
| aaaattccgacagctttttcttccttaattaacaaaagtcgatttcttcatttatggata |
| gttgaagaatatcacaagttcacttttgcctctgcttctgtatactgtgtaataaacagt |
| tttctcctaaacttatatgatccctttcatctagatattgatctctaattaagcgaatat |
| ttattagttgttttactttgttgtcaatattttcttagaattttttcagtttgtctgtat |
| ttgtttactcattagttctttgtttgtatataatggttttctaatat |
| >TCONS_00013978 gene=XLOC_008463 |
| ggaaccaaaaactgaaaaacgattccatgttgaattacactttagtcctggtgcttttgc |
| attatgtcacgatcttcctgaagggagtggttttcgatctggaaaattggtgcgtctgaa |
| ttctcaaatgagcgtaatgagtagcggtacatctgtggataaaggaagttctgactcagt |
| gccacaatcagcaatcccttctgttacacctattgttcctgctagtgaacaagacaagaa |
| aggtctgtttgatagatgtagtagtagacaaacagaacaaagtagtgaatatcaccagaa |
| tcttgggtcatttgactcaagtgaatgttcattctcggaatcttccgctaacgataaagc |
| ttcagatttgtttcgttggagttttgatagtgataggaatacgtatgaagtatttaagaa |
| tcaagcagagacttttaatgattcctctcttatcgaggaatgtccagttcgcaggtcaca |
| tagtgttcgttgtaacctcgcttcaaataattctcgagaaaacattgtaaatcatgaaga |
| aactttgattgtcaatcacagatcacaatctcaaggtcgtccttctgttttgccattatc |
| acctgtaccatcaacctctacgctagaattaactggaactcctacagcgtcaaatacgga |
| agttggtattggacgattcactgtcacgcggttcactgataataaaagtggttcgggtcc |
| aacttttccaagtgaatcaaaaactgataaattattatcgacatcaccttatacgaataa |
| actgaaaacgtaccaccttagacatttttcacgttcaaatttctgccaacattctgcccg |
| taataggtctaattcatgtcatcctgatgtttacagaaggacaaatttaactacaaccct |
| attatcaaaacctagatattcatgtatttcaaaactacttcaaacgacaggtaagcttaa |
| tgattttggcaagtttaagaaatatgattataatgaacaactgtcttttgaatgttcctg |
| ttcaaatactaattcgtcacatttcgctcctgtaattcaatggctttcaaagcagtcaga |
| acacttagtgaaatcagatgaaaaagatgacaatgaggatcacaaatctaatccaatcaa |
| catagaagacaagaaagttcgggaaatgcctaaatggctaacctcggggactcgtaaaat |
| gtacgattcagctggttcaaataattctactagtttgaatcaacataatagcacatgttc |
| tgtccattcatcaaaaaatcaagatttaacttccacatctactcgagttcgtttaactcc |
| actttcttctgatgaaccggtgtctttcaatacaaatgaatcgactcaatttacaatgga |
| tttaccactcaatcaaattaattctcaaccagtcaacaattttttgtcatctggggctga |
| cagtggtgtagatgatgatcctgttatcaatcaaactttgaatgctatcacttatcctag |
| tcgtcaaagcccatatcatttagatattgggcgtcttgtaagagaaactgaacagatcta |
| cagacgtttcagttctgcagcccctgttgcctcgggagtgccgtttgcaccgccaatatg |
| tcgaagtcttataagtacagcggttatacgtggttcacgtgatgataaccaagcatctag |
| tagtgttcctgatttcaagagattaacagaggatataagcgtggcaccacgaacattaac |
| acctggaagctctcaagaatttttggttcccagtgctccatgtgtcccagaagtttatcc |
| cctggaaacactacataacgctttgactttaagtcaattggaagcttttcttgttcgttt |
| aactactcataagtttccgactccattcacatcaccgaaacacccatctactcctgtaaa |
| ttattatcaccatcaccatcaacctcagttaagtcaaactacccttgagtccttctctca |
| gtatcatgacttatgtactaatctatcttcctcacttccaaattctactggagatgatac |
| tgcaacacaggattgtcccagtgttgctgatgaagcctctgctcaactacagcaacctgt |
| cttatcaaatgaaacaccaaacgttatgtcaataatcccatccgcctcactgtttcatga |
| aattacttccagaaatacagatgaccaacagtaaatcaaaaaattccgacagctttttct |
| tccttaattaacaaaagtcgatttcttcatttatggatagttgaagaatatcacaagttc |
| acttttgcctctgcttctgtatactgtgtaataaacagttttctcctaaacttatatgat |
| ccctttcatctagatattgatctctaattaagcgaatatttattagttgttttactttgt |
| tgtcaatattttcttagaattttttcagtttgtctgtatttgtttactcattagttcttt |
| gtttgtatataatggttttctaatat |
| >TCONS_00014089 gene=XLOC_008519 |
| agcgggaaatgttatgtggtcgatcatcggacgtttctgaaacaaaaggaagtgtcttgg |
| acaaaaacgatgttgctaaagacagtgcatcaaatgagactggacagcgcgatttggacg |
| tgaaatccaggactcaggatgaggacgaactgttaggtagatcagatgaaaatcacgact |
| tgtcatatgaaacccattcgaacgagaccggcagttttagcgagttccacactgcagttg |
| agtgccttaacgattcggaagaagggagtggtgacttttccatcaacacgaaaaaaaaca |
| acttcgtcaaaatctctaccgttacatcagaggatgtcgatccttttgaggatgtcactg |
| ctgccaccaatgaaataaccgacaaaaaagcaatttcaccgattgaatgggatagagttt |
| ctgatgaagaaagcgagaacagagacgaacttcatgtcgatgccccaggtgatgagtttt |
| ctaaagaggaagaagcccaaaactcagttcggccctctagtgtcagagggcatacctgca |
| caagtgagagagaaaattacattgattcgcgcctttttcgttctacaaagtatttcctca |
| tcaagagtaataattttgaaaatatcgaaatagcgaagtctcgcaatgtatgggcgacaa |
| caaagggcaatgagacaagactgaataaagcattttttgactacaacaatgttttgctca |
| tattttctgtacgtgagagtggtagattccaaggattcgctcgcattatcgcctcctcag |
| atcctcgaatcaaggttgactgggtgttttcttcacgcatgaatactggtcttctgagta |
| atccttttcgaattaagtggatatccaagtctgatctccctttcacaaagaccggacatc |
| tcttgaatgcatggaatgaagacaaacctgtgaaaatcgggagagatggtcaggaaattg |
| aacctacatgtggtgaggcactatgtcggttgttcaataaggacgaacttggggaagaac |
| aaaaacaactaaacatagttcaagctattggtgaaagaatcagtcgtagaaatgcatttg |
| agagacttggaaaaattgttggtcgccatggtcaaacgagtcataatggttctcgtgttg |
| ttgggttaaacaatcaagacaacactggactgttgggggatgctactggaacaaaccaac |
| gatatatggcatctactgaacgaaggagcttggtgaattaccctcctataacacctttaa |
| tcaacacagatggttcgcggctaacctcgacgggaactacaccgctattgcaagtaaacg |
| cagctgccgctgcaatggcagcagctgcagctatggctgcaatcaaatcatccggaccac |
| agtcaataggtgcttttgcccgaagtggagcaagcactctcaatcccacaattaattcca |
| gactgcctatacaaccacctactttccctcacatattttcaaccttcccgaattcaggtg |
| gttctttctcacgtttgaatgtatctagttcctctaacgcaactctgcttccgttgttgc |
| aacaacagcaacttcgtatatctggatcagcagcaaatgccgccgcaggtgctgttcttt |
| cacaagctatacaacaagctcgtagtctatccaatcctaatatgataagtcgatcaggaa |
| tgcctgtcaatcctgcgtcaaacagtgtgcctgtaaaatctcataccgaaagaagtcgtt |
| cgcgctctgctagcgacagcacagcttacagttatgctcgggaacgtaatccaaggcatc |
| ggagaactgacggttatgtaaaagaagagtctccacgatttgaaacaattcgacggaagt |
| cacattccccgaggttaggtgttattgaacagtggacacaagaatcaaaaatttgtgcta |
| atttatttattatttatatatggactgtttcacatctagttaaccacattgttttattgt |
| gcttcacttgaactgtcgaatttttttcatttttgatgctagcaacttgcttgtaccatg |
| gccacatgtggcttccatgaacaattaacattagatttcatatttttggatatccggtta |
| ctaacatagttgtaatagtaggcacgtgaagaactaaaaaatcttcagaggttcttgtat |
| acatcgaacatgcttattttgtaagtgaaatttggcttaagtttgcttgtgttgaggttt |
| tattagtcttcatttcctgaagtgaagttaatgtttggtggcatatgatttaacccattt |
| ctttttaggaataatacaggtaaaggatgtttgtgggtatttaattccctgttcacatgt |
| atttagcattaacatttgttttcattttcaataatgtgtcttcgttcacatgggaactct |
| tggtttggtttctgtcgtcaaggaggcgtgaaataacatcaaggcatggaaattatgaaa |
| gacctcgtggacatagatctcgtagtcggtccagttctcttcctattgaccgtaatttat |
| tgatgacttgggactatgacgactatgttcaacaagttagccgtggtatgcgaccaaaca |
| tgtatgatgaattgggaagtcgagttgccggttcaagtgcttattcacacatttcccaaa |
| gtcatgaaaatgatctgaaatatgaagaaaaagtagatgcgtttttacgtggtattggat |
| cacgcccaaaagttgcaggacatgatcatcactactcacgcagtcctagccgtagctgtt |
| catatagtcctcctccacctcatccccacaaccgtcgtcgtgaatattgcaggggttcat |
| catcctactccagatccaggtctccttccggaccaggcatacttttgtctaaggttcgac |
| gtggagagatagatcctgatgcttatcgtcaccgcccaccatctcaatatttatcccctc |
| gtcggagagaccatacttgttcccacagccgctctcctgcaagcttcggtcgtgagcttc |
| cagcacagtggagaaagccttcacaggcttccgtagctcattcaactggaagtagacgac |
| accagcaaatagaggggcgagttccttcacaccaagcacgatcccctcgtgttctctcga |
| gtggagggtcacgaacgtacactgcgagcagtcgtcatcacaaatagctggtttatgttt |
| attcagatgatcgtcgctctctgatacttctcgta |
| >TCONS_00014090 gene=XLOC_008519 |
| agcgggaaatgttatgtggtcgatcatcggacgtttctgaaacaaaaggaagtgtcttgg |
| acaaaaacgatgttgctaaagacagtgcatcaaatgagactggacagcgcgatttggacg |
| tgaaatccaggactcaggatgaggacgaactgttaggtagatcagatgaaaatcacgact |
| tgtcatatgaaacccattcgaacgagaccggcagttttagcgagttccacactgcagttg |
| agtgccttaacgattcggaagaagggagtggtgacttttccatcaacacgaaaaaaaaca |
| acttcgtcaaaatctctaccgttacatcagaggatgtcgatccttttgaggatgtcactg |
| ctgccaccaatgaaataaccgacaaaaaagcaatttcaccgattgaatgggatagagttt |
| ctgatgaagaaagcgagaacagagacgaacttcatgtcgatgccccaggtgatgagtttt |
| ctaaagaggaagaagcccaaaactcagttcggccctctagtgtcagagggcatacctgca |
| caagtgagagagaaaattacattgattcgcgcctttttcgttctacaaagtatttcctca |
| tcaagagtaataattttgaaaatatcgaaatagcgaagtctcgcaatgtatgggcgacaa |
| caaagggcaatgagacaagactgaataaagcattttttgactacaacaatgttttgctca |
| tattttctgtacgtgagagtggtagattccaaggattcgctcgcattatcgcctcctcag |
| atcctcgaatcaaggttgactgggtgttttcttcacgcatgaatactggtcttctgagta |
| atccttttcgaattaagtggatatccaagtctgatctccctttcacaaagaccggacatc |
| tcttgaatgcatggaatgaagacaaacctgtgaaaatcgggagagatggtcaggaaattg |
| aacctacatgtggtgaggcactatgtcggttgttcaataaggacgaacttggggaagaac |
| aaaaacaactaaacatagttcaagctattggtgaaagaatcagtcgtagaaatgcatttg |
| agagacttggaaaaattgttggtcgccatggtcaaacgagtcataatggttctcgtgttg |
| ttgggttaaacaatcaagacaacactggactgttgggggatgctactggaacaaaccaac |
| gatatatggcatctactgaacgaaggagcttggtgaattaccctcctataacacctttaa |
| tcaacacagatggttcgcggctaacctcgacgggaactacaccgctattgcaagtaaacg |
| cagctgccgctgcaatggcagcagctgcagctatggctgcaatcaaatcatccggaccac |
| agtcaataggtgcttttgcccgaagtggagcaagcactctcaatcccacaattaattcca |
| gactgcctatacaaccacctactttccctcacatattttcaaccttcccgaattcaggtg |
| gttctttctcacgtttgaatgtatctagttcctctaacgcaactctgcttccgttgttgc |
| aacaacagcaacttcgtatatctggatcagcagcaaatgccgccgcaggtgctgttcttt |
| cacaagctatacaacaagctcgtagtctatccaatcctaatatgataagtcgatcaggaa |
| tgcctgtcaatcctgcgtcaaacagtgtgcctgtaaaatctcataccgaaagaagtcgtt |
| cgcgctctgctagcgacagcacagcttacagttatgctcgggaacgtaatccaaggcatc |
| ggagaactgacggttatgtaaaagaagagtctccacgatttgaaacaattcgacggaagt |
| cacattccccgaggaggcgtgaaataacatcaaggcatggaaattatgaaagacctcgtg |
| gacatagatctcgtagtcggtccagttctcttcctattgaccgtaatttattgatgactt |
| gggactatgacgactatgttcaacaagttagccgtggtatgcgaccaaacatgtatgatg |
| aattgggaagtcgagttgccggttcaagtgcttattcacacatttcccaaagtcatgaaa |
| atgatctgaaatatgaagaaaaagtagatgcgtttttacgtggtattggatcacgcccaa |
| aagttgcaggacatgatcatcactactcacgcagtcctagccgtagctgttcatatagtc |
| ctcctccacctcatccccacaaccgtcgtcgtgaatattgcaggggttcatcatcctact |
| ccagatccaggtctccttccggaccaggcatacttttgtctaaggttcgacgtggagaga |
| tagatcctgatgcttatcgtcaccgcccaccatctcaatatttatcccctcgtcggagag |
| accatacttgttcccacagccgctctcctgcaagcttcggtcgtgagcttccagcacagt |
| ggagaaagccttcacaggcttccgtagctcattcaactggaagtagacgacaccagcaaa |
| tagaggggcgagttccttcacaccaagcacgatcccctcgtgttctctcgagtggagggt |
| cacgaacgtacactgcgagcagtcgtcatcacaaatagctggtttatgtttattcagatg |
| atcgtcgctctctgatacttctcgta |
| >TCONS_00012887 gene=XLOC_007945 |
| agtcgaaatatcatgttgagttattgaaatgcagttcggacttgttgggactccaggtta |
| tacactattattctaatgctttcgtataactagaaaaacttaccaggttcagaaaatgct |
| tccgaatgaattcctagttcggctatatatattctgtacagcttcattcatcagttttta |
| cgttggagatcaattcttgattttattgtgctttcactattctttgtgcaaagtattctc |
| tcgacttagcagcaacagtttgtaagcatttttaaaatgtgaattgtgtaggttgcttgt |
| gatcacattaggcactatacttatggctgaaataatggctttgagaactttgttgccgtt |
| tctatggagtgctccagtcaatttattgtacatactactcattaattgtttcctctgcca |
| ttatttagtgtggtctgtgttagagttttcccttgctggtattgaacatgaggttagtct |
| ttattatttactatcagttagtaataggttcttgagatcccgactttcagtagcttgcca |
| acaatctgctgtctactgatcacgatgttgggctttggtcactggaagaaacatttgact |
| tcactaacatgctttcttgggtaacacttagtgtaattcctagtttttcagtgtgcctac |
| tttgtggctgtgctccagatataaaccgaaaaaacatcttgaagtcctttggaattcatt |
| catggaggcatgcatcgctgttttgctaaacctattccctgtattaaattatgccatatg |
| ttatttctttgatgaaagtggcgggtcactttttaaattcatggtcgagattctgttttt |
| agtaacgacaacaaacctgatatcatcacatcttcgtgttcaaagt |
| >TCONS_00012888 gene=XLOC_007945 |
| attcatcagtttttacgttggagatcaattcttgattttattgtgctttcactattcttt |
| gtgcaaagtattctctcgacttagcagcaacagtttgtaagcatttttaaaatgtgaatt |
| gtgtaggttgcttgtgatcacattaggcactatacttatggctgaaataatggctttgag |
| aactttgttgccgtttctatggagtgctccagtcaatttattgtacatactactcattaa |
| ttgtttcctctgccattatttagtgtggtctgtgttagagttttcccttgctggtattga |
| acatgaggttcttgagatcccgactttcagtagcttgccaacaatctgctgtctactgat |
| cacgatgttgggctttggtcactggaagaaacatttgacttcactaacatgctttcttgg |
| tgtgcctactttgtggctgtgctccagatataaaccgaaaaaacatcttgaagtcctttg |
| gaattcattcatggaggcatgcatcgctgttttgctaaacctattccctgtattaaatta |
| tgccatatgttatttctttgatgaaagtggcgggtcactttttaaattcatggtcgagat |
| tctgtttttagtaacgacaacaaacctgatatcatcacatcttcgtgttcaaagt |
| >TCONS_00012889 gene=XLOC_007945 |
| tttgtgcaaagtattctctcgacttagcagcaacagtttgttgcttgtgatcacattagg |
| cactatacttatggctgaaataatggctttgagaactttgttgccgtttctatggagtgc |
| tccagtcaatttattgtacatactactcattaattgtttcctctgccattatttagtgtg |
| gtctgtgttagagttttcccttgctggtattgaacatgaggttcttgagatcccgacttt |
| cagtagcttgccaacaatctgctgtctactgatcacgatgttgggctttggtcactggaa |
| gaaacatttgacttcactaacatgctttcttggtgtgcctactttgtggctgtgctccag |
| atataaaccgaaaaaacatcttgaagtcctttggaattcattcatggaggcatgcatcgc |
| tgttttgctaaacctattccctgtattaaattatgccatatgttatttctttgatgaaag |
| tggcgggtcactttttaaattcatggtcgagattctgtttttagtaacgacaacaaacct |
| gatatcatcacatcttcgtgttcaaagt |
| >TCONS_00014201 gene=XLOC_008577 |
| ggtaggtcctgggttggaatctcgagagtgtgggatagaggatacgcactgctgaggatt |
| cccacaataggacgaaacgaccgtccagtgcttccaggttttccatagtggtctagcttc |
| aattgacccatgatttcaattatttattaaagacacgatttatgatgatcgtgtctctcc |
| cattgttcaatattgcggagcttataagtttagagcttccaagaaagctgaagagggatt |
| gacagtattcttatcaacattctgggagagatcttcaagagctaaatgtaagggcccatg |
| gtgcaagagttgtttccaggtgatgactataataacctctccatcgcagcacttacgtgt |
| ctcattctggttagacaaactatactaaccatcctgttattttgtacatttacatttgac |
| ccagccaagcattcagatgtctttttatatataagacgagtcgccctatttaggatgaat |
| tggctagtgtgcttagtttatactgtcttcccatgtttgtcatttgcatgagtatttact |
| aacagctgagtgcataaaaaccggcacaccttcatcacatttcgtatttcttcattttgg |
| ctcaatattttaacagggtgacttatcgacgattaactgaaaaatgtaatatgagacaca |
| tgaatgagagatataggctccgaatgtcttaattataaactaaaggagttttaaatagtt |
| tctgaatcagacaacgttatacccaggaataaaacatcaagaaatgtgctttgctgactg |
| cagaatgccactgaaaaatgcatttggagtattccgcatactggtgatcaggccctctct |
| aaactccaagatacatggcccaacatacaaaaaattgccgtaagagacttcaataaaaaa |
| tgcggtccttgctt |
| >TCONS_00014317 gene=XLOC_008638 |
| gtagtgataaattacgaaaggaaaaattgcaaacaatgaaggaaaccggatgctatgttc |
| ctgaagcatttacagaaggaatgacgaaagatttagagaaagaacgtcgaaggcttcaac |
| ttgaaacttgtgaatatctgatcaaggtgaatgaattaaaagcaaagcgtggtgctgatc |
| tcctgcagcatttaattgatttcttctat |
| >TCONS_00015354 gene=XLOC_009592 |
| gatcttggaaattctgattctttgtacataatgtttcgtcaaccagccagttacatggaa |
| ctaattttctagaaaaatatttgcgatatctttttgcagttgtgttggtcgaaaatatct |
| agtcatgagtaattcagattttggtatcattgctttggagatctattttccaaaatttta |
| tgtctcgcaacatgatcttgaaatagcagatgaatgtgttggaaagtatactcaaggatt |
| aggacaaaaatctcttggattttgttcaatacaggaagatataaactcaatatgtctaac |
| tgttgttagcaaccttatcaggcgtataaatcttgacctgaaaacaattggtttccttga |
| ggttgggacagaaacgattatagataaatcaaagtctacaaagaccgtactaatgcagtt |
| attcgaaactgctggtaattttgacgtcgaagggacggatacaaaaaatgcgtgtttcgg |
| tggtacatctgctttgttcggtgcgcttaactggcttgaatctagttactgcaatgg |
| >TCONS_00015361 gene=XLOC_009597 |
| tctcttttcaagatagttgtatgtagtttacatgagggagttaaataccaagttgtcata |
| cgattctcatttgatcaacaatgacgacaagttttgtgaccgtcattcaaagacccactt |
| caaatggcctttaattcgtcataaatctgaagatattagttcttcaggttctttcgttca |
| acctaggtacaaattcagttatgaaacggactgtaatgttaaacaaatcgaatttacaaa |
| gccgtcagatttaatccgtctcaatggtctagacggtcagacacaaacattcaattttag |
| tacagcttttaatttttcccaaaccaagtctgtcgatcaagatgctagtaataactacaa |
| aacaattttcac |
| >TCONS_00015344 gene=XLOC_009585 |
| accccttacgttctaagtgtaactcatacctacgtacttgatgaatctgttgatatacta |
| tacataataaaatttatgcttttttgtcacagattttcccgaattctattcggtttatta |
| aaataattttgaactgaatcgaataaatgacttccagaagagaaatcctgacactttaca |
| aaaatctcttaacatatgtgaataacctaaagcattccgataaaagttatctaaaaaaga |
| ggattcagtctacttttaaagagaataaagtgtctactgatgatgatgaaacaacacgat |
| tgtataagaaaggttgtgagattctacgacttaaaagatttatctaaattttgcatcggt |
| gtacatttcccttcgtgatttcattcagttcatgtaatttgctgtttttccagtgattta |
| ttttcccaaat |
| >TCONS_00015736 gene=XLOC_009825 |
| tttggattttgttttcgctgcacgtatgcgataatacatcagatccttctcttccttgga |
| atacattgaaaatggattcgattttaagaaatttaaagtactcaagggacgcacttactc |
| ccttaataagtttggcgaagacatggcgacgaccagctgcagcttggaccgttaccgctg |
| gtgtgctattcgtttattttaccgattggaaagtgatatgcactcgaattccactttata |
| atcgtaaattcttggattcagatgagtgaaacattgtttagcttcgaagcgacccgaaga |
| cataaaaataaggaagcacacagttcctcagaaattctttcactcaactagctgat |
| >TCONS_00016787 gene=XLOC_010517 |
| ataatttaacattatgactggattaattttaattttctccttgctattaattctgtcgtc |
| aaatatagaaggtaaatgtaatgaagaaggtgaagcatgtagcaaaacattgggaactcg |
| ttgttgtggtgaattatattgtaaactattcaaaccacttaaaggtagatgtaccaatcc |
| aaaacgtagacattcaacaatctggtagactactatgtttaacctttgctaaaaatatca |
| ttcaccttgccaatatgttgagcaattaaat |
| >TCONS_00017317 gene=XLOC_010860 |
| gagattcctcaaggaatccacacggggttactaccattaaaacaagtcaaagcaactgtt |
| ccttcagtttgtgtatatccaccaaataaatcacatagtgataataatattcctatttca |
| cagcaaacattgacgaatacagcgaatcctgacttgattacgaattgtaatgttgcatca |
| accgtgacattaagtacggtgtgcaatgtaactgtgtcgtcgacattcgtgtctaaaaca |
| acaacaacaacaacaacgacagctattacaaaatcaaaagcagttgttacggctgatcct |
| ttgaaaaatcactcgaccaatcaaatgattgatactaatgaaaacaaatctcaactgaat |
| catgattgtcttcaatgtgacaataataatcatcgtacagtcaatgtactacagtgtgga |
| ttgcctcctgactatcttcaaactcagcattatccagttactgttactaccattactact |
| aatactgtaactactgc |
| >TCONS_00017348 gene=XLOC_010880 |
| gatgcaacgtgcatctcattggtctactgaagtagagaatgcatataggtttcagttggc |
| gggttacagagatgaagtggaatattttaactataataatgctgacccagagaaatggac |
| gaatacgggatttgttaaaaaattgaagcgacgagatggtctgttctactattttaacaa |
| gaatagggagtgttcagacaaagatataccgaaatgcaaactatatgtatattaataaac |
| aaaatataactcagtgaataatatccaccaaactttatttctagaagtttttcacagtgc |
| atactccgtccaacaaaggatgtgaaaatttgacgcgagtatgaaccagcaa |
| >TCONS_00018007 gene=XLOC_011311 |
| gtggacgccacatcctaagtctgctggccttactacgatagttgtccactcttcaatcaa |
| gaggaaaaatccccgaagacctcgtccctatcgtgaccagggggttaaatttcttgttca |
| ggtttaccatgaagaaaatcgtggaatggctccttgttttatctttaatttccgcaatat |
| gggtatctaaattaatggggattatcactgtacagtctgactgcggcaacataatcctca |
| actggttgccttttcatatattattcatttttggaacagtttctgtgttaattatacttt |
| atcgtacgtacagttttaatgattgtcccgaggcgtccacggagcttatgaagctagtca |
| acgaagcgaaacgggatctcacttaccgcggatttgtttttgaatcttaattactaaaga |
| acataaacttagataattttgcactgtgactaacattttgtgatgttaatttgtcagtac |
| gtaagttgtgtagcctcacttctgtgactacagtatagaaacattttgtaccatc |
| >TCONS_00018008 gene=XLOC_011311 |
| tggacgccacatcctaattactacgatagttgtccactcttcaatcaagaggaaaaatcc |
| ccgaagacctcgtccctatcgtgaccagggggttaaatttcttgttcaggtttaccatga |
| agaaaatcgtggaatggctccttgttttatctttaatttccgcaatatgggtatctaaat |
| taatggggattatcactgtacagtctgactgcggcaacataatcctcaactggttgcctt |
| ttcatatattattcatttttggaacagtttctgtgttaattatactttatcgtacgtaca |
| gttttaatgattgtcccgaggcgtccacggagcttatgaagctagtcaacgaagcgaaac |
| gggatctcacttaccgcggatttgtttttgaatcttaattactaaagaacataaacttag |
| ataattttgcactgtgactaacattttgtgatgttaatttgtcagtacgtaagttgtgta |
| gcctcacttctgtgactacagtatagaaacattttgtaccatc |
| >TCONS_00018009 gene=XLOC_011311 |
| tagtgattgtaacatatgtcttacgtgatttactacgatagttgtccactcttcaatcaa |
| gaggaaaaatccccgaagacctcgtccctatcgtgaccagggggttaaatttcttgttca |
| ggtttaccatgaagaaaatcgtggaatggctccttgttttatctttaatttccgcaatat |
| gggtatctaaattaatggggattatcactgtacagtctgactgcggcaacataatcctca |
| actggttgccttttcatatattattcatttttggaacagtttctgtgttaattatacttt |
| atcgtacgtacagttttaatgattgtcccgaggcgtccacggagcttatgaagctagtca |
| acgaagcgaaacgggatctcacttaccgcggatttgtttttgaatcttaattactaaaga |
| acataaacttagataattttgcactgtgactaacattttgtgatgttaatttgtcagtac |
| gtaagttgtgtagcctcacttctgtgactacagtatagaaacattttgtaccatc |
| >TCONS_00018307 gene=XLOC_011493 |
| tggtcacaattacttcatttaataaatacacgtgaagatgatttaaaagcaagagaatcg |
| tttaataattcctatgcacaagcacgtaatcaagtacaagaaatgttgttaattgctgat |
| ggacgtttaactactttatcatcacctatcactttaaatttgaattcagtaaaacaacaa |
| atggacaaattaaatgaattatattcactccgagagacaatcaaacaacaattagatgaa |
| gtggaccaattaggctctgcttatgatactttattacatacaccaagtggaattgataag |
| aatttcggacgtggaacaacttcatcaaatgatgttcacatttcttcttcttcagcggta |
| ccacgtgtatccacttctgctgcctataaacaatcaaaagacaataaattaatgaatgta |
| ttaagtccattggctagtagtggatcaagtggcatctccagtgccgatcctatgtgtaat |
| ctacatgaaattaatgaagtcaatcgtgaattagacgagctacatgaacgttatgatcaa |
| ttaggta |
| >TCONS_00018807 gene=XLOC_011833 |
| atgatgaaccaaaaccaccattcgttcgttcacagcttcatcatgtcaatgtggaaggaa |
| ttcttcatgagccaaagatagttagttctggaacgtctgctgacatagaggtctacaaaa |
| tgggaaatactactaaagctcaccaaacagagatgattatgaatgttttgttatcaagta |
| caaatgcagaacgacaaaatattatgcatcagtacaacaggattttaaaaaagcccctat |
| tgaacgaaaaagaaaacattaaatcgggattaatgtatcagttatttgaagacctactaa |
| cagacacatctattttattagctgatgaattgtacagagcaataatgtcttccgatttac |
| ggagaacgacaagtatactgattgatttctggggtgatgaatttgatcaagtagaaaatg |
| cttataaaatatattctgtagaaccattctggaagtctatagagaaacactttggaaaat |
| ctgtaaaaagtactttacattgtatagttgaaacaaggaaaaatgaacctaaacaagaag |
| atccaataaaaggtcgaggtgttaaaccgatagttaacaaaacagcagttagtgaggtgt |
| tttatgacataatggtcgtgttggattctaacaaagatgtaggacagcaacttggtcaac |
| gtctatgtaaacttgatccatttcaatttcaaagattgaatatgatttataaacgaaaac |
| ccgttagacaatttggtgaagttcttgaaagtaggacgtctggtcaactgcataatattt |
| tagtagcaatgtataattattctattaacaaaccaatgtactttgcaacgttaattcatg |
| atgagcttcataaagaactcattgatgttctaggtgttcaacgtttcctacttttccgtt |
| ctgaagtaagtttatacagattacgtaaaacaaaatttcaatgttaacatttattcaatt |
| tattttgacggaagtataaaagtgagagaactattcgaatcttgataatttgcatctaca |
| tagattcacatatctttcaaaccatgtgaatcatatagtaaacacatttttctggggttg |
| acaaataatcatttgtgctgtaatgaattcgacagtctagaaggtaaaaagagaggggaa |
| tacctagtgagcggattcatgtcagctattgaatttcagatccatgtttctgtggcttca |
| tgtttatgctttaaaacagaggttttgtttttggaccctagtaggtttagagcgaatttt |
| agacaagaggtgataattcagcatctacaagaaaagtgatcataagattttggcgctaaa |
| ttcaattatccatttggataaatagagttttggcaatatagctgatgtcagttcgtgatg |
| aaaaccccaagtaaaattcctagccttaaccatgaaccgtaaatcataatttgaattcct |
| cacaggtttataggcctcattttgtcttagttattatgttgacactctcagtatcactct |
| agcgtcgctcataggtcgtccataacttatggtctcacccttaaccctaaaccctaaacc |
| ttaactctaaccctaaaccgtaattcataccaatataccaacaacatagaagctatgtgg |
| tcgaggctaaaagaattttttagactttatcatcgtttcagaggtcaactactatggagc |
| catatggatgtgttcctgtaccgtatgcactatgattttagaacttttgaacctaaatct |
| gaccttgagaagttttaagtcatgtagaagaacagtatctcctttgatttttgagttttg |
| tattatttatttcactgtatactaacagtcttctgattagctaatatttctcaaggtcat |
| ttaagattcgttcaaaggtcggcagtatagtttagtctcgccggtttatcttttttacat |
| ggtctcaatacagaagctaagtgaatccagttttcaacctgtttacatatcactaataat |
| ttcaaatgatgagattcatatgatcaatatattgttctaagctggtgaataatttttgtc |
| cactgacaaattcgatattctagaacgttaacaaatgagttaacacagatttcgaacttc |
| gcgagttacttaaacctttgaactgattatttctcctaccacctaaatatttcgacaagt |
| tatcttttcttgtatttacatatcattagagtcaaatcacttaacgatgttaaaaattac |
| aatccgagacttcaggatcgctgggaatctgcgttgaagagaataacagttaatcaagta |
| tcgattctcaaactgttatttttcaaattcccattattaaatggtatatcttaaatcaat |
| actaaatgcaaacatgtaacgtatatgtggttcaaacataattccagaatcataattgaa |
| tgacttatatgacgattatttagtattgtcgtgtggttacttattttcacataagtagta |
| tatggtgtaagtcaggcatagaatgtattttggcagatgctcgattacaaaagaatagga |
| acgaagcacaatcagtatgaaaatgtatgaatgactgatatttgcagaaggaacagtcaa |
| gattgagacaattgatggttattttgcaagttaactgtcaactgtatggttatcagattt |
| ttgtgagacagtctataatttttgcttaaatacattcgattgtccccgcccgtgttctca |
| ttcactacagtattgccacatgtcttatatgtttgtcatcagcagcgatcaggcaataag |
| aaaacgtacggcatcagtgtagcctaatttgcaaatagtgttaatcaaaatctgtgtttt |
| aagtccttagacgacagtctcaagtgtttaacacacttgaataaatacaactatgtttat |
| tttatcgatagttgtgtgactctggactgcataccatatgcacttactgttttcctttat |
| gaatcactacttcgttgttactgcctctaatggtttacttgaaactatacgtggcaatct |
| aaatatatatgaatataactttctatcattaatgtctaatcaaaatgtgactcattttct |
| cgattaatcctcatcagagttaaactttcattattgatgtaggaatatttgtgacatttg |
| atgtgctccatcgcttattattgatcattgttattctaaatctactccatcaatatggca |
| agcttagaaagagaatattaaaacatcaaatagaggaagttcaatacaggaattactcaa |
| aataaaatgtccaagatctgaaaaatatactacgtacgtcaaactgagattcattcttac |
| ttacttacttacatacacctattacccctcgtcaaggagcataggccgctcaccagcatt |
| gtccatccaactctgtcctgagccttcctttccagttctttccagttattattcatcctt |
| ttaatatctgcttctatttcctgacgtaatgtgttcttcggcctttctcttttccgcttc |
| ccttccggattccaagtaagggattgccttgtaatgtacattggtgatttccgtaatgta |
| tgtccgatccacttccaacgtcttttcctaatttcctcatcagttggaagctgatttgtc |
| ctctctcacaaataacttattcgtaaacacaattaatgttaacacgtattctgcattctg |
| ctttgacatttacttttctactgagactattgtagaacgttttgaccaaacaaacattcc |
| tgcaattaagcagacgtcataataaaaagaaacaatttacaaatagagaaacttcgctga |
| agttgaaaagccaaatgatctgacgaaccaagaaataaaatacgtgaatccagtgtctac |
| taagaaactcttatctcgtagaaacttttcagttaggtgggaaatacgataatgtcaatg |
| tttagttgaaaaatttggaacttgaacaaaatttttataagcctatcctccgatttcatg |
| ctttcgtacccgcaagttttaagaagagttgtatctcaaattgtactgaaattgaggact |
| aaaaatgaatacagtctcttaaggtgtctcaatgtttatgacctgaagaaattccaaacc |
| tctttcttgagaagtgcgctgacattctgtgcgatcgatacaacagcagttttaacgaat |
| cattttatgccaaaaatgtgaagaaagattgcagataattccgataacaaagaaagtatt |
| tagaggtgaaaaggctaaattgagacaatagcagtaactaatttttgtcttggttgtttg |
| aatgttctcattcacgtttgagacttcaattgatcagcctcttattggcatgtgtgcatt |
| ctatacggattgtcttgatattgcactggagtgaacatcaaatcggagatgcaggtacat |
| ccaggtgacaagttttaaacagaatggatcgcgcattgtgagttccactactagccacca |
| ttcatctctgcttgaaatagcaatagatttaattttactcaagactatcggaacaccatt |
| tgtaccaatgcttaagccagtaataaaagagttatacccatccatattaaattatttaca |
| aataaaaaatacctgccctgaatactgctgttgttttgcatcataatattatgttcggtt |
| tggaaaagcgtatgaagtatgttctatgctcgtttctatacgatatctcttgatttgact |
| ttcaaggtgggtcacttcaataaggatagctggttaactggttgaccatgtttttatttc |
| gctgggaaaaaaacattattctatgttcagtggaaagtgtttttaatcaatagttaaggt |
| ctgctagaaggaactgattttcttcttaacgttttcatgcttttattctgcgtgacgtac |
| tggttatagagaacagttttatcagttttatgggtgatctcactgtatgtgaaccaattt |
| cagtctctttaggttctttagtaatgaatgagtttcccttaaatgatgttcttttccaaa |
| tattttgacagctaatccttctaaatgtcaaagtaacaatttattcctgaaaccagagca |
| aaacataaacaaactattggaatccgataaaagttgtactattccagatcgtgtacaaaa |
| accgtcgaaggtcttttatcttggtgcaatgatcccgtcttatctctcttggtcttctca |
| tgttttcctgttatcgatgaaaattttccgtgtgacttactatataaacgagctacgcgt |
| ttttcacataagtggacatttagttttacgatttgtaggttttcgtatactactcataat |
| tctttaatgttcttcattattctttccttttgaaaaaagatttttgtttattgcgcaaac |
| tgctgaaggcagctttcagggtgtgtaataaatcttttgaggttattattatagttctaa |
| ataaatatttaaaatcatgcaaactcctgtcaggtgttatctaatcagctactattcatc |
| cactacattcttatcttagttcttgtatatcttctggtggaacgaaacgataatatatca |
| gaatccatacactcaagcgaaggtataaatacctccaaatgccctggtacggccaagagt |
| agtgagagtccgctctccctttcgaaatgttctcacatggccacgagtatatagcctctg |
| ccagcgaagtcctactcactgccttctcgcaacacgggtgttgtttacgaaatcgagagg |
| acgaacagcgaacggccggcgctttaatcgagttggtggacacggaaagtccacctaggg |
| ggatttgaaaaaccctgattccaaaccaatggtgcacatgggctccaatattctacattt |
| tttttacttctatggaacgaataagccatctaacagatattcatcgacaatttcaatgga |
| tttacagttttcatacaacttctattaaatcactgtatctgtgtaaagattgaactaaca |
| ttccacgttcagggctaattttaacattgatagggtagatacatcattattggcttgtaa |
| gtaccgttttgtatgttcagacctccgagcgaatcgttcactttattccgatatatatca |
| tttgaattaaaggttggattacattttgtacctaatattggttattttattgagaatggt |
| tataaattaccgtttatctacgtttttgattcagaaggagttttgtggagatttcagtat |
| tttggtcgttgaaatcatgagtcaattggagctacaccaccatgaaaacctggaaacact |
| ggaaggccatttcgtcctattatgggactcctcagtagtgcgttttgtcttgtatt |
| >TCONS_00018808 gene=XLOC_011833 |
| atgatgaaccaaaaccaccattcgttcgttcacagcttcatcatgtcaatgtggaaggaa |
| ttcttcatgagccaaagatagttagttctggaacgtctgctgacatagaggtctacaaaa |
| tgggaaatactactaaagctcaccaaacagagatgattatgaatgttttgttatcaagta |
| caaatgcagaacgacaaaatattatgcatcagtacaacaggattttaaaaaagcccctat |
| tgaacgaaaaagaaaacattaaatcgggattaatgtatcagttatttgaagacctactaa |
| cagacacatctattttattagctgatgaattgtacagagcaataatgtcttccgatttac |
| ggagaacgacaagtatactgattgatttctggggtgatgaatttgatcaagtagaaaatg |
| cttataaaatatattctgtagaaccattctggaagtctatagagaaacactttggaaaat |
| ctgtaaaaagtactttacattgtatagttgaaacaaggaaaaatgaacctaaacaagaag |
| atccaataaaaggtcgaggtgttaaaccgatagttaacaaaacagcagttagtgaggtgt |
| tttatgacataatggtcgtgttggattctagcaaagatgtaggacagcaacttggtcaac |
| gtctatgtaaacttgatccatttcaatttcaaagattgaatatgatttataaacgaaaac |
| ccgttagacaatttggtgaagttcttgaaagtaggacgtctggtcaactgcataatattt |
| tagtagcaatgtataattattctattaacaaaccaatgtactttgcaacgttaattcatg |
| atgagcttcataaagaactcattgatgttctaggtgttcaacgtttcctacttttccgtt |
| ctgaagaatatttgtgacatttgatgtgctccatcgcttattattgatcattgttattct |
| aaatctactccatcaatatggcaagcttagaaagagaatattaaaacatcaaatagagga |
| agttcaatacaggaattactcaaaataaaatgtccaagatctgaaaaatatactacgtac |
| gtcaaactgagattcattcttacttacttacttacatacacctattacccctcgtcaagg |
| agcataggccgctcaccagcattgtccatccaactctgtcctgagccttcctttccagtt |
| ctttccagttattattcatccttttaatatctgcttctatttcctgacgtaatgtgttct |
| tcggcctttctcttttccgcttcccttccggattccaagtaagggattgccttgtaatgt |
| acattggtgatttccgtaatgtatgtccgatccacttccaacgtcttttcctaatttcct |
| catcagttggaagctgatttgtcctctctcacaaataacttattcgtaaacacaattaat |
| gttaacacgtattctgcattctgctttgacatttacttttctactgagactattgtagaa |
| cgttttgaccaaacaaacattcctgcaattaagcagacgtcataataaaaagaaacaatt |
| tacaaatagagaaacttcgctgaagttgaaaagccaaatgatctgacgaaccaagaaata |
| aaatacgtgaatccagtgtctactaagaaactcttatctcgtagaaacttttcagttagg |
| tgggaaatacgataatgtcaatgtttagttgaaaaatttggaacttgaacaaaattttta |
| taagcctatcctccgatttcatgctttcgtacccgcaagttttaagaagagttgtatctc |
| aaattgtactgaaattgaggactaaaaatgaatacagtctcttaaggtgtctcaatgttt |
| atgacctgaagaaattccaaacctctttcttgagaagtgcgctgacattctgtgcgatcg |
| atacaacagcagttttaacgaatcattttatgccaaaaatgtgaagaaagattgcagata |
| attccgataacaaagaaagtatttagaggtgaaaaggctaaattgagacaatagcagtaa |
| ctaatttttgtcttggttgtttgaatgttctcattcacgtttgagacttcaattgatcag |
| cctcttattggcatgtgtgcattctatacggattgtcttgatattgcactggagtgaaca |
| tcaaatcggagatgcaggtacatccaggtgacaagttttaaacagaatggatcgcgcatt |
| gtgagttccactactagccaccattcatctctgcttgaaatagcaatagatttaatttta |
| ctcaagactatcggaacaccatttgtaccaatgcttaagccagtaataaaagagttatac |
| ccatccatattaaattatttacaaataaaaaatacctgccctgaatactgctgttgtttt |
| gcatcataatattatgttcggtttggaaaagcgtatgaagtatgttctatgctcgtttct |
| atacgatatctcttgatttgactttcaaggtgggtcacttcaataaggatagctggttaa |
| ctggttgaccatgtttttatttcgctgggaaaaaaacattattctatgttcagtggaaag |
| tgtttttaatcaatagttaaggtctgctagaaggaactgattttcttcttaacgttttca |
| tgcttttattctgcgtgacgtactggttatagagaacagttttatcagttttatgggtga |
| tctcactgtatgtgaaccaatttcagtctctttaggttctttagtaatgaatgagtttcc |
| cttaaatgatgttcttttccaaatattttgacagctaatccttctaaatgtcaaagtaac |
| aatttattcctgaaaccagagcaaaacataaacaaactattggaatccgataaaagttgt |
| actattccagatcgtgtacaaaaaccgtcgaaggtcttttatcttggtgcaatgatcccg |
| tcttatctctcttggtcttctcatgttttcctgttatcgatgaaaattttccgtgtgact |
| tactatataaacgagctacgcgtttttcacataagtggacatttagttttacgatttgta |
| ggttttcgtatactactcataattctttaatgttcttcattattctttccttttgaaaaa |
| agatttttgtttattgcgcaaactgctgaaggcagctttcagggtgtgtaataaatcttt |
| tgaggttattattatagttctaaataaatatttaaaatcatgcaaactcctgtcaggtgt |
| tatctaatcagctactattcatccactacattcttatcttagttcttgtatatcttctgg |
| tggaacgaaacgataatatatcagaatccatacactcaagcgaaggtataaatacctcca |
| aatgccctggtacggccaagagtagtgagagtccgctctccctttcgaaatgttctcaca |
| tggccacgagtatatagcctctgccagcgaagtcctactcactgccttctcgcaacacgg |
| gtgttgtttacgaaatcgagaggacgaacagcgaacggccggcgctttaatcgagttggt |
| ggacacggaaagtccacctagggggatttgaaaaaccctgattccaaaccaatggtgcac |
| atgggctccaatattctacattttttttacttctatggaacgaataagccatctaacaga |
| tattcatcgacaatttcaatggatttacagttttcatacaacttctattaaatcactgta |
| tctgtgtaaagattgaactaacattccacgttcagggctaattttaacattgatagggta |
| gatacatcattattggcttgtaagtaccgttttgtatgttcagacctccgagcgaatcgt |
| tcactttattccgatatatatcatttgaattaaaggttggattacattttgtacctaata |
| ttggttattttattgagaatggttataaattaccgtttatctacgtttttgattcagaag |
| gagttttgtggagatttcagtattttggtcgttgaaatcatgagtcaattggagctacac |
| caccatgaaaacctggaaacactggaaggccatttcgtcctattatgggactcctcagta |
| gtgcgttttgtcttgtatt |
| >TCONS_00018871 gene=XLOC_011888 |
| caaatggatattttgagatgttgggtcgcgtcgcgttgttttcaggagccaggaagtatt |
| gtagcgcacagtcaaactatatgttaaatgagttgtgcatactcgttgacaagtgtgatc |
| atgttcttgggtttgctaacaagaagtattattcggaaaatcattactacatcgggcctt |
| cagcttattcctttttcaagaggatgctagtagtgaaacaaatcacagaagcctcaagct |
| actagttcagaagcgttcatctagtaaacttaccttcccatctctgtggtcaaatacttg |
| ttgcagtcatccaatcatgaattttcctgatgaacttattgagttggatgcagtcggggt |
| gaaaaaagcagcacaaagaaaggtcgagtgctgtatgcagcaccaaatgaaccatgtacc |
| caaac |
| >TCONS_00018877 gene=XLOC_011892 |
| aaattatttattgactttgagttccagtggcttgccaataatgatgtttactgcatgtgc |
| aatattttccagctttcttctctgtacatacgctgtgacgctggaagaaggacttaagaa |
| cccttccaagtatattcgttatgacacagctccaaataatacttggattcatgctctgat |
| ttctctttgcataacgtacggtacgcttactggatttatactttgcatacacctagtggt |
| atacctatcaggatcaaagaaaaatcgtcgttctgcttgataaatcaactaattagtata |
| aatgctacatgtagttcttcatgttaacattgtattttattagccatatttgcctaattc |
| at |
| >TCONS_00018878 gene=XLOC_011892 |
| aaattatttattgactttgagttccagtggcttgccaataatgatgtttactgcatgtgc |
| aatattttccagctttcttctctgtacatacgctgtgacgctggaagaaggacttaagaa |
| cccttccaagtatattcgttatgacacagctccaaataatacttggattcatgctctgat |
| ttctctttgcataacgtacggtacgcttactggatttatactttgcatacacctagtggt |
| atacctatcaggatcaaagaaaaatcgtcgttctgcttgataaatcaactaattagtata |
| aatgctacatccatatttgcctaattcat |
| >TCONS_00018881 gene=XLOC_011894 |
| ttacggtcctcttaaaatgctaaagtacgtttactataagatcctgcgctacccgtcacg |
| ttttgtcggagctgctgtcgcatccgctttcgcctttgaatttttgttttttaacggtct |
| ggacaagatatattttcatgtaaacaaagggttactgtttaaagatgttatggcttccat |
| aaaacagaaggaagaagaagaatgaaatcctattttttatcttcttgtagtcaacttttc |
| ctatattatttgcaatgtctagccgataccct |
| >TCONS_00018892 gene=XLOC_011904 |
| aaactggtttggcttttctgccgtgtctgcgatttctttgttcattctgattaacatttc |
| gtcttatacaaatttctgcttattgtaaacagtgagaaacacttcacttagactgtttac |
| agcggagacatgtcgctgatacttgaactccggttcaggtctgtttattcatgcttcctg |
| tcgtgtgggattagcaccttgctgttggtgcttatggtgttttcacaacacataggcctt |
| tgtgctccttggttttggatatcagatacgtttcttgacctttttatcacgtcgaaaatc |
| ttcttgttaatattcaatgttataattattggtttgacaacactcatgtttcgggctaga |
| ctgtcagtaacggatcgtgttttccgtaatatatatcagatatgtggggctttcttcaac |
| tggcgtgattttaaaatccttttttctagcatgatgctgggtgcaataaatagtttggtt |
| gtagcaaaactcctagcaactgggaagtatgggtcatttttcgaatacaatcattttcct |
| tccgtgaaccaaagagcgcttatactaacaacttgtggagcatttatgggtttgctgttt |
| tctattacagtgctcgtcatgaataaactgacgcttaactactcatgtccaattgtaagt |
| tctcacaaaatacccatattgtagggcaacatattcgtgataacaaaggagcatatgttt |
| actcgtctactcacaactgtcgggagagtattatatttcttgccactctacgcagtatgc |
| tcaaatctgttttggcatccccaatggctctcctttatttggcttattttggatattcga |
| ctaattcttttgattctgatcacaacctttcacctccagttttcatggagttgtgcaatc |
| gatgtcctaaagtcacagtttcagaaacctatcgaaatccctttgtcctctgtccttgac |
| ccttccagttcagtccaaggcgttcttagtggttgcgctaatcctttagaacaagtaagt |
| tgacatcttgacgatcattttgtagcatttgactttggaaagattggctgatttggtggc |
| ta |
| >TCONS_00018893 gene=XLOC_011904 |
| actcctagcaactgggaagtatgggtcatttttcgaatacaatcattttccttccgtgaa |
| ccaaagagcgcttatactaacaacttgtggagcatttatgggtttgctgttttctattac |
| agtgctcgtcatgaataaactgacgcttaactactcatgtccaattggcaacatattcgt |
| gataacaaaggagcatatgtttactcgtctactcacaactgtcgggagagtattatattt |
| cttgccactctacgcagtatgctcaaatctgttttggcatccccaatggctctcctttat |
| ttggcttattttggatattcgactaattcttttgattctgatcacaacctttcacctcca |
| gttttcatggagttgtgcaatcgatgtcctaaagtcacagtttcagaaacctatcgaaat |
| ccctttgtcctctgtccttgacccttccagttcagtccaaggcgttcttagtggttgcgc |
| taatcctttagaacaacatttgactttggaaagattggctgatttggtggcta |

**Supplementary Table S4:** Set of 15 expressed lncRNAs with the neighboring coding genes and their respective GO entries

| LncRNA name | Transcript ID | Length | Genomic neighbourhood | Smp_gene | GO: Biological Process | GO: Molecular Function | GO: Cellular Component |
| --- | --- | --- | --- | --- | --- | --- | --- |
| Sm-lncRNA 1 | TCONS_00001011 | 570 | 20861 bp at 5' side: putative yeast nucleolar complex | Smp_154980 | - | - | GO:0005634 - Nucleus |
|  |  |  | 36025 bp at 3' side: hypothetical protein | Smp_086060 | - | - | - |
| Sm-lncRNA 2 | TCONS_00012347 | 556 | 6900 bp at 5' side: hypothetical protein | Smp_193940 | - | - | GO:0005737 - Cytoplasm |
|  |  |  | 1354 bp at 3' side: chromosome transmission fidelity protein 8 | Smp_130740 | GO:0007049 - Cell cycle/GO:0006260 - DNA replication | GO:0003677 - DNA binding | GO:0005634 - Nucleus |
| Sm-lncRNA 3 | TCONS_00013257 | 742 | 13223 bp at 5' side: putative pdz and lim domain protein | Smp_022340 | - | GO:0008270 - Zinc ion binding | GO:0005737 - Cytoplasm/GO:0005856 - Cytoskeleton |
|  |  |  | 5551 bp at 3' side: putative dnaj homolog subfamily B member 2, 6, 8 | Smp_022330 | GO:0009408 - Response to heat/GO:0006260 - DNA replication/GO:0006457 - Protein folding | GO:0008270 - Zinc ion binding/GO:0005524 -ATP binding/GO:0031072 - heat shock protein binding/GO:0051082 - unfolded protein binding | GO:0005737 - Cytoplasm |
| Sm-lncRNA 4 | TCONS_00003004 | 309 | 6057 bp at 3' side: histone lysine N methyltransferase MLL5 | Smp_246410 | GO:0006355 - Regulation of transcription, DNA-templated/GO:0016568 - Chromatin modification/GO:0006350 - Transcription | GO:0018024 - Histone-lysine N-methyltransferase activity | GO:0005634 - Nucleus/GO:0005694 - Chromosome |
|  |  |  |  |  | - | - | - |
| Sm-lncRNA 5 | TCONS_00000625 | 202 | 15028 bp at 5' side: proteasome subunit beta 1 (T01 family) | Smp_025800 | GO:0006511 - Ubiquitin-dependent protein catabolic process | GO:0008233 - Peptidase activity/GO:0004298 - Threonine-type endopeptidase activity | GO:0005829 - Cytosol/GO:0005839 - Proteasome core complex/GO:0005634 - Nucleus/GO:0005737 - Cytoplasm |
|  |  |  | 3974 bp at 3' side: unnamed protein product | Smp_200530 | - | - | - |
| Sm-lncRNA 6 | TCONS_00001840 | 278 | U3 small nucleolar ribonucleoprotein | Smp_102820 | GO:0006412 - Translation | GO:0019843 - rRNA binding/GO:0003735 - Structural constituent of ribosome/ | GO:0015935 - Small ribosomal subunit/GO:0005622 - Intracellular/GO:0005840 - Ribosome |
|  |  |  | fibroblast growth factor receptor a | Smp_175590 | GO:0006468 - Protein phosphorylation/GO:0007169 - Transmembrane receptor protein tyrosine kinase signaling pathway | GO:0005524 -ATP binding/GO:0004714 - Transmembrane receptor protein tyrosine kinase activity/GO:0004674 - Threonine kinase activity/protein serine | GO:0016021 - Integral component of membrane/GO:0016020 - Membrane |
| Sm-lncRNA 7 | TCONS_00009100 | 659 | 7028 bp at 5' side: putative histone H2A | Smp_130880 | GO:0006334 - Nucleosome assembly | GO:0003677 - DNA binding | GO:0005634 - Nucleus/GO:0005694 - Chromosome/GO:0000786 - Nucleosome |
|  |  |  | 9619 bp at 3' side: ATP synthase E chain | Smp_015980 | GO:0015986 - ATP synthesis coupled proton transport | GO:0046872 - Metal ion binding/GO:0015078 - hydrogen ion transmembrane transporter activity | GO:0016020 - Membrane/GO:0000276 - Mitochondrial proton-transporting ATP synthase complex, coupling factor F(o) |
| Sm-lncRNA 8 | TCONS_00009852 | 1157 | 5857 bp at 5' side: rRNA | Smp_sma.5s-14.1 | - | - | - |
|  |  |  | 2437 bp at 3' side: putative suppressor of actin (sac) | Smp_060420 | - | GO:0004437 - Phosphatidylinositol phosphatase activity/GO:0004439 - phosphatidylinositol-4,5-bisphosphate 5-phosphatase activity | - |
| Sm-lncRNA 9 | TCONS_00009849 | 2098 | 27717 bp at 5' side: hypothetical protein | Smp_193460 | - | - | - |
|  |  |  | 53702 bp at 3' side: rRNA | Smp_sma.5s-14.1 | - | GO:0004437 - Phosphatidylinositol phosphatase activity/GO:0004439 - phosphatidylinositol-4,5-bisphosphate 5-phosphatase activity | - |
| Sm-lncRNA 10 | TCONS_00009851 | 875 | 27717 bp at 5' side: hypothetical protein | Smp_193460 | - | - | GO:0030054 - Cell junction/GO:0045211 - Postsynaptic membrane/GO:0045202 - Synapse/GO:0005737 - Cytoplasm |
|  |  |  | 53702 bp at 3' side: rRNA | Smp_sma.5s-14.1 | - | GO:0004437 - Phosphatidylinositol phosphatase activity/GO:0004439 - phosphatidylinositol-4,5-bisphosphate 5-phosphatase activity | - |
| Sm-lncRNA 11 | TCONS_00012478 | 1591 | 47352 bp at 5' side: putative 40s ribosomal protein S9 | Smp_180000 | GO:0006412 - Translation | GO:0019843 - rRNA binding/GO:0003735 - structural constituent of ribosome | GO:0015935 - Small ribosomal subunit/GO:0005622 - Intracellular/GO:0005840 - Ribosome |
|  |  |  | - |  | - | - | - |
| Sm-lncRNA 12 | TCONS_00010393 | 237 | 9284 bp at 5' side: putative diphteria toxin resistance protein 2, dph2 | Smp_174680 | - | - | GO:0005737 - Cytoplasm |
|  |  |  | 4506 bp at 3' side: Ubiquitin conjugating enzyme E2 J1 | Smp_174670 | GO:0043687 - Post-translational protein modification/GO:0006512 - Obsolete ubiquitin cycle/GO:0051246 - regulation of protein metabolic process | GO:0004842 - Ubiquitin-protein transferase activity | - |
| Sm-lncRNA 13 | TCONS_00010903 | 1166 | 1571 bp at 5' side: putative flagellar radial spoke 3 protein | Smp_170010 | - | - | - |
|  |  |  | 32039 bp at 3' side: putative ubiquitination factor E4a | Smp_030780 | GO:0006511 - Ubiquitin-dependent protein catabolic process/GO:0016567 - Protein ubiquitination/GO:0006512 - Obsolete ubiquitin cycle | GO:0034450 - Ubiquitin-ubiquitin ligase activity/GO:0004842 - Ubiquitin-protein transferase activity | GO:0000151 - Ubiquitin ligase complex/GO:0005737 - Cytoplasm |
| Sm-lncRNA 14 | TCONS_00011021 | 2280 | 7796 bp at 5' side: venom allergen-like (VAL) 7 protein | Smp_199890 | GO:0009405 - Pathogenesis | GO:0008200 - Ion channel inhibitor activity | GO:0005576 - Extracellular region |
|  |  |  | 42906 bp at 3' side: hypothetical protein | Smp_156240 | - | - | GO:0016020 - Membrane |
| Sm-lncRNA 15 | TCONS_00013835 | 664 | 8674 bp at 3' side: ubiquinol cytochrome C reductase | Smp_061870 | GO:0055114 - Oxidation-reduction process/GO:0006810 - Transport/GO:0006118 - Obsolete electron transport/GO:0022900 - Electron transport chain | GO:0008121 - Ubiquinol-cytochrome-c reductase activity/GO:0009055 - electron carrier activity/GO:0005506 - iron ion binding/GO:0051537 - 2 iron, 2 sulfur cluster binding | GO:0005739 - Mitochondrion/GO:0016021 - Integral component of membrane/GO:0016020 - membrane/GO:0005746 - mitochondrial respiratory chain |
|  |  |  | - |  | - | - | - |
